# Molecular Engineering of a Conductive Metal–Organic Framework for Ultrasensitive, Rapid, Selective, and Reversible Sensing of Nitric Oxide

**DOI:** 10.1021/jacs.5c07229

**Published:** 2025-08-01

**Authors:** Joseph Y. M. Chan, Elissa O. Shehayeb, Doran L. Pennington, Christopher H. Hendon, Katherine A. Mirica

**Affiliations:** a Department of Chemistry, Burke Laboratory, 3728 Dartmouth College, Hanover, New Hampshire 03755, United States; b Department of Chemistry and Biochemistry, 3265 University of Oregon, Eugene, Oregon 97403, United States

## Abstract

The selective, sensitive, low power, and portable detection of nitric oxide (NO) is important for environmental monitoring, industrial safety, and medical diagnostics. While tremendous progress has been made in detecting NO, existing technologies exhibit significant trade-offs in sensitivity, selectivity, portability, and power requirements for broad implementation. This paper presents the first synthesis of a novel class of two-dimensional conductive tetrapyrazinoporphyrazine-based metal–organic frameworks (MOFs) interconnected with Cu (**DC-100** and **DC-102**) and Zn ions (**DC-101**) with unprecedented chemiresistive performance toward NO detection. **DC-100** achieves an ultralow detection limit (0.47 parts-per-trillion (ppt)), rapid response (within seconds), high selectivity of NO over H_2_S, SO_2_, CO, NH_3_, and NO_2_, excellent reversibility, operation at room temperature, and low power requirements. The novel structural features and material–analyte interactions of **DC-100** with NO represent a significant conceptual advance in molecular engineering of materials for NO detection, with potential applications in environmental monitoring, industrial safety, and medical diagnostics.

## Graphical abstract

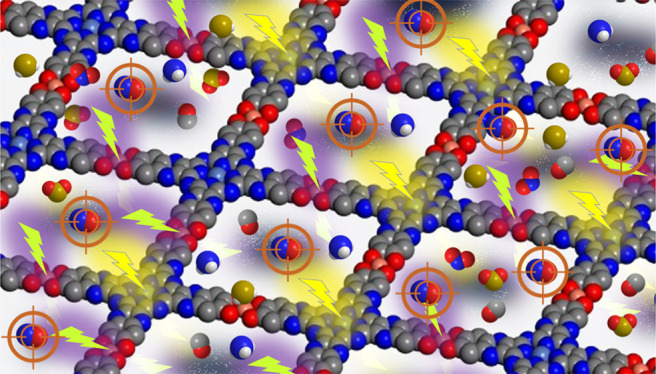

## Introduction

Molecular engineering of novel electrically conductive metal–organic framework (cMOF) materials drives progress in advancing the fields of spintronics,^1^ electrocatalysis,^2^ energy storage,^3^ and electronics^4^ as well as electronically transduced chemical sensing.^5^ Despite tremendous advances in creating highly modular chemical structures through judicious selection of metal nodes and organic linkers, strategic tuning of properties of these materials toward desired applications has remained an unresolved challenge. In part, this challenge arises due to limited chemical diversity of cMOFs, which is primarily restricted by the synthetic accessibility of organic linkers that has, with few exceptions, remained highly reliant on prototypical functionalized benzene,^6^ triphenylene,^7^ and metallophthalocyanine-based molecular building blocks.^8^ As such, enhancing the functional performance of cMOFs in devices requires fundamental advances in molecular design that enable strategic tuning of material properties through molecularly precise synthetic approaches.

Although advancing molecular engineering of cMOFs has a high potential impact in multiple aforementioned fields, this activity is particularly well suited to offer transformative function in the realm of electrically transduced chemical sensing of toxic gases, such as NO, with significant importance in environmental monitoring,^9^ industrial safety,^10^ and medical diagnostics.^11^ As a small and highly reactive gas, NO constitutes an important indicator of air quality and environmental pollution^12^ while also playing a critical role as a signaling molecule in biological systems with implications in vasodilation,^13^ neurotransmissions,^14^ and immune response.^15^ While the development of portable, selective, sensitive, reversible, and low power sensors for NO has the potential to offer transformative solutions for remediation of atmospheric pollution, ensuring personal and industrial safety, as well as noninvasive monitoring of diseases, such as asthma, cardiovascular disease, and inflammatory disorders,^16,17^ progress in this field is limited by the available NO sensing technologies that possess significant trade-offs in sensor capabilities. Traditional methods for NO detection include infrared spectroscopy,^18^ gas chromatography,^19^ and chemiluminescence.^20^ These conventional analytical techniques require bulky and costly instrumentation, high power, and trained technicians to operate, which make them unsuitable for portable NO detection. In turn, recent advancements in materials chemistry have played a significant role in enabling portable detection of NO. The use of nanomaterials, such as metal oxide semiconductors,^21^ zeolites,^22^ conjugated polymers,^23^ carbon nanotubes,^24^ graphenes,^25^ metal dichalcogenides,^26^ covalent organic frameworks,^27^ and cMOFs^28^ has achieved impressive gains in NO detection, such as high sensitivity in a portable sensing format. Despite these achievements, many of the reported sensing systems suffer from trade-offs related to synthetic accessibility, requirements for postsynthetic modification, cross-reactivity, limited chemical stability, lack of reversibility, or high-temperature operation.^29^ Moreover, from a molecular design perspective, the combined features of ultrasensitive, selective, reversible, and low power detection have proven extremely challenging to achieve,^30^ because they require access to disparate material properties of strong and selective material–analyte interactions that are prone to be highly labile to ensure reversibility. Thus, despite the significant gains in sensitive and selective detection of NO, developing fundamental molecular design criteria that can ensure ultrasensitive detection of NO with high reversibility, long-term stability, and low power detection, while minimizing interference from other gases, remains a major unresolved challenge.

This paper describes the synthesis of a novel class of two-dimensional, intrinsically cMOFs through the coordination of nickel­(II) octahydroxytetrapyrazinoporphyrazine (NiTPz-(OH)_8_) with copper ions (**DC-100**) and zinc ions (**DC-101**), as well as the coordination of the metal-free ligand octahydroxytetrapyrazinoporphyrazine (H_2_TPz-(OH)_8_) with copper ions (**DC-102**). The molecular design of these cMOFs is based on a novel metallotetrapyrazinoporphyrazine (MTPz) ligand,^31^ an analog of metallophthalocyanine (MPc), where the α-position carbon atoms are replaced with nitrogen. This substitution offers two strategic advantages for designing a highly reversible and sensitive sensor: (1) the replacement of α-position carbon atoms with electronegative nitrogen in NiTPz increases its oxidation potential by lowering the energy of its highest occupied molecular orbital (HOMO);^32^ and (2) a monomer building block that in our preliminary studies showed strong sensitivity and reversibility toward chemiresistive NO detection (Figure S26d), compared to MPc analog (Figure S26e). By leveraging the high porosity and conductivity of cMOFs, we reasoned that incorporating the NiTPz building block into a 2D cMOF would enhance NO detection by achieving fast response times, improved sensitivity, and reversibility at low driving voltages. To understand the structure–property relationships in material–analyte interactions, we systematically compared **DC-100** with control materials **DC-101** and **DC-102** to investigate how variations in the bridging ion and complex metal center influence MOF interactions with NO.

**DC-100** exhibits good conductivity (3 × 10^–^^6^ S cm^–^^1^), a large surface area (396 m^2^ g^–^^1^), low dimensionality, and ordered presentation of binding sites to the gases. This material enables four major innovations in chemical sensing: (1) ultrasensitive NO detection, with a theoretical limit of 0.47 parts-per-trillion (ppt) within 5 min of gas exposure and an initial response rate surpassing 100,000% per minute at 1 ppm of NO, both the highest among all chemiresistive NO sensors made from metal oxides,^21^ dichalcogenides,^26^ nanocomposites,^33^ and cMOFs;^34,35^ (2) short saturation time (<5 min) and a broad dynamic range at low ppb levels (10–1000 ppb), uncommon in previously reported NO gas sensors (Table S10);^34–50^ (3) exceptional selectivity, with distinct resistance changes for reducing and oxidizing gases and a 250-fold stronger response to NO over NO_2_ within 5 min at 1 ppm; and (4) unparalleled reversibility and reusability for NO detection at room temperature compared to most of the NO gas sensors (Table S10).^34–50^ These achievements showcase the tremendous power of molecular engineering through precise selection of molecular building blocks to obtain cMOFs with tailored function, which, when combined with the rapid and lower power detection, make **DC-100** a promising material for versatile applications of continuous monitoring of NO. Building on former reports on reversible NO sensing, this work introduces a conceptually novel design strategy based on a previously unreported, oxidation-resistant monomer that enables exceptional reversibility and reusability without requiring high crystallinity. The dual-active-site ligand allows tunable structure–property relationships, as demonstrated through control analogs (**DC-101** and **DC-102**). Compared with state-of-the-art materials, **DC-100** exhibits unmatched sensitivity, selectivity, and response time, establishing it as a new benchmark in chemiresistive NO sensing.

## Results and Discussion

### MOF Synthesis and Characterization

We synthesized **DC-100** using NiTPz-(OH)_8_, which is prepared in six steps from 4,5-dimethoxybenzene-1,2-diamine (Scheme S1). Optimization studies (see the Supporting Information, Section 2.2) indicated that a low concentration of NiTPz-(OH)_8_ and excess ethylenediamine (EDA), which slows down the nucleation process,^51^ were essential for crystallinity. A reaction mixture of NiTPz-(OH)_8_ (0.44 mM), Cu­(NO_3_)_2_ (2.1 equiv), and EDA (1600 equiv) in anhydrous dimethyl sulfoxide (DMSO) at 85 °C for 2 days yielded the desired crystalline product. Similarly, we synthesized **DC-101** and **DC-102** by reacting NiTPz-(OH)_8_ with Zn­(NO_3_)_2_ and H_2_TPz-(OH)_8_ with Cu­(NO_3_)_2_, respectively. The MOF preparation followed a procedure similar to that of **DC-100**, as described in the Supporting Information, Section 2.3.

Powder X-ray diffraction (PXRD) analysis suggested the 2D framework structures of **DC-100**, **DC-101**, and **DC-102** with peaks at 2θ = 3.9, 5.6, 7.8, and 27.5° (Figure 1b), matching the (100), (110), (200), and (001) facets, respectively. These results aligned with simulations based on the *P*4/*mmm* space group assuming AA-stacked NiTPz subunits. Scanning electron microscopy (SEM) revealed nanoscale cubic crystallites of **DC-100** (Figure 1d), **DC-101** (Figure 1e), and **DC-102** (Figure 1f). High-resolution transmission electron microscopy (HR-TEM) visualized 2.2–2.3 nm square pores in all three MOFs (Figure 1d–f), with corresponding fast Fourier transform (FFT) patterns shown in Figure S18g, confirming MOF formation.

**Figure 1:**
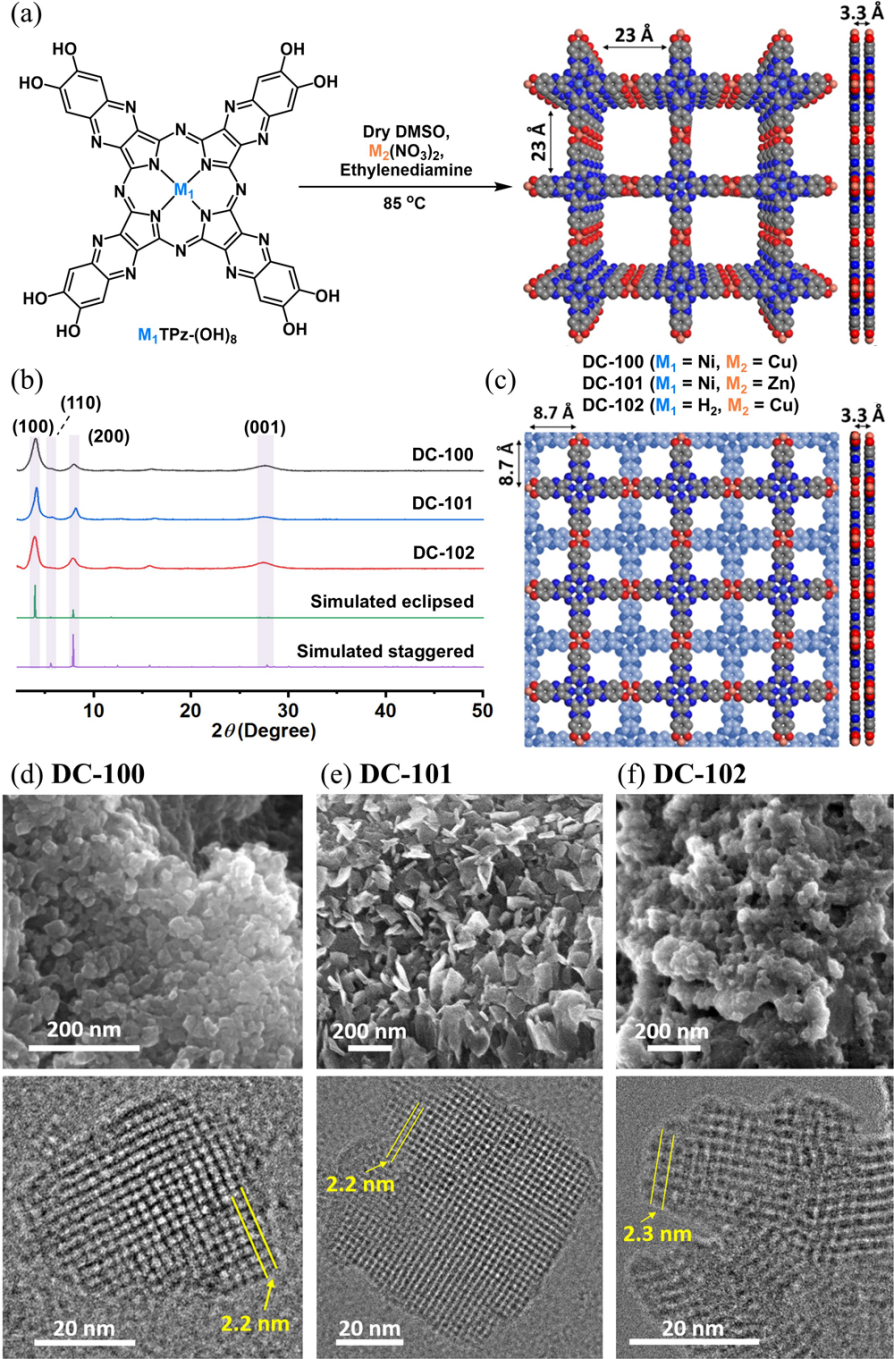
(a) Preparation of **DC-100**, **DC-101**, and **DC-102**. The product shows the simulated top and side views of eclipsed stacking MOF. (b) Experimental and simulated PXRD patterns for **DC-100**, **DC-101**, and **DC-102**. (c) Simulated top and side views of the staggered stacking MOF. SEM (top) and TEM (bottom) images for (d) **DC-100**, (e) **DC-101**, and (f) **DC-102**.

The chemical composition of **DC-100**, **DC-101**, and **DC-102** was analyzed using combustion analysis, inductively coupled plasma mass spectrometry (ICP-MS) (see the Supporting Information, Section 6), and thermogravimetric analysis (TGA) (see the Supporting Information, Section 8). The results indicated the presence of EDA and water within the crystal lattice of all of the MOFs. The presence of EDA is likely due to the chelating interaction of EDA with the bridging ions,^13^ while the water content is attributed to the hydrophilic nature of the MOF pores, which absorb moisture from the atmosphere. X-ray photoelectron spectroscopy (XPS) of **DC-100** (see the Supporting Information, Section 5) identified C, O, N, Ni, and Cu, with the Ni 2p-to-Cu 2p peak area ratio aligning with theoretical expectations (1:2). The Cu 2p_3/2_ peak revealed partial reduction of Cu­(II) (934.0 eV) to Cu­(I) (932.5 eV)^52^ in a 4:6 ratio. The presence of C–O and C=O bonds in the O 1s region (Figure S11b) supported the semiquinone structure of the MOF, and −NH_2_ groups in the N 1s region^53^ (Figure S11c) suggested the presence of EDA. The XPS spectra of **DC-101** (Figure S12) and **DC-102** (Figure S13) exhibited similar patterns in the O 1s and N 1s regions, demonstrating the chemical similarity among the three MOFs. Electron paramagnetic resonance (EPR) spectroscopy of **DC-100** and **DC-102** exhibited a broad symmetric line shape attributed to a Cu-centered radical (Figure S14a,b),^54^ while the EPR spectrum of **DC-101** closely resembled that of its monomer (Figure S14a). The charge neutrality of the material was confirmed by dye uptake experiments, where neither positively nor negatively charged dyes were absorbed by **DC-100** overnight (Figure S20).

Conductivity measurements of **DC-100**, **DC-101**, and **DC-102** at ambient conditions using a four-point probe method yielded values of 3 ± 1 × 10^–^^6^ S cm^–^^1^ (*n* = 7), 2 ± 1 × 10^–^^6^ S cm^–^^1^ (*n* = 7) and 2 ± 1 × 10^–^^7^ S cm^–^^1^ (*n* = 7), respectively. These values represent a 2 orders of magnitude improvement over their corresponding monomers: NiTPz-(OH)_8_: 5 ± 3 × 10^–^^8^ S cm^–^^1^ (*n* = 7) and H_2_TPz-(OH)_8_: 6 ± 3 × 10^–^^9^ S cm^–^^1^ (*n* = 7). Gas adsorption analysis revealed Brunauer–Emmett–Teller (BET) surface areas of 396, 305, and 408 m^2^/g for **DC-100**, **DC-101**, and **DC-102**, respectively (Figure S9a–c).

### Chemiresistive Response of **DC-100** and Its Control Materials

Each sensing experiment was performed by using at least three devices to ensure reproducibility. Additionally, both the first and second authors independently conducted sensing experiments using different batches of **DC-100** to ensure batch-to-batch consistency and minimize human error. To illustrate the chemiresistive gas sensing ability of **DC-100** (see the Supporting Information, Section 12.1, for device fabrication), we tested six analytes: NO, H_2_S, SO_2_, CO, NH_3_, and NO_2_, which are toxic pollutants or biological signaling molecules.^30,55,56^ **DC-100** can easily distinguish reducing gases and oxidizing gases, as shown in Figure 2a, with distinct positive percentage changes in the normalized sensing response (see the Supporting Information, Section 12.2, for the data processing) toward reducing gases (H_2_S, SO_2_, CO, and NH_3_) and negative percentage changes in the normalized sensing response toward oxidizing gases (NO and NO_2_). Among the tested oxidizing gases, **DC-100** exhibited a 250-fold stronger response to NO than to NO_2_ at 1 ppm over 5 min. This exceptional selectivity for NO over NO_2_, based on response magnitude, is unprecedented among the reported MOF sensors.^35,40,57^

**Figure 2:**
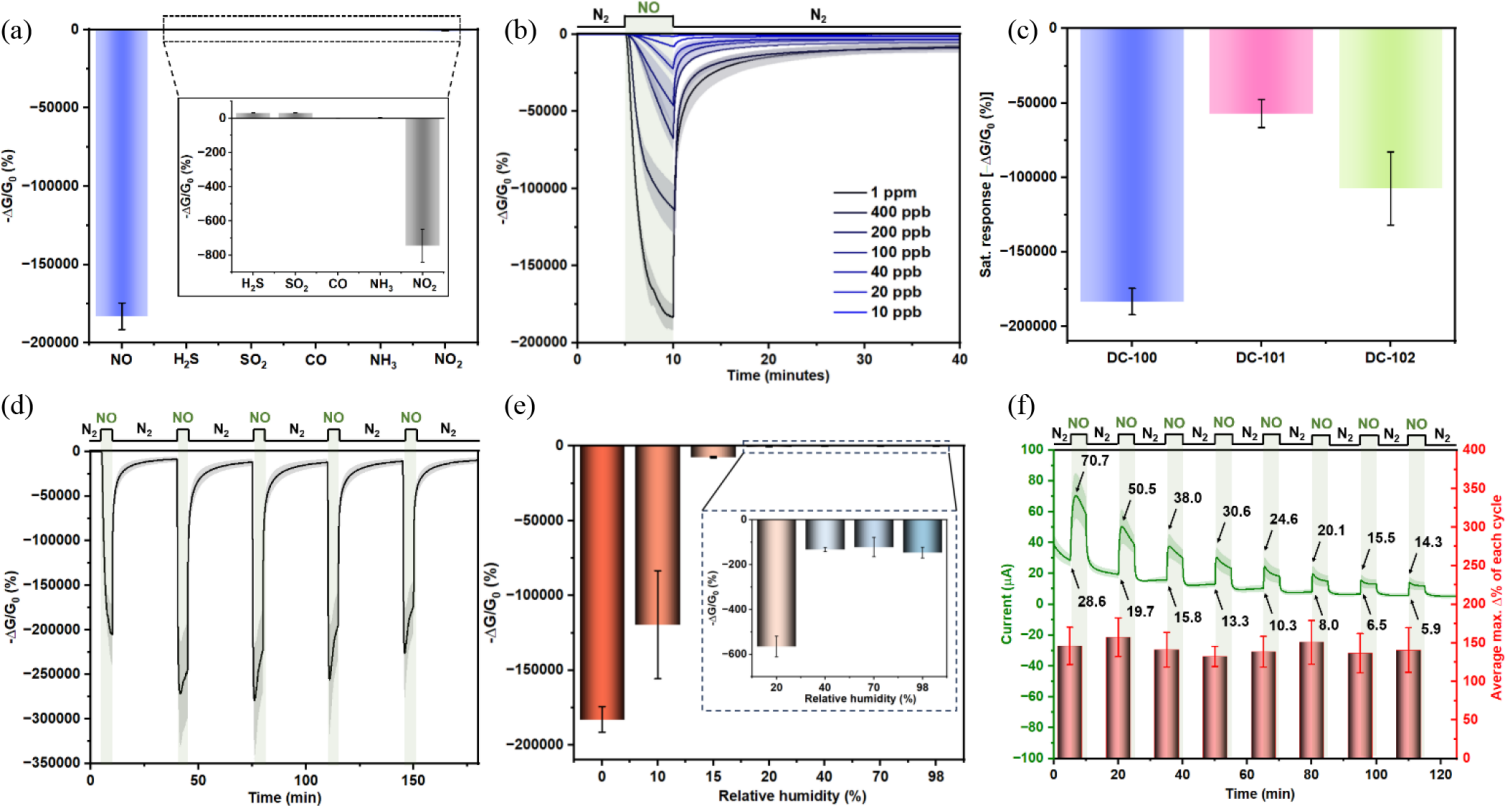
Mean normalized response (−Δ*G*/*G*_0_) of **DC-100** devices (*n* = 3) to (a) six gaseous analytes at 1 ppm over 5 min (inset: magnified view excluding NO), and (b) NO concentrations from 10 to 1000 ppb. (c) Saturated −Δ*G*/*G*_0_ of **DC-100**, **DC-101**, and **DC-102** to 1 ppm of NO in dry N_2_. −Δ*G*/*G*_0_ of **DC-100** to (d) 1 ppm of NO over five exposure–recovery cycles and (e) 1 ppm of NO under different relative humidity levels after 5 min exposure. (f) Time-dependent current (green) and average maximum % change per cycle (red bars) of **DC-100** devices during exposure–recovery cycles in 98% RH nitrogen.

As shown in Figure 2b, **DC-100** demonstrated a rapid response and excellent reversibility. The response to 1 ppm of NO reached saturation within 5 min, a significant improvement over previously reported MPc-based cMOFs (>30 min). The initial response rates of **DC-100** over the first minute of exposure at 20–1000 ppb NO ranged from 370 to 102,000% min^–^^1^, 370–2600 times faster than prior MPc-based cMOFs.^34,35^ To further investigate the kinetics between **DC-100** and NO, we plotted the initial response rate over the first minute of exposure against the NO concentration. A linear relationship (*R* ^2^ = 0.98, Figure S30a) suggests a pseudo-first-order reaction^58^ with a rate constant of 118 min^–^^1^, around 3000 times greater than other MPc-based cMOFs.^34,35^ Through a comparison of saturation time and analysis of initial response rates, **DC-100** demonstrates an exceptionally fast response to extremely low concentrations of NO. **DC-100** exhibited high sensitivity, detecting 10 ppb NO with a −1500% normalized sensing response change (Figure 2b and Figure S23g), significantly outperforming all chemiresistive NO sensors (Table S10).^34–50,59–70^ The calculated limit of detection ranged from 0.47 to 6.20 ppt for 1–5 min exposures (Table S7), among the lowest reported for NO sensors (Table S10).^34–50,59–70^ Additionally, **DC-100** showed almost full reversibility, recovering 83–96% of its response within 30 min in nitrogen (Table S8), a notable improvement over most of the NO gas sensors (Table S10).^34–50,59–70^

To investigate the role of NiTPz and Cu bridging ions on NO sensitivity, NO sensing experiments were conducted using two isoreticular MOFs, **DC-101** and **DC-102**. **DC-101** was used as a control because Zn­(II) ions cannot form stable nitrosyl complexes,^71,72^ meaning its electronic signal primarily arises from the interaction between NiTPz and NO. **DC-102** was selected as another control, as the metal-free ligand should not interact strongly with NO, meaning its electronic signal should originate from interactions between Cu bridging ions and NO. As shown in Figure 2c, MOFs with single active sites produced significantly weaker changes in normalized sensing response (**DC-100**: −184,000%, **DC-101**: −57,000%, and **DC-102**: −107,000%). The combined response of the single active site MOFs remained below that of **DC-100**, showing that embedding dual active sites within a single MOF enables a synergistic effect, significantly enhancing the NO detection sensitivity.

The reusability of **DC-100** as an NO sensor was evaluated through a 5-cycle exposure–recovery experiment (5 min exposure and 30 min recovery) (Figure 2d) and a 15-cycle test (5 min exposure and 10 min recovery) (Figure S27b). In both experiments, the initial responses in the first cycle were comparable (−205,000% in the 5-cycle test vs −201,000% in the 15-cycle test). The maximum responses were observed in the third cycle (−279,000 and −284,000%, respectively), followed by a gradual decline in subsequent cycles. Under the 5-cycle conditions, **DC-100** maintained full sensitivity (i.e., maximum −Δ*G*/*G*_0_ comparable to the first cycle) throughout all five cycles. In the 15-cycle test with a shortened recovery time, full sensitivity was retained for the first four cycles. Collectively, these results demonstrate the reproducibility and reusability of **DC-100** across different sample batches and testing conditions.

The performance of **DC-100** under humid conditions is shown in Figure 2e. The sensor response sharply decreased from −183,000 to −7400% as relative humidity (RH) increased from 0 to 15%, then stabilized in the RH range of 40–98% (−120 to −146%). Despite the significant drop, the signal-to-noise ratio of **DC-100** remains robust when compared to previously reported NO sensors such as Cu_3_(HHTP)_2_ (Figure S26f) and NiPc–CuMOF (Figure S26g), both of which exhibited negligible response to 1 ppm of NO under 98% RH in N_2_. Notably, **DC-100** exhibited reversible and consistent responses over eight exposure–recovery cycles under these humid conditions (Figure 2f and Figure S27c–e). The average responses over eight cycles remained highly reproducible: RH 40%: −121 ± 7%, RH 70%: −118 ± 7%, and RH 98%: −143 ± 8%. This observation can be interpreted based on the water adsorption isotherm (Figure S9d), which shows that pore filling begins at approximately 13% RH. Below this threshold, the MOF pores remain accessible to NO, allowing strong interactions with the active sites (bridging ions and the central TPz ion), resulting in high sensitivity. Above 13% RH, water molecules begin to block the pores and compete with NO for binding at the metal sites, sharply reducing the response. At RH above 40%, multilayer water films form on the MOF. In this regime, NO interacts weakly with the MOF surface rather than the metal nodes, resulting in lower but stable and reversible sensing responses.

In addition to humidity, oxygen is another atmospheric component that can influence the sensing performance. Response of **DC-100** in air was examined using 1 ppm of NO (Figure S23h). The maximum normalized response within 5 min in air (−176,000%) was comparable to that in dry nitrogen (−183,000%), but with a significantly faster response time in air. This result indicates that oxygen does not interfere with the NO detection. The long-term stability of **DC-100** was further evaluated using a sample stored under ambient conditions for 5 months. When tested with 1 ppm of NO in nitrogen, the aged sample showed a slightly reduced overall response (−156,000%, ∼85% of the original) but retained the same saturation time (5 min) and recovery rate (96%) as freshly prepared samples (Figure S23i). These findings confirm the excellent long-term stability and reliability of **DC-100** for practical sensing applications.

### Spectroscopic Assessment of the Interaction between NO and **DC-100** and Its Control Materials

We employed *ex situ* PXRD, EPR, and XPS spectroscopy to investigate the structural and redox stability of **DC-100** in 1 ppm of NO. After exposure to 1 ppm of NO in N_2_ for 2 h, followed by a 15 min N_2_ purge, the PXRD spectra remained almost identical to the pristine MOF (Figure 3a), confirming its structural stability. The EPR signal exhibited only a slight 4% increase (Figure 3b), indicating minimal oxidation of Cu­(I) to Cu­(II). XPS spectroscopy further confirmed the redox stability of **DC-100** at 1 ppm of NO exposure: (1) Cu 2p spectra (Figure 3c) showed only 0.2% of Cu­(I) ions oxidized to Cu­(II) ions, indicating minimal redox changes; (2) N 1s spectra (Figure 3d) showed similar peak areas for the C=N and C=N–Ni regions before and after NO exposure, supporting MOF stability. However, a small new peak (2.4%) appeared at 403.0 eV, corresponding to amine *N*-oxide,^73^ while the −NH_2_ peak area decreased. These minor changes may be attributed to ethylenediamine oxidation; (3) the O 1s spectra (Figure 3e) indicated minimal MOF oxidation, with only a slight 0.2% decrease in the C–O bond peak area. The peak area at 532.4 eV increased by 1.1%, likely due to the overlap of amine *N*-oxide binding energies^73^ with the C=O bond region. Overall, the EPR and XPS results demonstrated the high redox stability of **DC-100** after 1 ppm of NO exposure, aligning with the exceptional reversibility and reusability observed in the sensing experiments.

**Figure 3:**
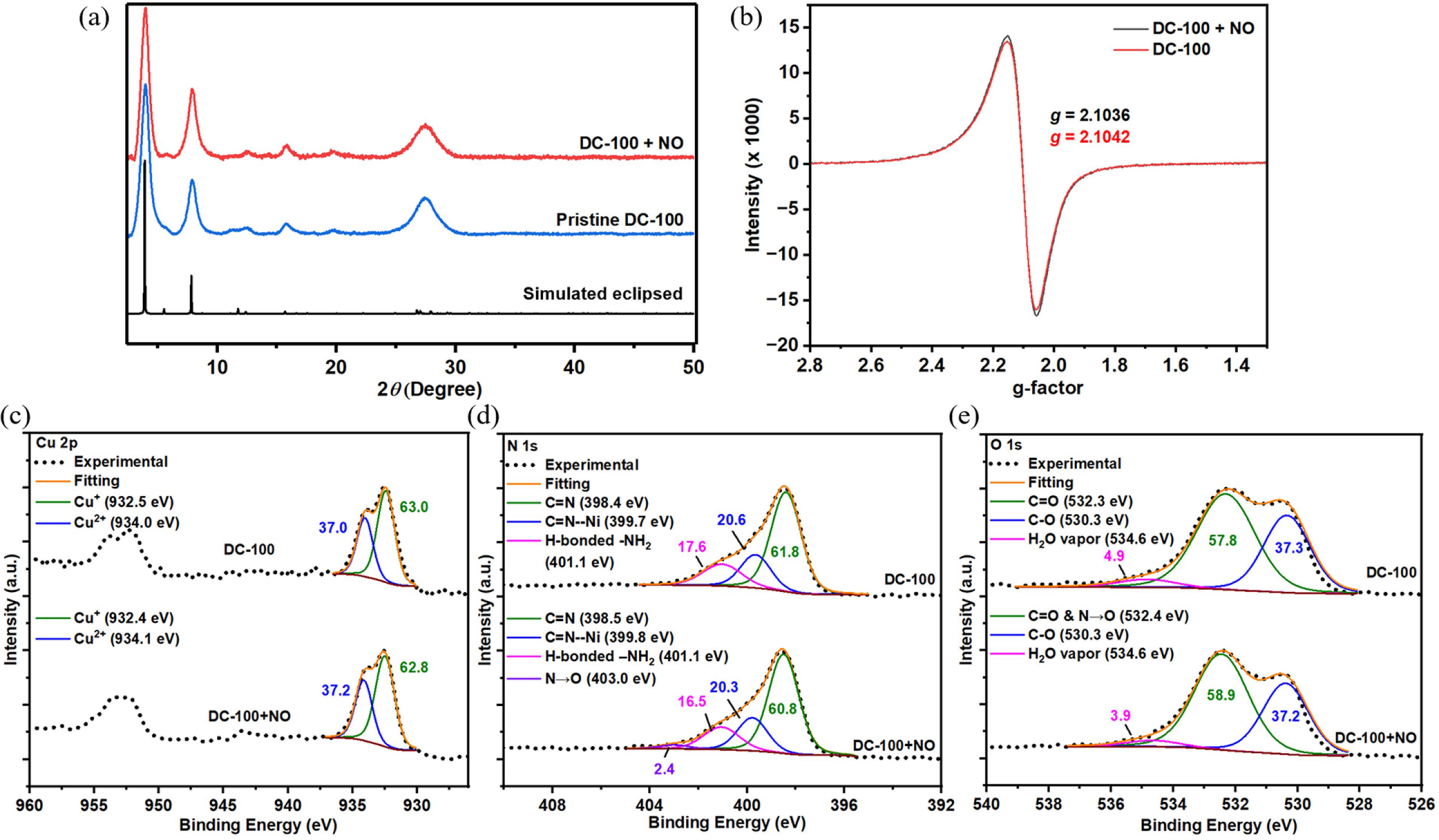
Comparison of the (a) PXRD spectra, (b) EPR spectra, (c) Cu 2p XPS spectra, (d) N 1s XPS spectra, and (e) O 1s XPS spectra of the pristine **DC-100** and **DC-100** after 2 h exposure to 1 ppm of NO.

In the diffuse reflectance infrared Fourier transform spectroscopy (DRIFTS) experiments, a 1% NO concentration was used to enhance the detection of subtle spectroscopic changes upon NO exposure. Additionally, experiments were conducted at a lower NO concentration (100 ppm) to better simulate the conditions in NO detection. As shown in Figure 4a, the spectra obtained from both experiments were largely similar, except for the absence of the NO_(g)_ band at 1900 cm^–^^1^ in the lower concentration case, likely due to weak signal intensity. The DRIFTS spectra revealed four distinct spectral regions of interest: (1) the metal–heteroatom bond stretching region (<800 cm^–^^1^), (2) the aromatic ring vibration and C–O bond stretching regions (800–1600 cm^–^^1^), (3) the N–O bond stretching region of the metal nitrosyl complex (M···N–O) and the C=O bond stretching region (1600–2000 cm^–^^1^), and (4) the broad electronic absorbance (BEA) region (>2000 cm^–^^1^).

**Figure 4:**
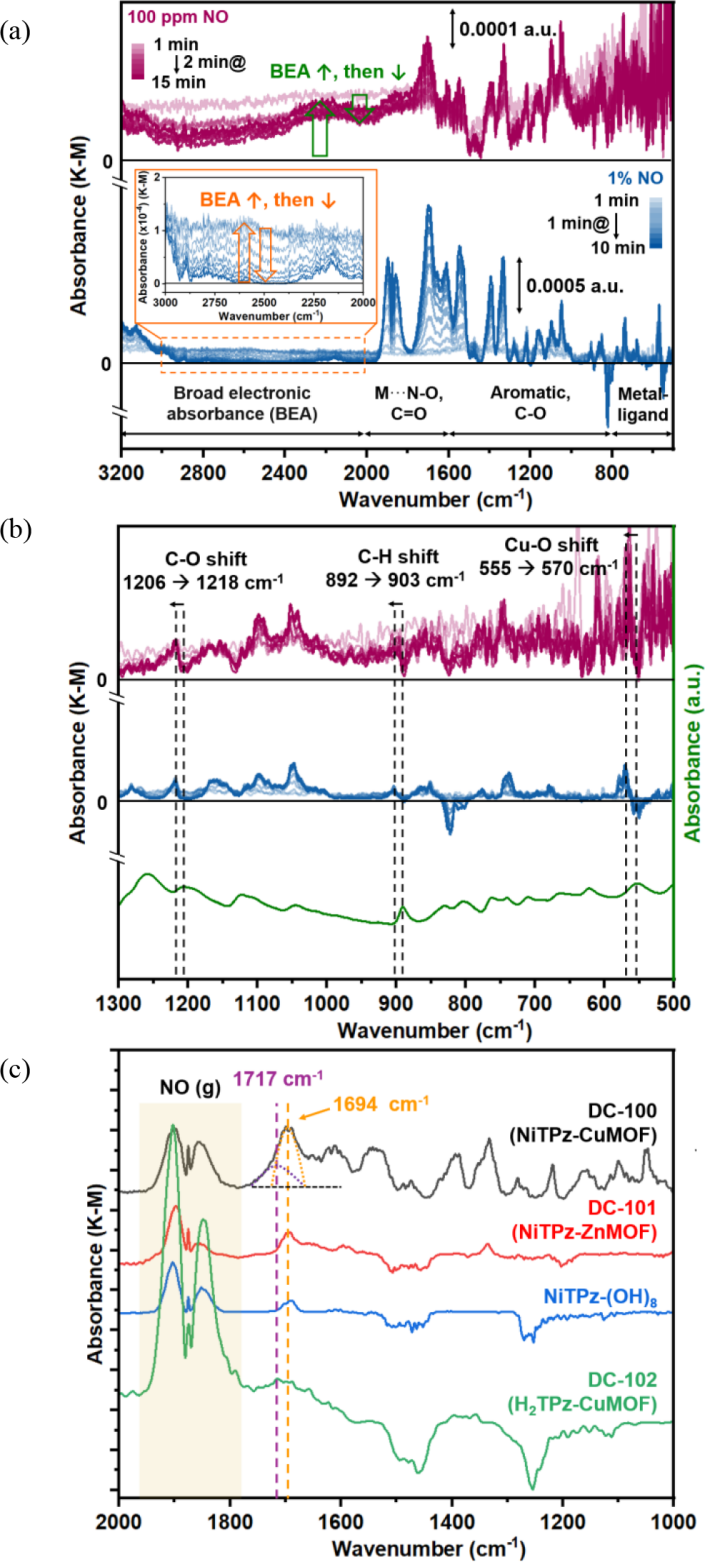
(a) DRIFTS difference spectra of **DC-100** after continuous exposure to 100 ppm of NO for 15 min (red lines) and 1% NO for 10 min (blue lines). (b) Fourier transform infrared (FT-IR) spectrum of **DC-100** (green lines), magnified DRIFTS difference spectra of the aromatic and metal–ligand regions (500–1300 cm^–1^). (c) DRIFTS difference spectra of **DC-100**, **DC-101**, NiTPz-(OH)_8_, and **DC-102** after continuous exposure to 1% NO (balance N_2_) for 10 min.

The BEA region is associated with changes in the conduction band electron population and charge-transfer reactions.^74^ Upon exposure to 1% NO, an increase in BEA intensity was observed within the first minute (Figure 4a), followed by a gradual decrease to its original absorbance level after 10 min. This pattern mirrors the response decay observed in the consecutive exposure–recovery sensing experiments (Figure 2d). During the second through fifth cycles, the normalized response decayed after reaching its peak during NO exposure. The response decay during exposure typically indicates secondary interactions between the material and NO after saturation.^43,57,75^ The combined results from DRIFTS and sensing experiments suggest that the electronic properties of **DC-100** may recover through secondary interactions with NO. A similar pattern was observed with 100 ppm of NO exposure. However, while the BEA region initially increased, it did not fully return to its original absorbance after 15 min (Figure 4a). When NO was replaced with N_2_, the BEA intensity continued to decrease over time (Figure S34d). In the aromatic and metal–ligand regions (Figure 4b), spectral shifts to higher wavenumbers were observed at 1206 cm^–^^1^ (C–O stretching),^76^ 892 cm^–^^1^ (aromatic C–H bending),^76^ and 555 cm^–^^1^ (Cu–O stretching),^77^ showing a change of the chemical environment upon NO interaction with **DC-100.**

In the M···N–O and C=O stretching regions (Figure 4c], the **DC-100** spectrum exhibited a broad absorption feature between 1666 and 1748 cm^–^^1^. To clarify the nature of this band, we examined structural analogs **DC-101**, **DC-102**, and NiTPz-(OH)_8_. A distinct peak at 1694 cm^–^^1^ was observed in the spectra of **DC-100** (NiTPz-CuMOF), **DC-101** (NiTPz-ZnMOF), and NiTPz-(OH)_8_, but it was absent in **DC-102** (H_2_TPz-CuMOF). Since the former three materials contain Ni, the peak at 1694 cm^–^^1^ is likely associated with the Ni···N–O interaction. However, in typical octahedral or square pyramidal Ni­(II) nitrosyl complexes, the N–O stretching frequency is generally observed near the NO_(g)_ frequency (1850–1920 cm^–^^1^), making it indistinguishable from free NO_(g)_.^78,79^ If the Ni­(II) ion partially distorts from the TPz ligand cavity, forming a 3-coordinated (1570–1820 cm^–^^1^)^80^ or 4-coordinated (1690–1780 cm^–^^1^)^81,82^ nitrosyl complex, it could potentially exhibit an N–O stretching signal at 1694 cm^–^^1^. Additionally, a weak band at 1717 cm^–^^1^ was detected in **DC-100** and **DC-102** but was absent in **DC-101** and NiTPz-(OH)_8_, suggesting an association with Cu···N–O interactions in Cu-containing materials **DC-100** and **DC-102**. The Cu­(II)···N–O species typically exhibit sharp peaks in the 1855–1920 cm^–^^1^ range,^83^ while Cu­(I)···N–O complexes tend to form weak peaks around 1730 cm^–^^1^.^84,85^ Based on the peak position and intensity, the 1717 cm^–^^1^ peak is more likely to correspond to a Cu­(I)···N–O interaction. These findings suggest that the broad absorption band in **DC-100** comprises two distinct and independent contributions, a sharp peak at 1694 cm^–^^1^ (purple dotted line) and a weaker peak at 1717 cm^–^^1^ (orange dotted line). We employed DFT calculations to probe potential material–analyte interactions responsible for the highly sensitive and reversible detection of NO.

### Computational Investigation of NO Binding in **DC-100** and Its Structural Analogs

To inspect the mechanism of sensing in **DC-100** and its structural analogs, density functional theory simulations using the PBEsol functional were employed to probe the thermodynamics of binding between the MOF sensors and NO. After structural optimization, NO was initialized at different sites on the framework and further relaxed; upon binding, the NO vibrational frequency was computed using the finite differences method. In **DC-100**, NO binding at both Ni and Cu was observed. The Ni···NO bond was slightly endothermic (*E*_ads_ = +360 meV) and gave an NO stretching mode of 1834 cm^–1^, close to free NO. At bridging metal sites in the framework (Cu for **DC-100** and **DC-102** and Zn for **DC-101**), NO desorbed. However, these bridging metals are all nominally in the +2 oxidation state. To model the majority population of Cu­(I) by XPS, as well as the incorporation of EDA suggested by XPS and ICP-MS, a ribbon model of **DC-100** was constructed, which contained Cu­(I) edge sites coordinated by EDA (Figure 5). At these Cu­(I) edge sites, a slightly exothermic Cu···NO bond formed (*E*_ads_ = −308 meV) with an NO stretching mode of 1685 cm^–1^, in excellent agreement with the experimental DRIFTS spectra. NO binding at these isolated Cu­(I) edge sites adds a new manifold of conduction band states associated with N–O ϖ* orbitals, but the bulk transport properties of the material remain unperturbed (Figure S38). Additional molecular models were developed to search for similar vibrational features in nonperiodic examples, but only EDA-coordinated Cu­(I) yielded the appropriate match to the experiment (see the Supporting Information, Section 15, Figure S39). This analysis suggests that NO adsorption at the EDA-coordinated edge sites contributes to the sensing performance for this group of materials.

**Figure 5:**
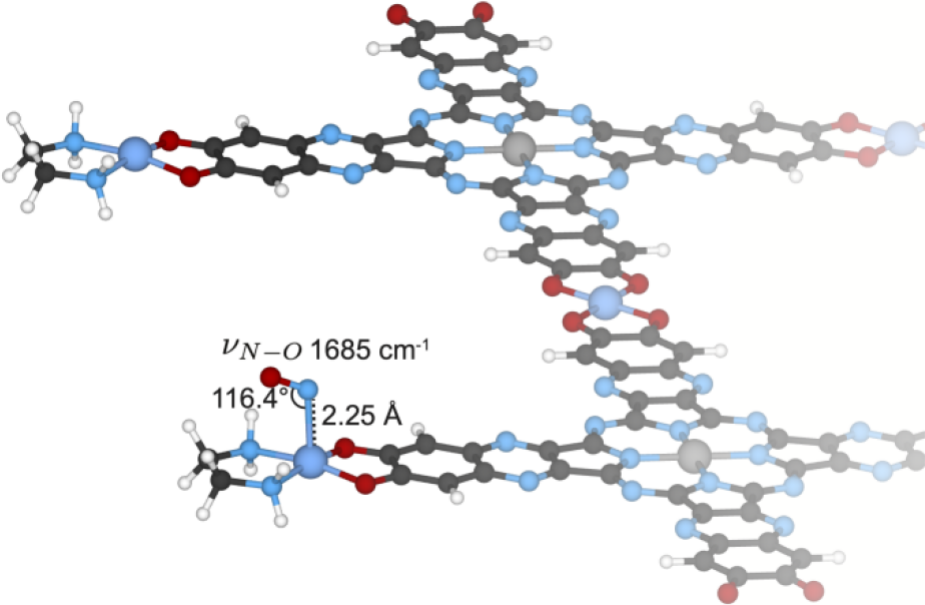
Periodic ribbon model of **DC-100**, showing Cu­(I) edge sites coordinated by EDA that bind NO in a square pyramidal configuration with the Cu–N distance labeled. The simulated NO vibrational frequency of 1685 cm^–1^ is in close agreement with the experimental DRIFTS spectrum.

## Conclusions and Outlook

In conclusion, this study presents the synthesis and characterization of **DC-100**, **DC-101**, and **DC-102**, a novel class of 2D cMOFs. Among them, **DC-100** demonstrates remarkable NO sensitivity, with a detection limit as low as 0.47 ppt within 5 min and rapid response rates exceeding 100,000% per minute at 1 ppm of NO. It shows high selectivity for NO over other reactive gases (e.g., H_2_S, SO_2_, CO, NH_3_, and NO_2_). Notably, **DC-100** offers outstanding reversibility and reusability, maintaining sensitivity across multiple cycles and stable performance in low humidity conditions, promising for real-time and continuous monitoring. Furthermore, PXRD analysis confirms the structural stability of **DC-100**, while XPS and EPR results demonstrate its redox stability, supporting its durability in NO detection. DRIFTS analysis, supported by DFT calculations, suggests that Cu­(I)···NO interactions play a crucial role in MOF···NO interactions. This work demonstrates the importance of molecular engineering of linkers in tuning structure–function relationships of MOFs. This approach provides a distinct molecular design strategy for creating materials with tailored sensing functions, merging high sensitivity and reversibility. While **DC-100** is highly promising for sensing applications, practical deployment will require addressing challenges such as synthetic complexity, potential environmental interferences, and device fragility. However, only microgram-scale quantities are needed per device, making the material feasible for integration. In addition, sensor arrays based on structurally tunable TPz-based analogs can mitigate cross-reactivity in complex gas mixtures. Together, these insights highlight the high potential utility of **DC-100** in continuous and distributed NO monitoring systems.

## Supplementary Material



## Notes

Details for synthetic conditions, PXRD test, SEM and TEM characterizations, XPS, FT-IR spectroscopy, dye adsorption experiments, BET analysis, TGA, EPR, conductivity measurement, sensing experiments, elemental analysis, DRIFTS experiments, and computational studies (PDF)
