# Supplementary material for: Molecular Engineering of a Conductive Metal–Organic Framework for Ultrasensitive, Rapid, Selective, and Reversible Sensing of Nitric Oxide
Source: J Am Chem Soc. 2025 Aug 1;147(32):29003–12. doi: 10.1021/jacs.5c07229 (PMC12356586; doi:10.1021/jacs.5c07229)
Supplement: Supplementary file 1 [file ja5c07229_si_001.pdf]

Molecular Engineering of a Conductive Metal–Organic Framework for Ultrasensitive, Rapid, Selective, and Reversible Sensing of Nitric Oxide

Joseph Y. M. Chan,[a] Elissa O. Shehayeb,[a] Doran L. Pennington,[b] Christopher H. Hendon*[b] and Katherine A. Mirica*[a]

[a] Department of Chemistry, Burke Laboratory, Dartmouth College, Hanover, New Hampshire 03755, United States

[b] Department of Chemistry and Biochemistry, University of Oregon, Eugene, Oregon 97403, United States

* Correspondence: Chendon@uoregon.edu; Katherine.A.Mirica@dartmouth.edu

Contents

**1.**	**Materials and Methods**	S3
**2.**	**Synthesis of DC-100**, **DC-101**, and **DC-102**	
	2.1 Synthesis of NiTPz-$(OH)_8$	S4
	2.2 Optimization of **DC-100**	S13
	2.3 Synthesis of **DC-101** and **DC-102**	S18
**3.**	**Brunauer–Emmett–Teller (BET) Analysis**	S20
**4.**	**Measurements of Conductivity**	S21
**5.**	**X-ray photoelectron spectroscopy (XPS)**	S22
**6.**	**Elemental Analysis**	S27
**7.**	**Electron Paramagnetic Resonance (EPR) Spectroscopy**	S29
**8.**	**Thermal Gravimetric Analyses (TGA)**	S30
**9.**	**SEM and TEM**	S31
**10.**	**ATR-FTIR spectroscopy**	S35
**11.**	**Dye uptake experiments**	S36
**12.**	**Sensing experiments**	
	12.1 Fabrication of Gas Sensors	S37
	12.2 Sensing Experiments	S38
	12.3 NO sensing performance	S42
	12.4 Other gaseous analytes sensing performance	S50
	12.5 Representative examples of NO detection by MOFs and other nanomaterials/methods	S51
**13.**	**XPS and EPR studies of NO exposure**	S53
**14.**	**Diffuse Reflectance Infrared Fourier Transform Spectroscopy (DRIFTS) of NO exposure and N_2 recovery**	S55
**15.**	**Density functional theory experiments**	S59
**16.**	**References**	S63

1. Materials and Methods

1-Hexanol and DMSO were distilled from anhydrous 4 Å molecular sieve. All other solvents and reagents were of reagent grade and used without further purification. All the reactions were performed under an atmosphere of nitrogen and were monitored by thin-layer chromatography (TLC; Merck pre-coated silica gel $60F_{254}$ plates). Chromatographic purification was performed on silica gel (Macherey-Nagel, 230–400 mesh) with the indicated eluents. 6,7-dimethoxy-1,4-dihydroquinoxaline-2,3-dione (S3)[R1] and 4,5-dimethoxybenzene-1,2-diamine[R2] were prepared as described.

^{1}H and $^{13}C\{^{1}H\}$ NMR spectra were recorded on a Bruker 600 MHz NMR spectrometer (^{1}H, 600 MHz; ^{13}C, 150.9 MHz) in $CDCl_3$, CF_3COOD or D_2SO_4. Spectra were referenced internally by using the residual solvent [^{1}H: δ = 7.26 (for $CDCl_3$), δ = 11.5 (for CF_3COOD), δ = 11.2 (for D_2SO_4)] relative to tetramethylsilane. High-resolution electrospray ionization (ESI & MALDI) mass spectrum was recorded on a Waters Q-TOF Ultima ESI. Powder X-ray diffraction (PXRD) measurements were performed with a Rigaku sixth generation MiniFlex X-ray diffractometer with a 600 W (40 kV, 15 mA) CuKα (α = 1.54 Å) radiation source.

2. Synthesis of DC-100

2.1 Synthesis of MTPz-(OH)$_8$

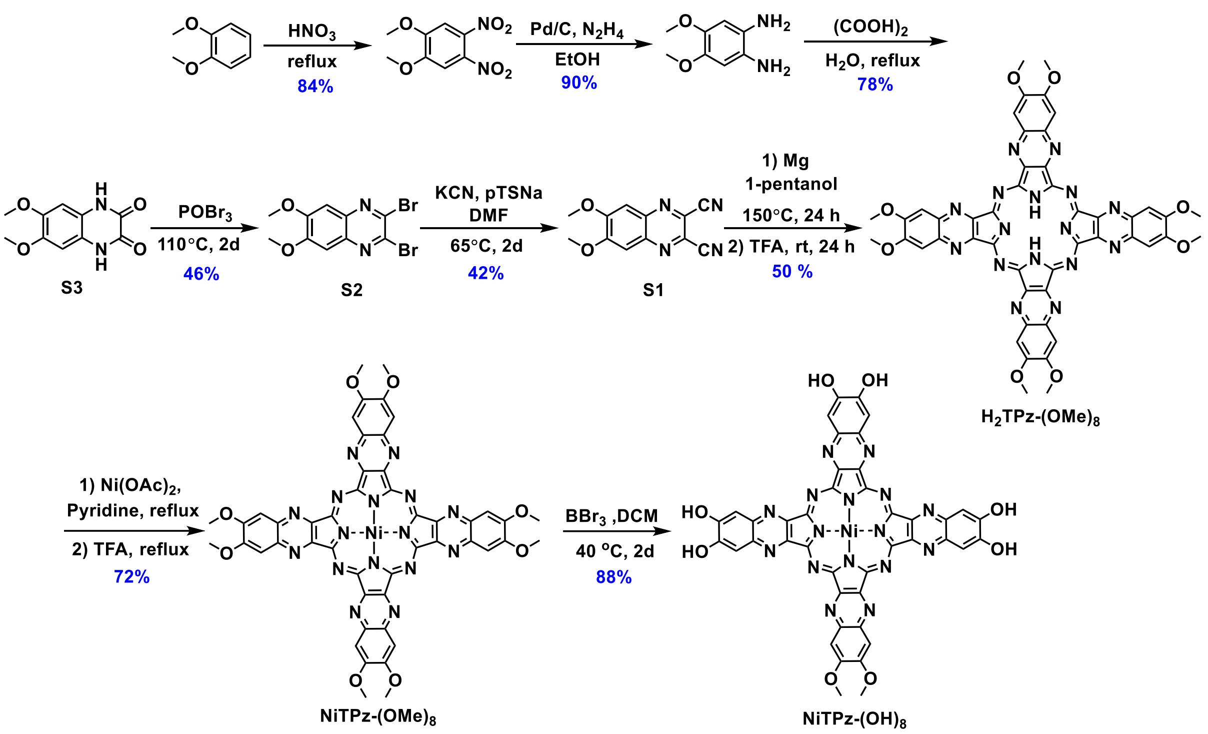

Scheme S1. Preparation of NiTPz-(OH)$_8$.

Preparation of S2

A round-bottom flask was charged with **S3** (4 g, 18 mmol),[R1] phosphoryl bromide (12 g, 42 mmol), and 40 mL of dry toluene. The reaction mixture was heated at 110 °C for 2 days until a brown suspension was observed. The reaction mixture was then cooled to room temperature and carefully poured over a beaker containing excess ice. The product was extracted with DCM (25 mL) three times. The organic layer was collected and evaporated to yield a yellow solid (2.88 g, 46%). ^{1}H NMR (CDCl$_3$, 600 MHz): 7.28 (s, 2H) and 4.04 (s, 6H). ^{13}C{^{1}H} NMR (CDCl$_3$, 150.9 MHz): 153.75, 142.72, 137.97, 105.74, 56.55. HRMS (ESI): calcd for $C_{10}H_9Br_2N_2O_2$ $[M+H]^+$ 346.9031, found 346.9032.

Preparation of S1

S2 (400 mg, 1.15 mmol), potassium cyanide (165 mg, 2.53 mmol), and p-toluene sulfinic acid sodium salt (225 mg, 1.27 mmol) were added to a Schlenk tube with a stir bar and degassed by three vacuum-nitrogen cycles. Under nitrogen, 2 mL of anhydrous DMF was added via syringe. The reaction mixture was heated at 65 °C under nitrogen for 48 hours. The mixture was then diluted with a large amount of water to precipitate the product, which was collected by suction filtration and washed thoroughly with water. After drying, the solid was soaked and washed extensively with dichloromethane. The organic solution was evaporated to obtain the crude product. Column chromatography over a silica bed using a DCM/hexane mixture (3:2, v/v) afforded the pure product as a yellow powder (116 mg, 42%). ^{1}H-NMR ($CDCl_3$, 600 MHz): 7.41 (s, 2H) and 4.13 (s, 2H). ^{13}C{^{1}H} NMR ($CDCl_3$, 150.9 MHz): 157.18, 140.48, 128.23, 114.02, 106.36, 57.16. HRMS (ESI): calcd for $C_{12}H_9N_4O_2$ $[M+H]^+$ 241.0726, found 241.0735.

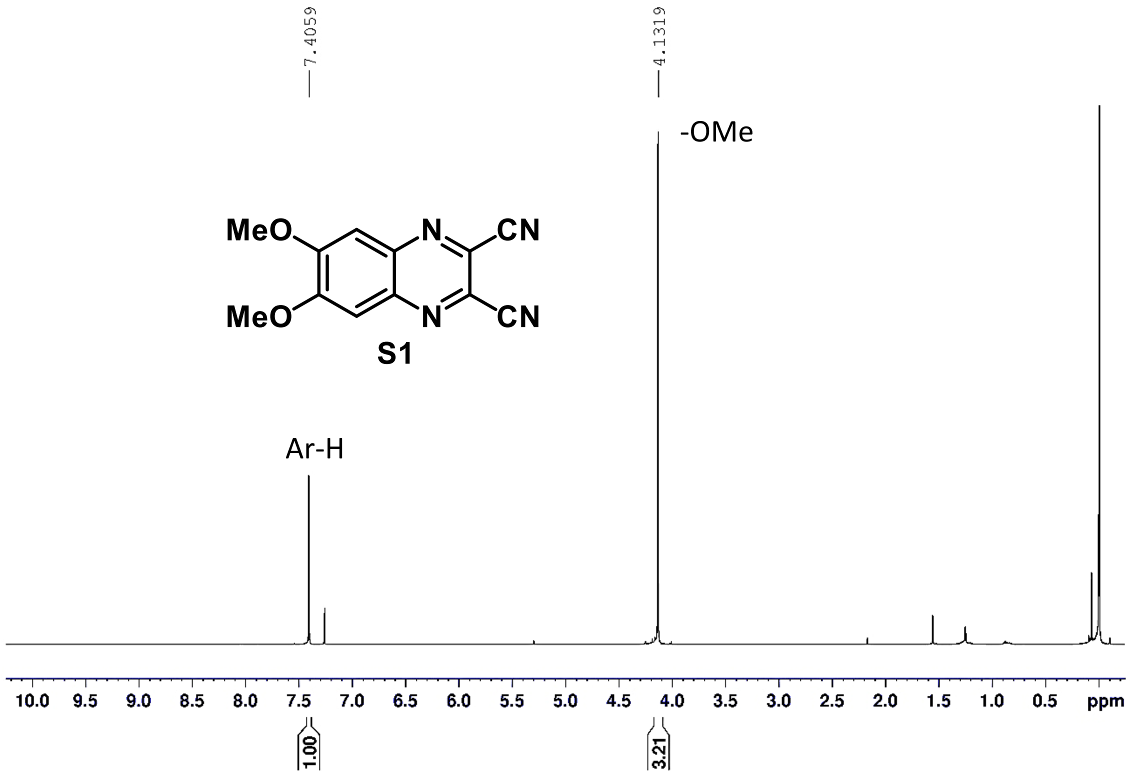

Figure S1. ^{1}H NMR spectrum of **S1** in $CDCl_3$.

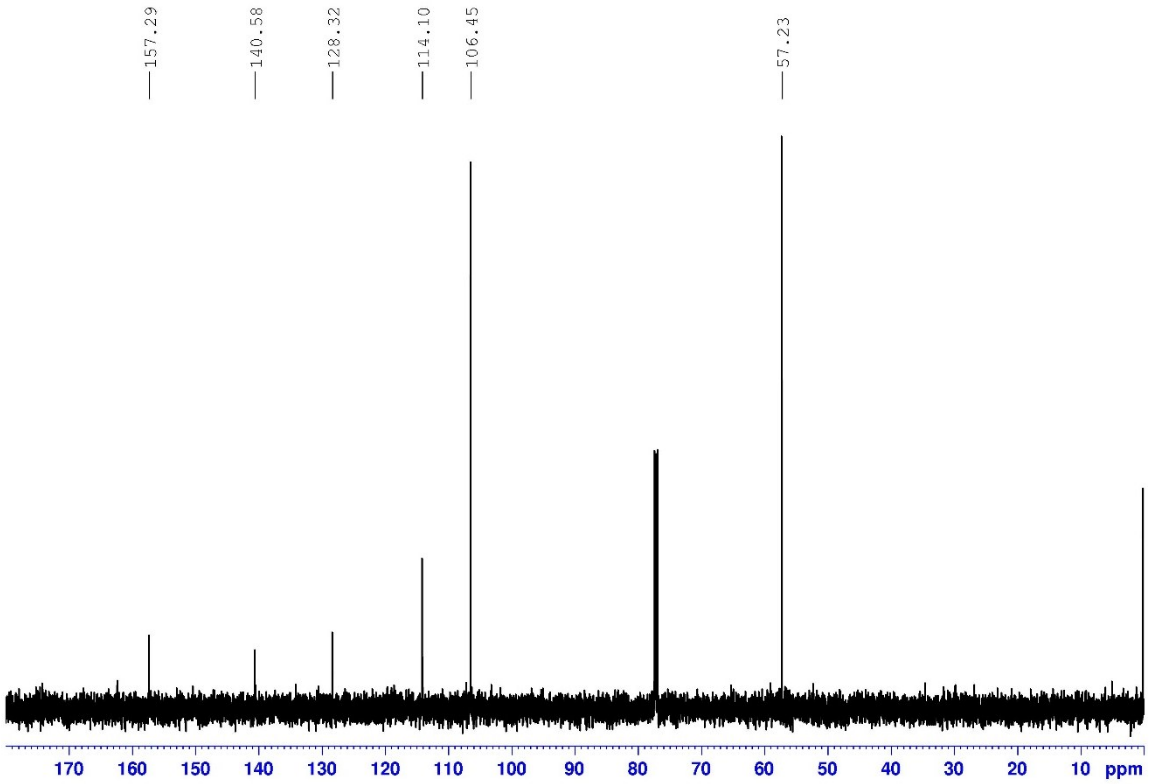

Figure S2. $^{13}C\{^{1}H\}$ NMR spectrum of **S1** in $CDCl_3$.

Preparation of H_2TPz-$(OMe)_8$

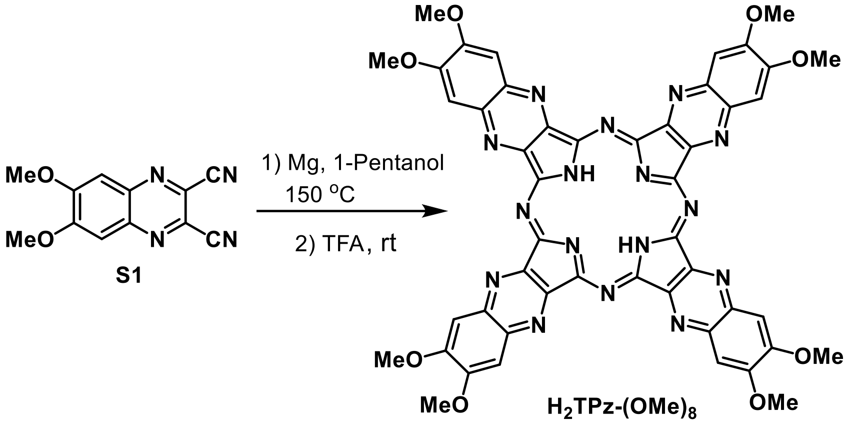

A mixture of **S1** (200 mg, 0.83 mmol) and magnesium (82 mg, 3.37 mmol) in 1-pentanol (5 mL) was heated at 150 °C overnight. The mixture was allowed to cool, and hexane (20 mL) was added to precipitate the crude product, which was separated by suction filtration. The solid was collected and subsequently washed and decanted using the following solutions in a centrifuge tube: i) methanol, ii) methanol/acetic acid (4:1, v/v), iii) acetone, iv) acetone/acetic acid (4:1, v/v), and v) acetone. The remaining precipitate was dried in a vacuum and stirred in TFA overnight to yield the pure product (101 mg, 49%). ^1H NMR (CF_3COOD, 600 MHz): δ 8.51 (s, 8 H, Ar-H), 4.57 (s, 24 H, $-OCH_3$), -0.01 (br, 2 H, -NH). ^{13}C NMR spectrum was not obtained due to the poor solubility in all common solvents. HRMS (MALDI): calcd for $C_{48}H_{35}N_{16}O_8$ $[M+H]^+$ 963.2818, found 963.2839; calcd for $C_{48}H_{34}N_{16}O_8Na$ $[M+Na]^+$ 985.2638, found 985.2671.

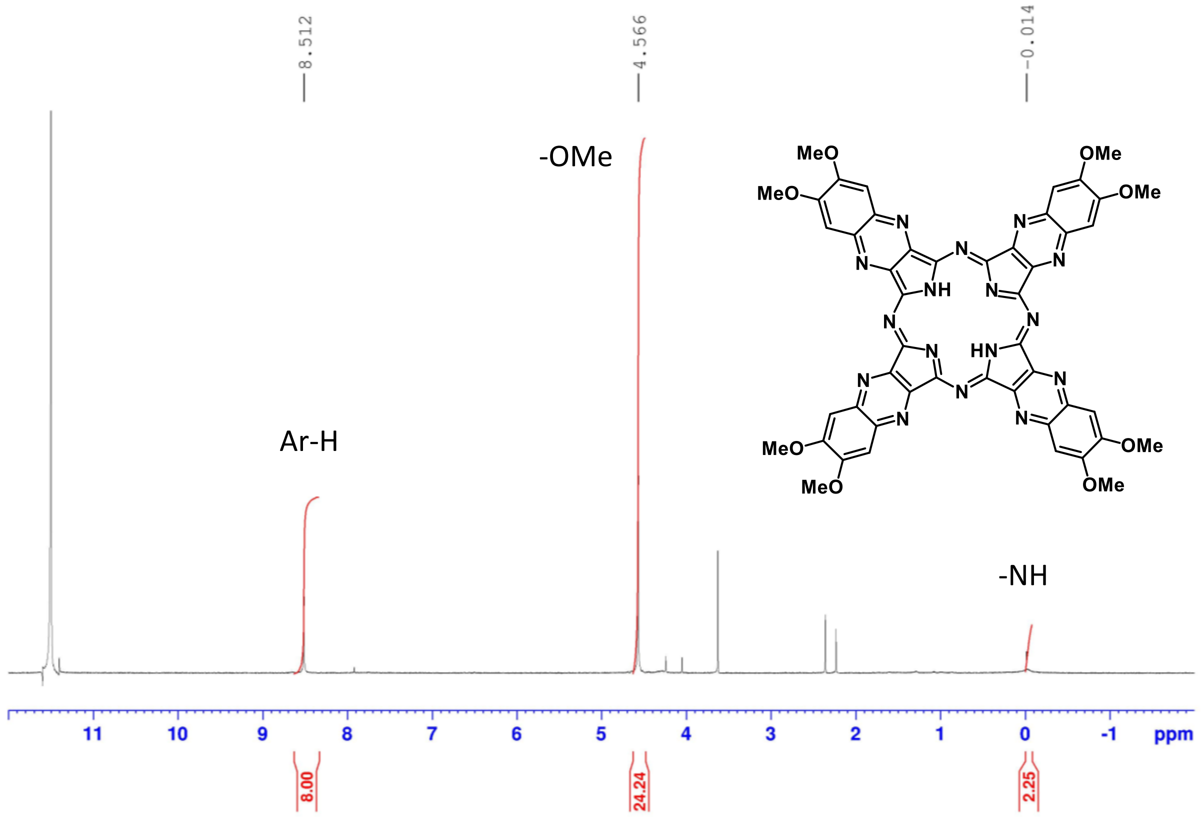

Figure S3. ^{1}H NMR spectrum of **H_2TPz-(OMe)$_8$** in CF_3COOD.

Preparation of NiTPz-(OMe)$_8$

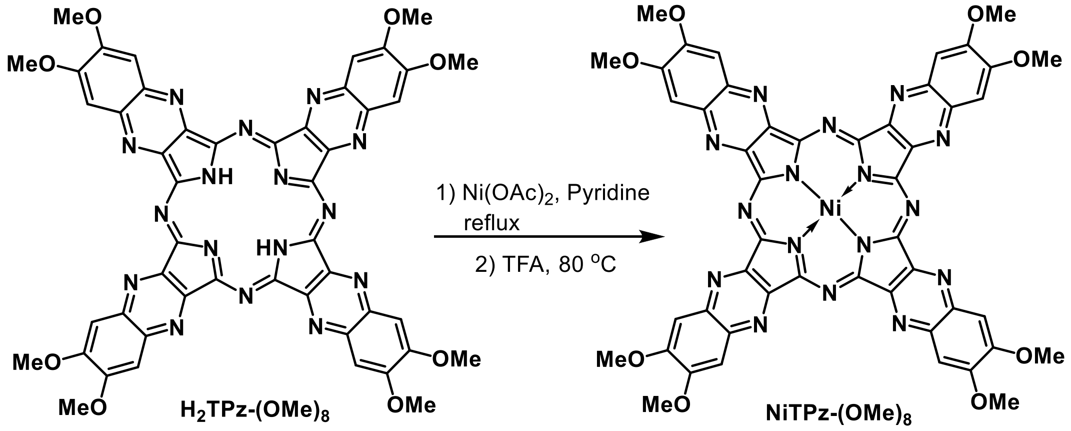

150 mg of H_2TPz-(OMe)$_8$ and nickel(II) acetate tetrahydrate (149 mg, 0.60 mmol) were dissolved in pyridine (10 mL) and heated under reflux for 1 hour. The solvent was removed under vacuum. The remaining solid was heated in TFA (10 mL) at 80 °C overnight. The solvent was removed under vacuum. The remaining solid was was washed with 25 mL of i) water, ii)

DMF/acetone (1:4, v/v), and iii) acetone in a centrifuge tube. The precipitate was dried in a vacuum oven to yield the pure dark green product (114.0 mg, 72%).^{1}H NMR (CF_3COOD, 600 MHz): δ 8.46 (s, 8 H, Ar-H), 4.55 (s, 24 H, -OCH_3). ^{13}C NMR spectrum was not obtained due to the poor solubility in all common solvents. HRMS (MALDI): calcd for $C_{48}H_{32}N_{16}O_8Ni$ $[M]^+$ 1018.1937, found 1018.1917.

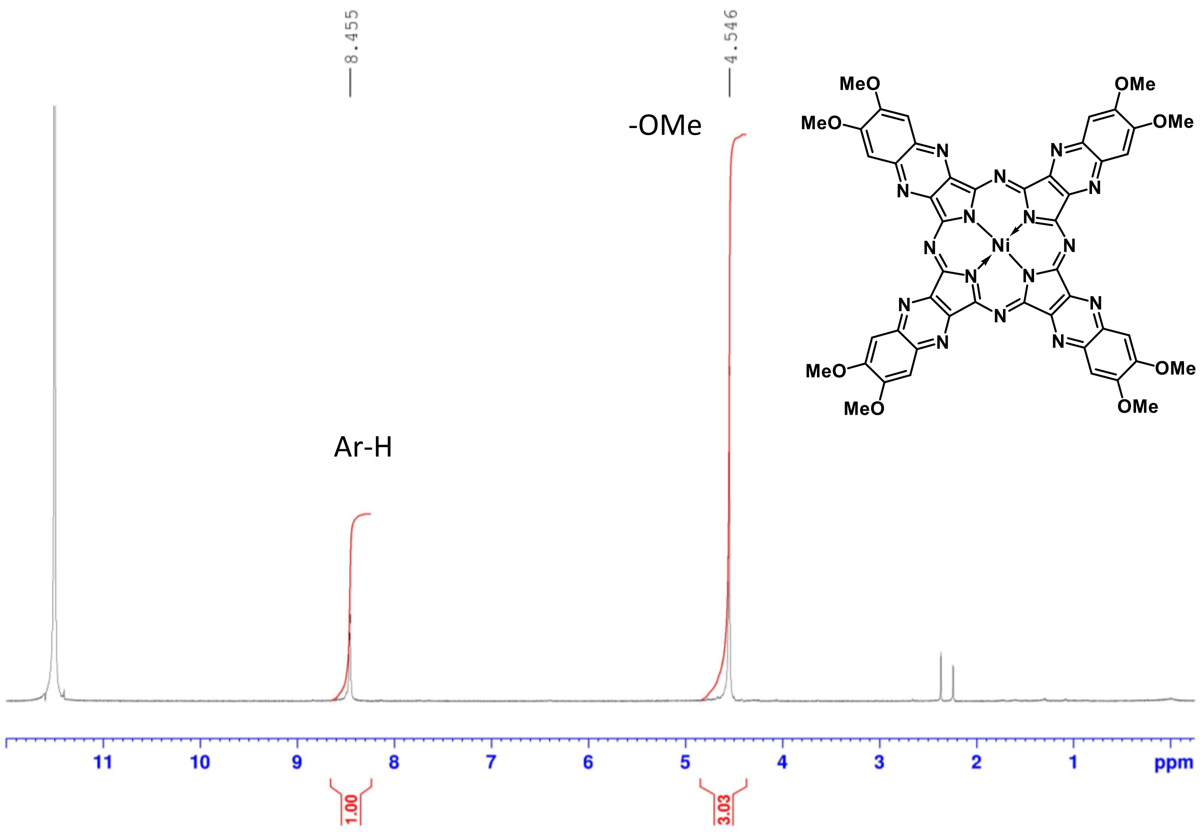

Figure S4. ^{1}H NMR spectrum of **NiTPz-(OMe)$_8$** in CF_3COOD.

Preparation of NiTPz-(OH)$_8$

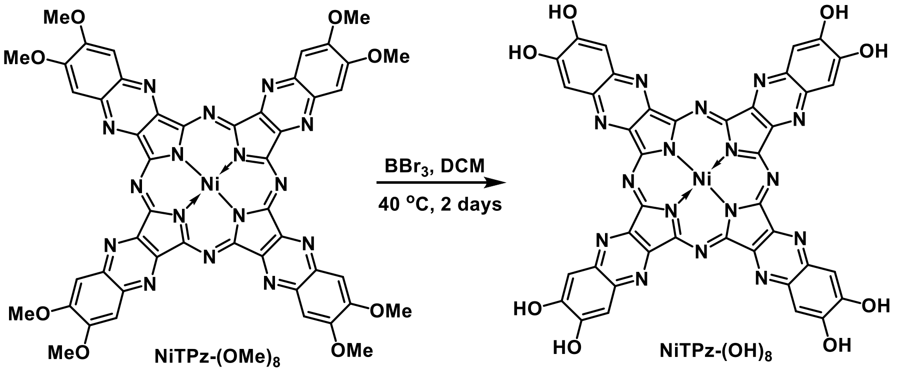

NiTPz-(OMe)$_8$ (100 mg, 98.1 μmol) was suspended in dry dichloromethane (6.5 mL), and boron tribromide (0.75 mL, 7.85 mmol) was added under nitrogen. The mixture was heated at 40 °C for 2 days. The reaction mixture was quenched with a mixture of ice and methanol (30 mL), and the resulting suspension was centrifuged and decanted. The obtained solid was then washed, centrifuged, and decanted successively with 25 mL of i) methanol/acetic acid mixture (4:1, v/v), ii) DMF, and iii) acetone. The precipitate was dried in a vacuum oven to yield the pure dark green product (75.2 mg, 88%). ^1H NMR (D_2SO_4, 600 MHz): δ 9.32 (s, 8 H, Ar-H). ^{13}C NMR spectrum was not obtained due to the poor solubility in all common solvents. HRMS (MALDI): calcd for $C_{40}H_{16}N_{16}O_8Ni$ $[M]^+$ 906.0685, found 906.0699.

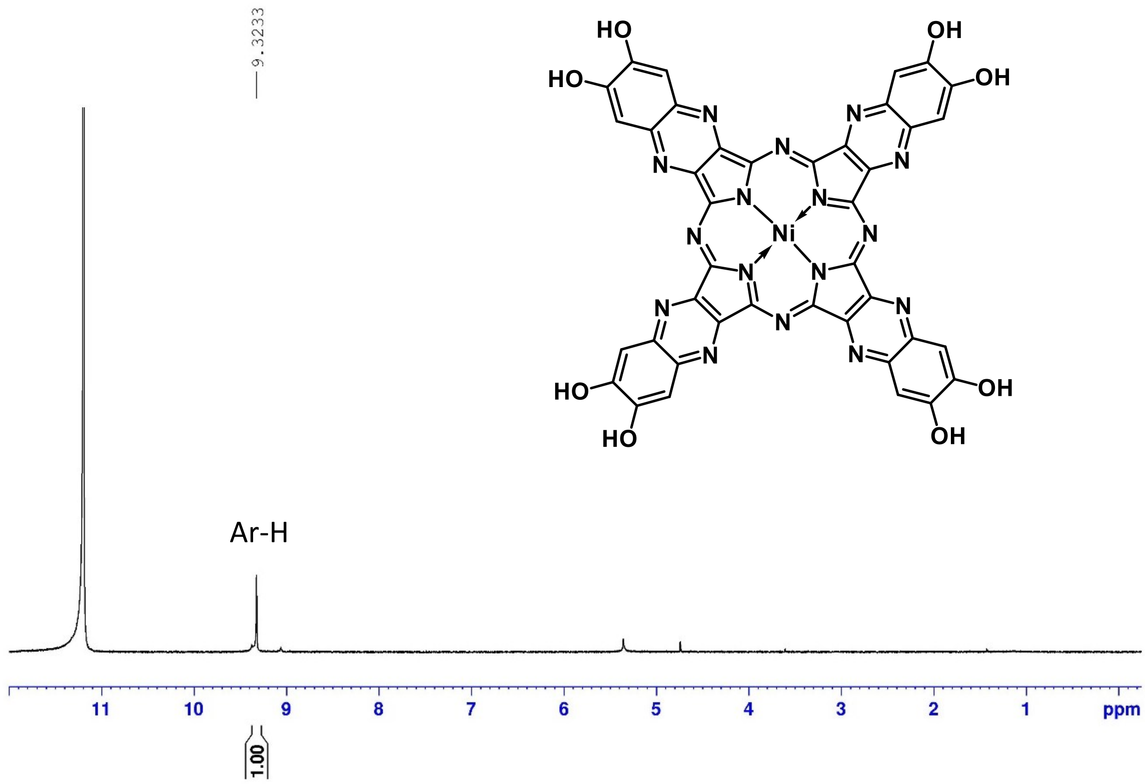

Figure S5. ^{1}H NMR spectrum of **NiTPz-(OH)$_8$** in D_2SO_4.

H_2TPz-(OMe)$_8$ (94.5 mg, 98.1 μmol) was suspended in dry dichloromethane (6.5 mL), and boron tribromide (0.75 mL, 7.85 mmol) was added under nitrogen. The mixture was heated at 40 °C for 2 days. The reaction mixture was quenched with a mixture of ice and methanol (30 mL), and the resulting suspension was centrifuged and decanted. The obtained solid was then washed, centrifuged, and decanted successively with 25 mL of i) methanol/acetic acid mixture (4:1, v/v), ii) DMF, and iii) acetone. The precipitate was dried in a vacuum oven to yield the pure dark green product (54.2 mg, 65%). ^{1}H NMR (D_2SO_4, 600 MHz): δ 9.04 (s, 8 H, Ar-H), 3.25 (s, 8 H, O-H), 0.81 (s, 2 H, N-H). ^{13}C NMR spectrum was not obtained due to the poor solubility in all common solvents. HRMS (MALDI): calcd for $C_{40}H_{19}N_{16}O_8$ $[M+H]^+$ 851.1572, found 851.1566.

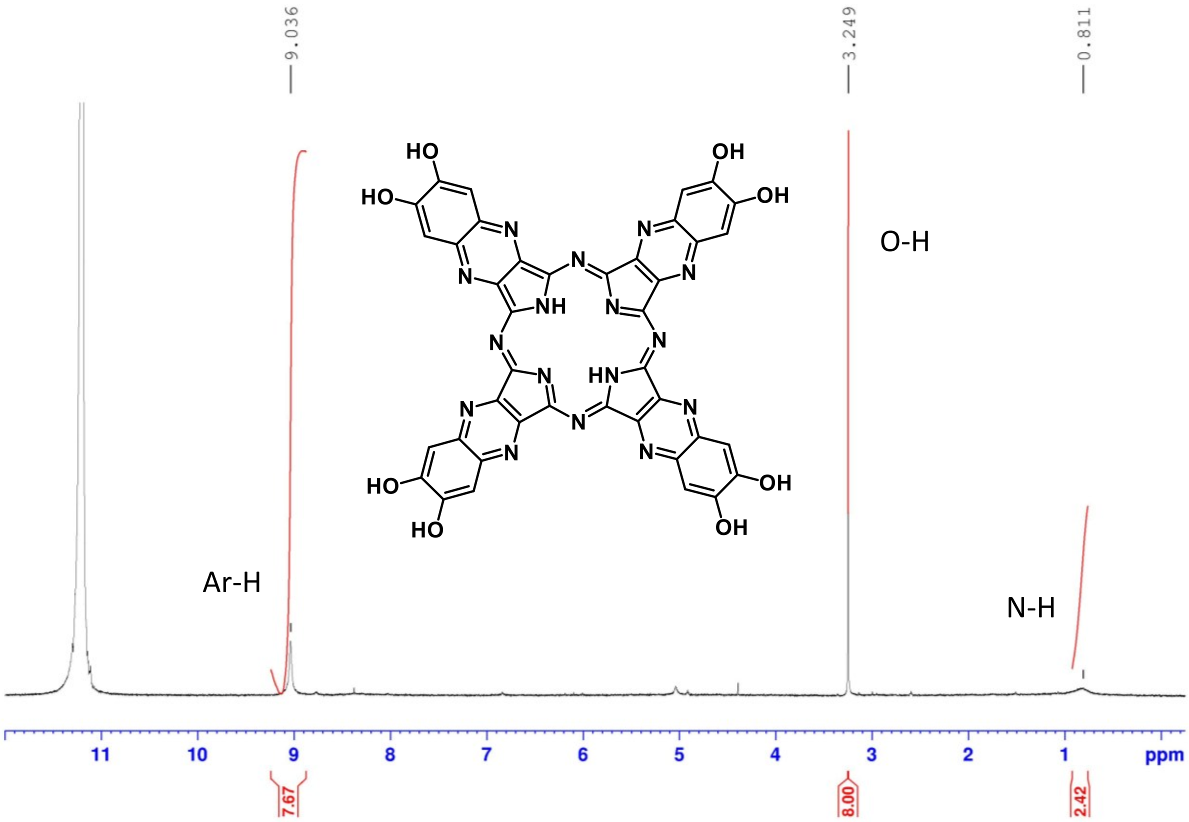

Figure S6. ^{1}H NMR spectrum of **H_2TPz-(OH)$_8$** in D_2SO_4.

2.2 Optimization of DC-100

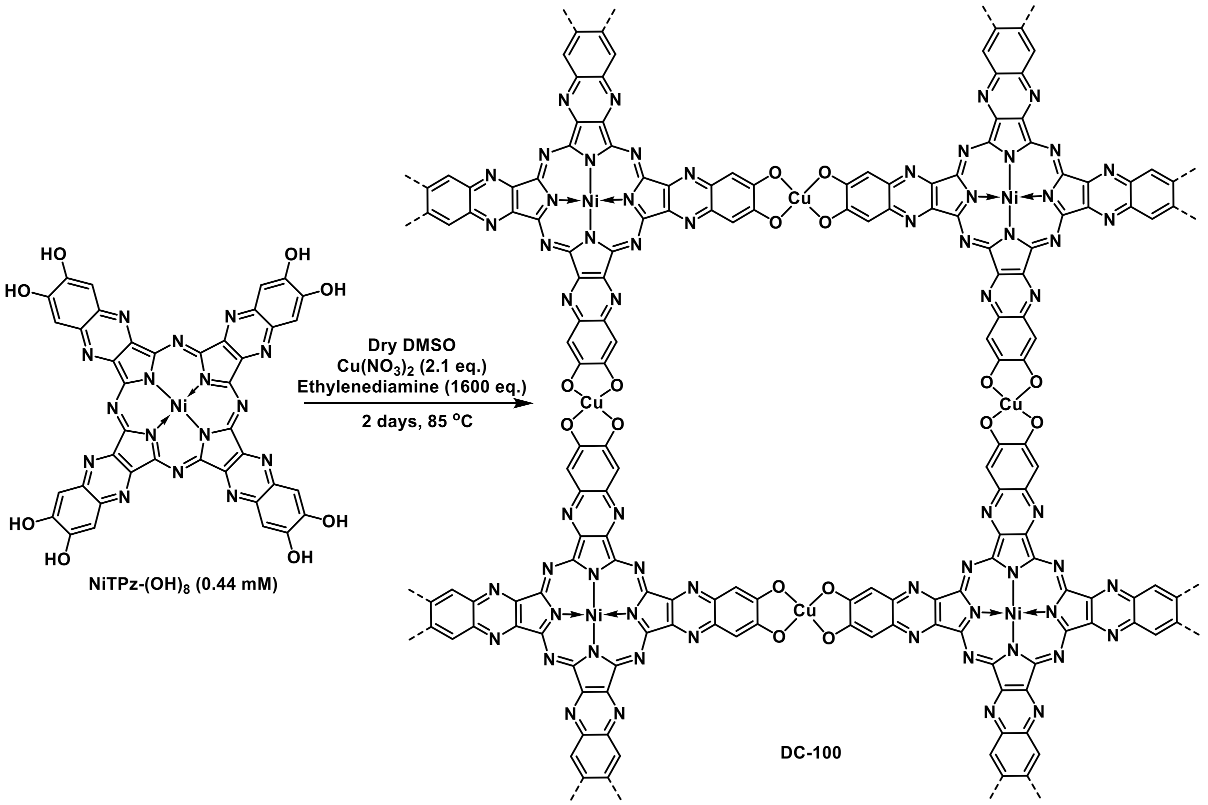

To optimize the crystallinity and yield of the synthesized MOF, we utilized NiTPz-(OH)$_8$ as a model compound and investigated various reaction parameters. We systematically altered the solvents, reaction temperature, reaction duration, the source of copper(II) salts, and concentrations of NiTPz-(OH)$_8$. Our studies revealed that high dilution and the addition of excess ethylenediamine (EDA) were crucial for synthesizing crystalline MOFs. We conducted the MOF synthesis at three different concentrations (5.79 mM, 1.93 mM, and 0.44 mM) of NiTPz-(OH)$_8$ in DMSO. The PXRD spectra of MOF samples isolated from conditions at 5.79 mM and 1.93 mM ligand concentrations exhibited similar patterns (Table S1, entries 6-8), while the MOF prepared at 0.44 mM ligand concentration showed a stronger (100) peak, although the (110) peak was still not evident in the PXRD spectrum (Table S1, entry 8). Ensuring complete dissolution of NiTPz-(OH)$_8$ through a high dilution strategy was key for optimizing MOF crystallinity. To enhance the crystallinity of the MOF further, we utilized EDA as a

mediator, which can bind with Cu(II) ions to slow down MOF precipitation.[R34] The addition of 20 equivalents of EDA notably decelerated the MOF precipitation process, leading to the presence of sharp (100) and (200) diffraction peaks in the PXRD spectrum (Table S1, entry 10). Increasing the amount of EDA to 1600 equivalents effectively prevented MOF precipitation at room temperature, allowing the MOF crystallites to maximize growth before crystallizing out from the reaction mixture. The resulting MOF displayed sharp (100) and (200) peaks, and a small (110) peak eventually emerged in the PXRD spectrum (Table S1, entry 13), indicating a significant increase in crystallite size. After optimization, we determined that a reaction mixture comprising NiTPz-$(OH)_8$ (0.44 mM), $Cu(NO_3)_2$ (2.1 equivalents), and EDA (1600 equivalents) in anhydrous DMSO at a temperature of 85°C for a duration of 2 days yielded the desired product with good crystallinity.

Table S1. The optimization of synthetic conditions for **DC-100**.

Entry	Solvent	[NiTPz-(OH)$_8$] / mM	Cu(II) salt	Base	Temperature / ^oC
1	DMSO	5.79	$Cu(OAc)_2$	-	155
2	DMSO	5.79	$Cu(OAc)_2$	-	105
3	DMSO	5.79	$Cu(OAc)_2$	-	85
4	Formamide	5.79	$Cu(OAc)_2$	-	85
5	Ethylene glycerol	5.79	$Cu(OAc)_2$	-	85
6	DMSO	5.79	$Cu(C_5H_4F_3O_2)_2$	-	85
7	DMSO	1.93	$Cu(OAc)_2$	-	85
8	DMSO	0.44	$Cu(OAc)_2$	-	85
9	DMSO	0.44	$Cu(NO_3)_2$	NH_3(aq) (20 eq.)	85
10	DMSO	0.44	$Cu(NO_3)_2$	Ethylenediamine (20 eq.)	85
11	DMSO	0.44	$Cu(NO_3)_2$	Ethylenediamine (40 eq.)	85
12	DMSO	0.44	$Cu(NO_3)_2$	Ethylenediamine (60 eq.)	85
**13**	**DMSO**	**0.44**	**$Cu(NO_3)_2$**	**Ethylenediamine (1600 eq.)**	**85**

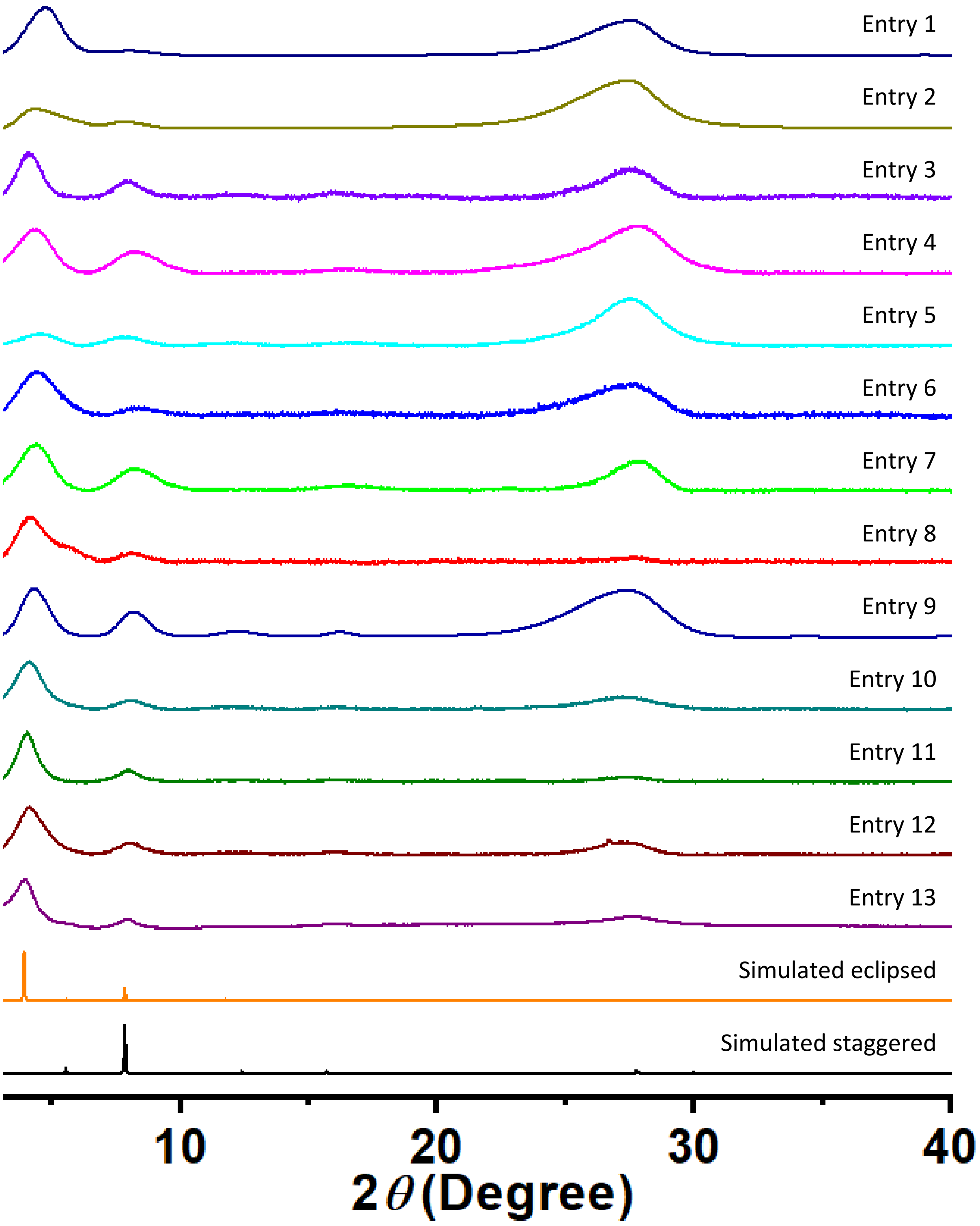

Figure S7. PXRD spectra of the materials obtained under synthetic conditions described in **Table S1**.

Synthesis of DC-100 under optimized conditions (Entry 13)

In a 100 mL Schlenk flask, 10 mg of NiTPz-$(OH)_8$ were dissolved in 25 mL of anhydrous DMSO. The mixture was sonicated for 15 minutes until the NiTPz-$(OH)_8$ was completely dissolved. 1.118 mL of ethylenediamine (1600 eq.) was added to the solution and the mixture was shaken well. 5.4 mg (2.1 eq.) of $Cu(NO_3)_2{\cdot}2.5H_2O$ was added to the resulting solution. The solution was then heated in an oven at 85 °C for 2 days. After cooling to room temperature, the black solid was collected by centrifugation, washed with methanol (20 mL × 2), deionized water (20 mL × 2), and acetone (20 mL × 3) using a vortex, and transferred to a vacuum (20 mTorr) oven at 65 °C. The solid was then dried for 24 hours to obtain **DC-100**, a dark black powder. (Yield: 78%)

2.3 Synthesis of DC-101 and DC-102

Synthesis of DC-101

In a 25 mL Schlenk flask, 10 mg of NiTPz-$(OH)_8$ were dissolved in 5 mL of anhydrous DMSO. The mixture was sonicated for 15 minutes until the NiTPz-$(OH)_8$ was completely dissolved. 29.5 µL of ethylenediamine (40 eq.) was added to the solution and the mixture was shaken well. 6.9 mg (2.1 eq.) of $Zn(NO_3)_2{\cdot}6H_2O$ was added to the resulting solution. The solution was then heated in an oven at 85 °C for 18 hours. After cooling to room temperature, the black solid was collected by centrifugation, washed with methanol (20 mL × 2), deionized water (20 mL × 2), and acetone (20 mL × 3) using a vortex, and transferred to a vacuum (20 mTorr) oven at 65 °C. The solid was then dried for 24 hours to obtain **DC-101**, a dark black powder. (Yield: 89%)

Synthesis of DC-102

In a 100 mL Schlenk flask, 10 mg of H_2TPz-$(OH)_8$ were dissolved in 25 mL of anhydrous DMSO. The mixture was sonicated for 15 minutes until the H_2TPz-$(OH)_8$ was completely dissolved. 2.236 mL of ethylenediamine (3200 eq.) was added to the solution and the mixture was shaken well. 4.8 mg (2 eq.) of $Cu(NO_3)_2{\cdot}2.5H_2O$ was added to the resulting solution. The solution was then heated in an oven at 85 °C for 2 days. After cooling to room temperature, the black solid was collected by centrifugation, washed with methanol (20 mL × 2), deionized water (20 mL × 2), and acetone (20 mL × 3) using a vortex, and transferred to a vacuum (20 mTorr) oven at 65 °C. The solid was then dried for 24 hours to obtain **DC-102**, a dark black powder. (Yield: 61%)

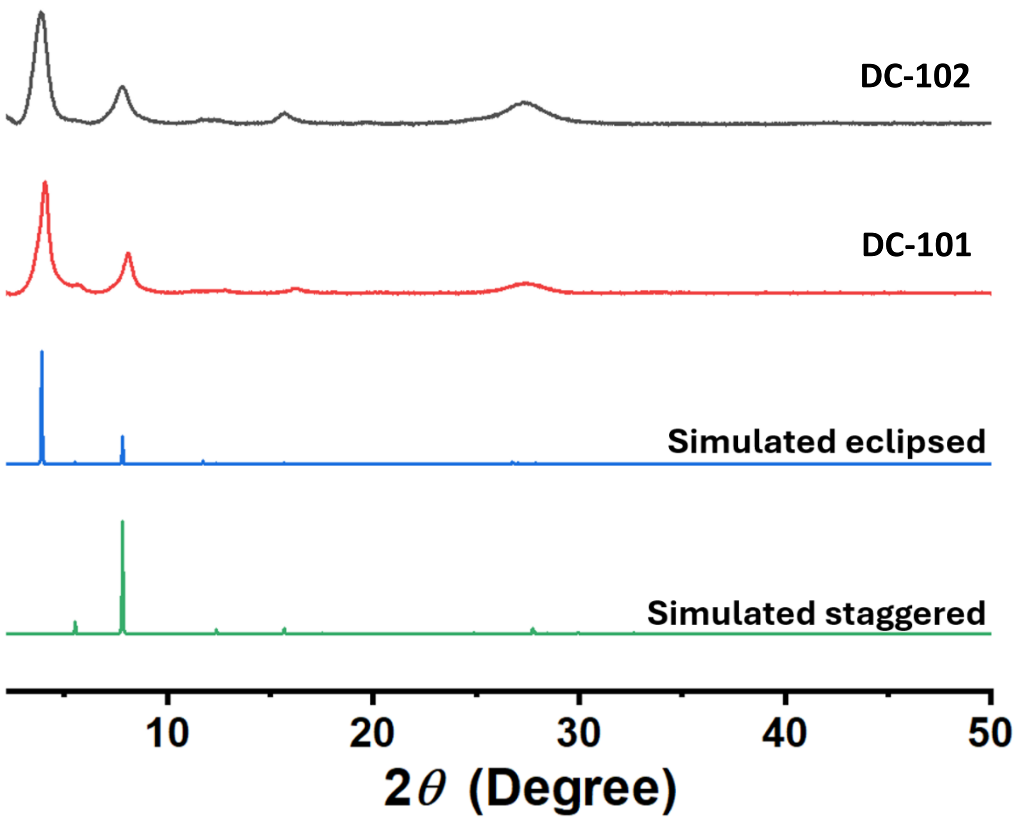

Figure S8. PXRD spectra of **DC-101** and **DC-102**.

3. Brunauer–Emmett–Teller (BET) Analysis

In order to assess the porosity of the MOFs, gas adsorption measurements were performed on an ASAP Plus 2020 (Mircromeritics, Norcross, Georgia) 3FLEX instrument with N_2 at 77K. To remove the residual high boiling point solvent used in the synthesis of the MOFs, the samples of MOFs were activated by soaking in DMSO for 2 days during which the DMSO was changed every 24 hours and then in THF for 1 day. The samples were then dried in the oven under vacuum (20 mTorr, 70 °C for 24 hours). Before gas adsorption measurements, the samples were degassed under vacuum at 85 °C for 12 hours. For BET calculations, a full isotherm with a fitting range of 0 to 0.3

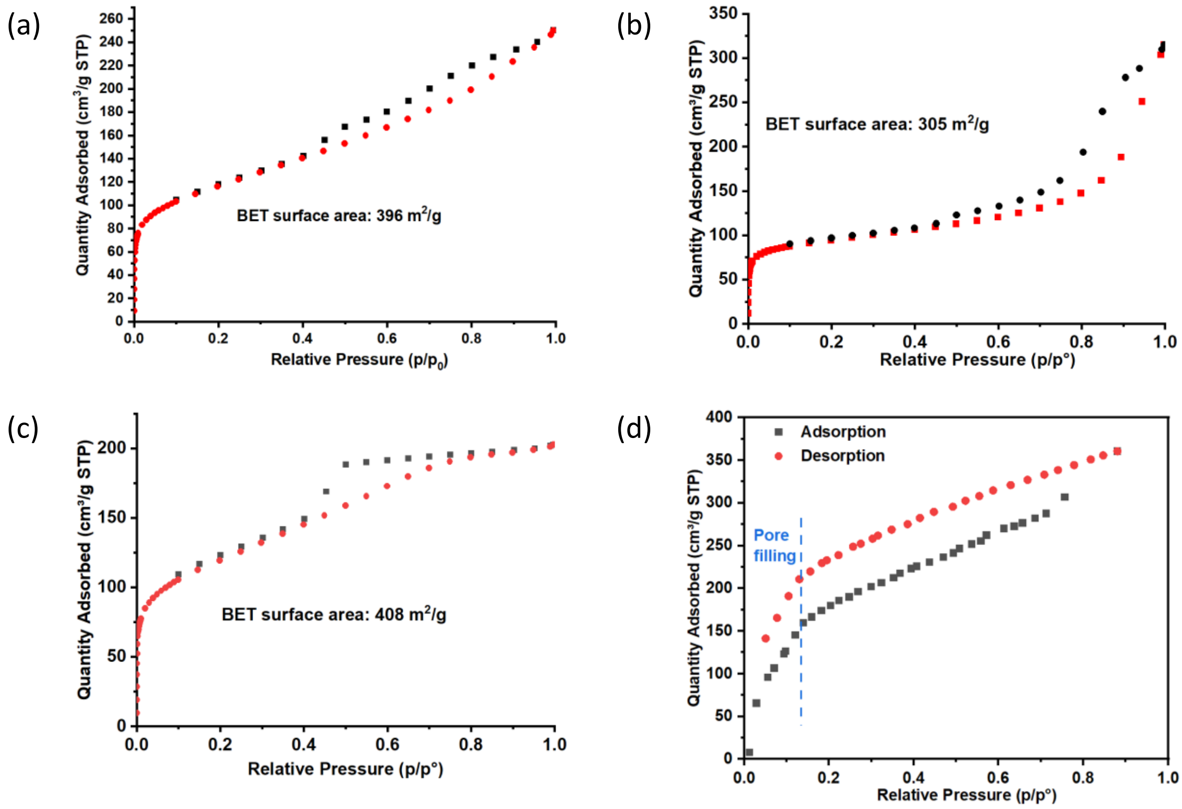

Figure S9. Nitrogen sorption curves (Red dots: adsorption, black dots: desorption, STP=standard temperature pressure) at 77 K of activated (a) **DC-100**, (b) **DC-101**, and (c) **DC-102**. (d) Water sorption curves at 298 K of activated **DC-100**.

4. Measurements of Conductivity

To make a pressed pellet, 25 mg of the MOF sample was put into a 6 mm inner-diameter split sleeve pressing die and pressed for 10 min under a pressure of approximately 1000 psi. A Signatone tungsten carbide four-point linear probe was employed to collect bulk conductivity measurements of the MOFs with a space between tips of 1.25 mm. We calculated the bulk conductivity measurements (S/cm) using the equation $\sigma = \frac{I}{v}\frac{1}{2\pi sF}$. Herein, I (A) is current, V is the voltage of cross the probes, s (cm) is distance of between the probes (1.25 mm), F (unitless) is the correction factor accounting for the diameter and thickness of the pellet.[R3]

Conductivities of the **DC-100**, **DC-101**, and **DC-102** measured by four-point probe method were 2.6 ± 1.3 x 10^{-6} S cm^{-1} (n = 7), 1.9 ± 0.8 x 10^{-6} (n = 7), and 2.0 ± 0.6 x 10^{-7} (n = 7) respectively.

5. X-ray photoelectron spectroscopy (XPS)

X-ray photoelectron spectroscopy (XPS) experiments were conducted on a Physical Electronics Versaprobe II X-ray Photoelectron Spectrometer under ultrahigh vacuum (base pressure 10^{-10} mbar). The measurement chamber was equipped with a monochromatic Al (Kα) X-ray source. Both survey and high-resolution spectra were obtained using a beam diameter of 200 μm. The spectra were processed with CasaXPS.

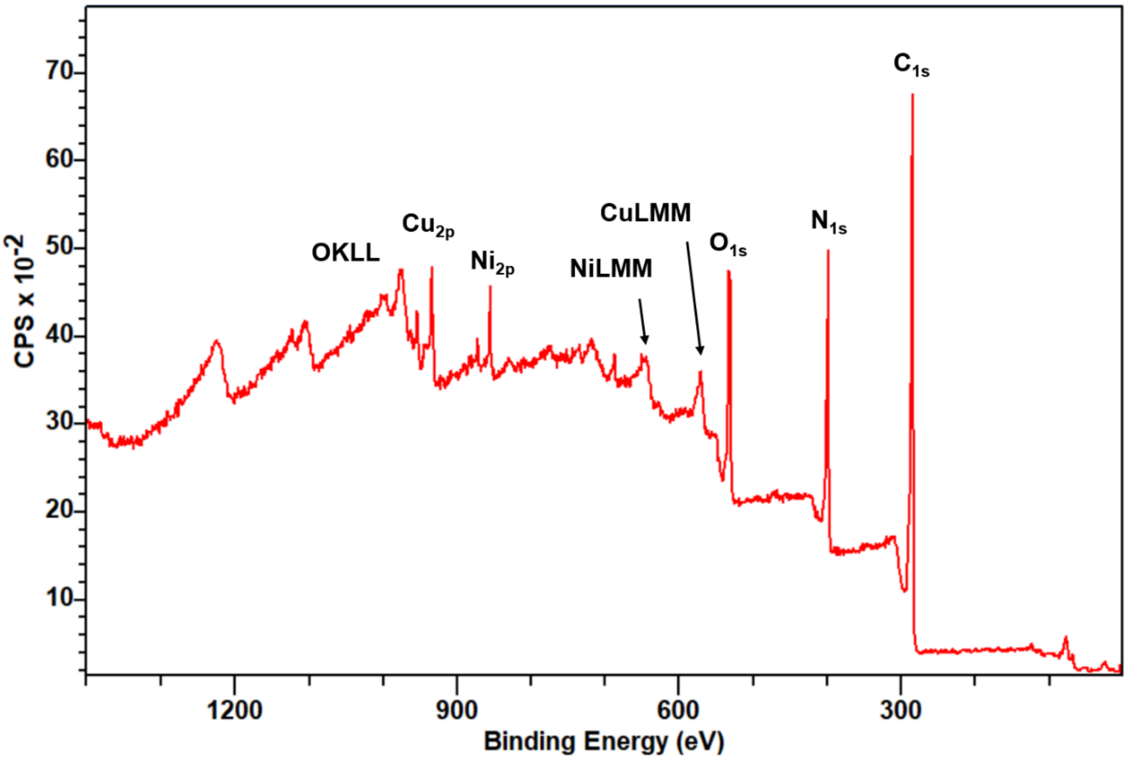

Figure S10. XPS survey spectrum of **DC-100** showing the presence of C, N, O, Ni and Cu elements.

Table S2. Chemical composition in the XPS of **DC-100**.

Peak identification	C 1s	N 1s	O 1s	Ni 2p	Cu 2p
**Position (eV)**	285.0	398.5	532.0	855.0	934.0
**Area (%)**	44.6	22.1	23.4	3.3	6.6

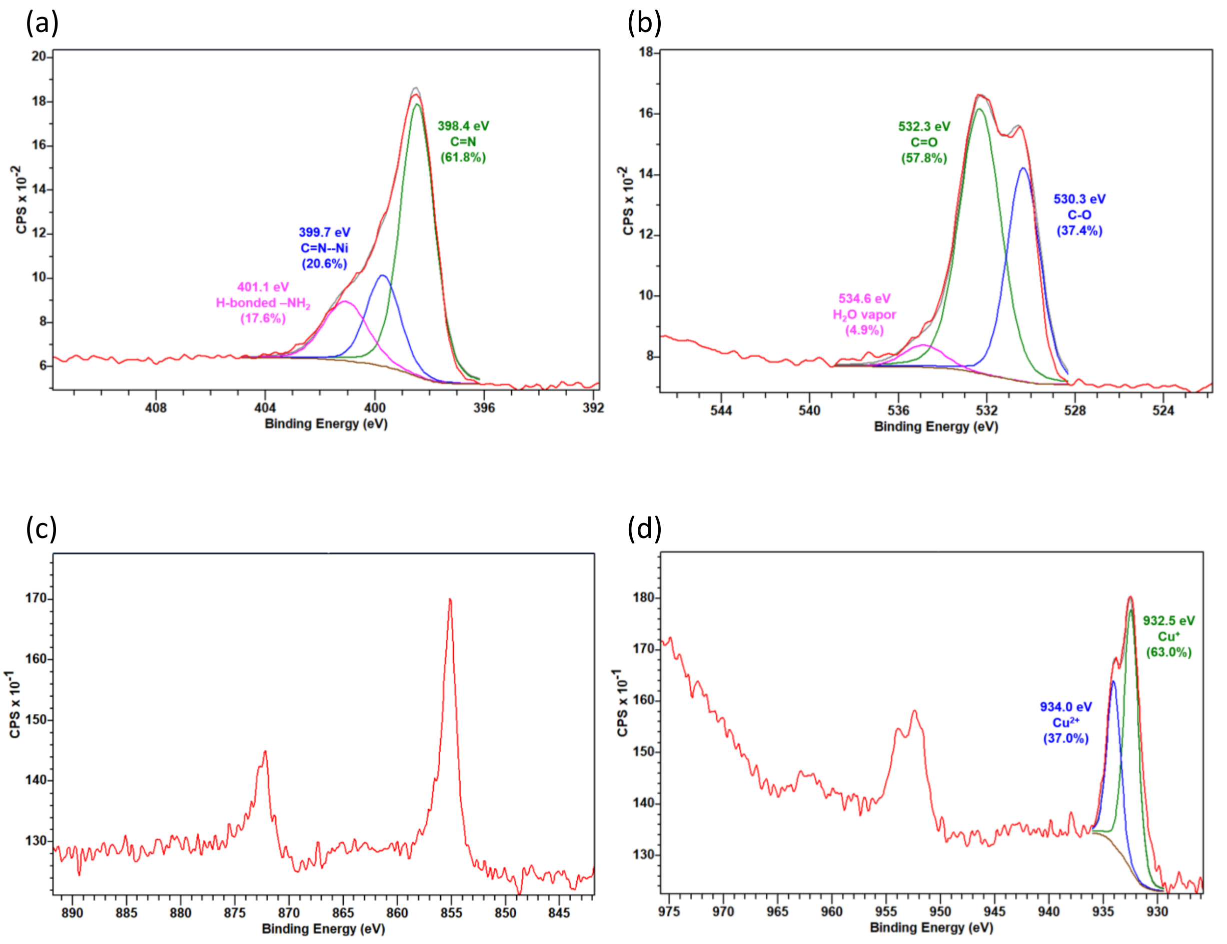

Figure S11. Deconvoluted XPS spectra of the **DC-100**: (a) N 1s, (b) O 1s, (c) Ni 2p, and (d) Cu 2p. The red, brown, and grey curve represent original data, background and the sum of the deconvoluted peaks, respectively.

XPS analysis confirmed the presence of C, O, N, Ni, and Cu in **DC-100** (Figure S10), consistent with the expected elemental composition. The Ni 2p to Cu 2p peak area ratio of 1:2 (Table S2) closely matched the theoretical 1:2 ratio expected from NiTPz-$(OH)_8$ polymerization with Cu(II) ions. High-resolution Cu 2p scans revealed two peaks: Cu $2p_{3/2}$ (932.5 eV) and Cu $2p_{1/2}$ (952.5 eV). The Cu $2p_{3/2}$ peak comprised two components [932.5 eV for Cu(I) and 934.0 eV for Cu(II)][R35] with an area ratio of 6:4, indicating substantial Cu(II) reduction. This finding aligns

with the EPR spectrum, which showed a peak at g = 2.1, confirming the presence of paramagnetic Cu(II) ions [Figure S14(a)]. O 1s scans revealed two environments: C-O (530.3 eV) and C=O (532.3 eV) in a 4:6 ratio [Figure S11(b)],[R36] suggesting a semiquinone structure for each dihydroxyquinoxaline unit of TPz. N 1s scans [Figure S11(a)] showed three environments: non-coordinated N (398.4 eV), Ni-coordinated N (399.7 eV),[R37] and hydrogen-bonded primary amine (401.1 eV)[R38] in a 3:1:1 ratio, indicating two ethylenediamine molecules per TPz ligand. Based on these analyses, we propose a charge neutral skeleton with the formula $(NiTPz)_1Cu_2{\cdot}2EDA$.

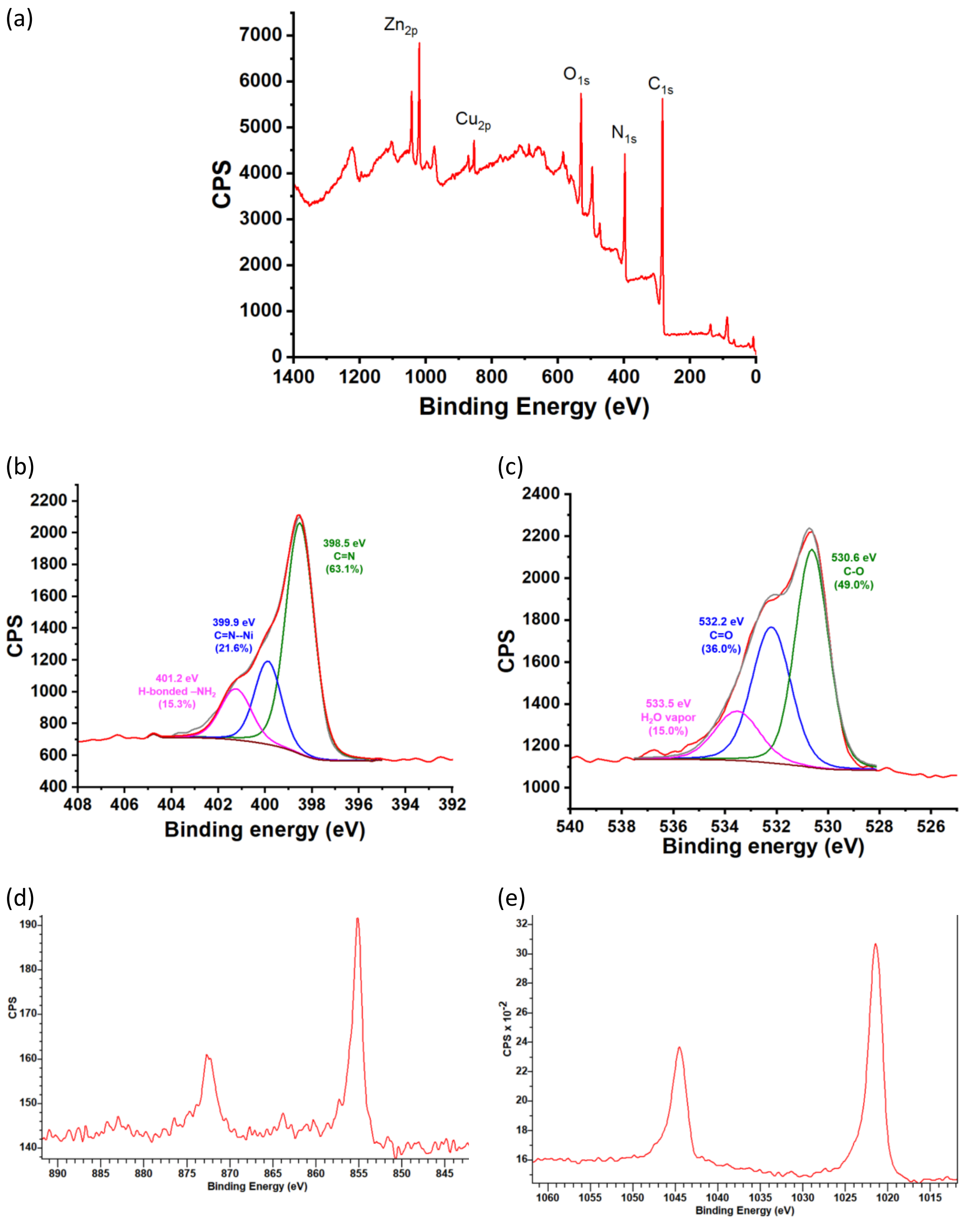

Figure S12. (a) XPS survey spectrum of **DC-101** showing the presence of C, N, O, Ni and Zn elements. Deconvoluted XPS spectra of the **DC-101**: (b) N 1s, (c) O 1s, (d) Ni 2p, and (e) Zn 2p. The red, brown, and grey curve represent original data, background and the sum of the deconvoluted peaks, respectively.

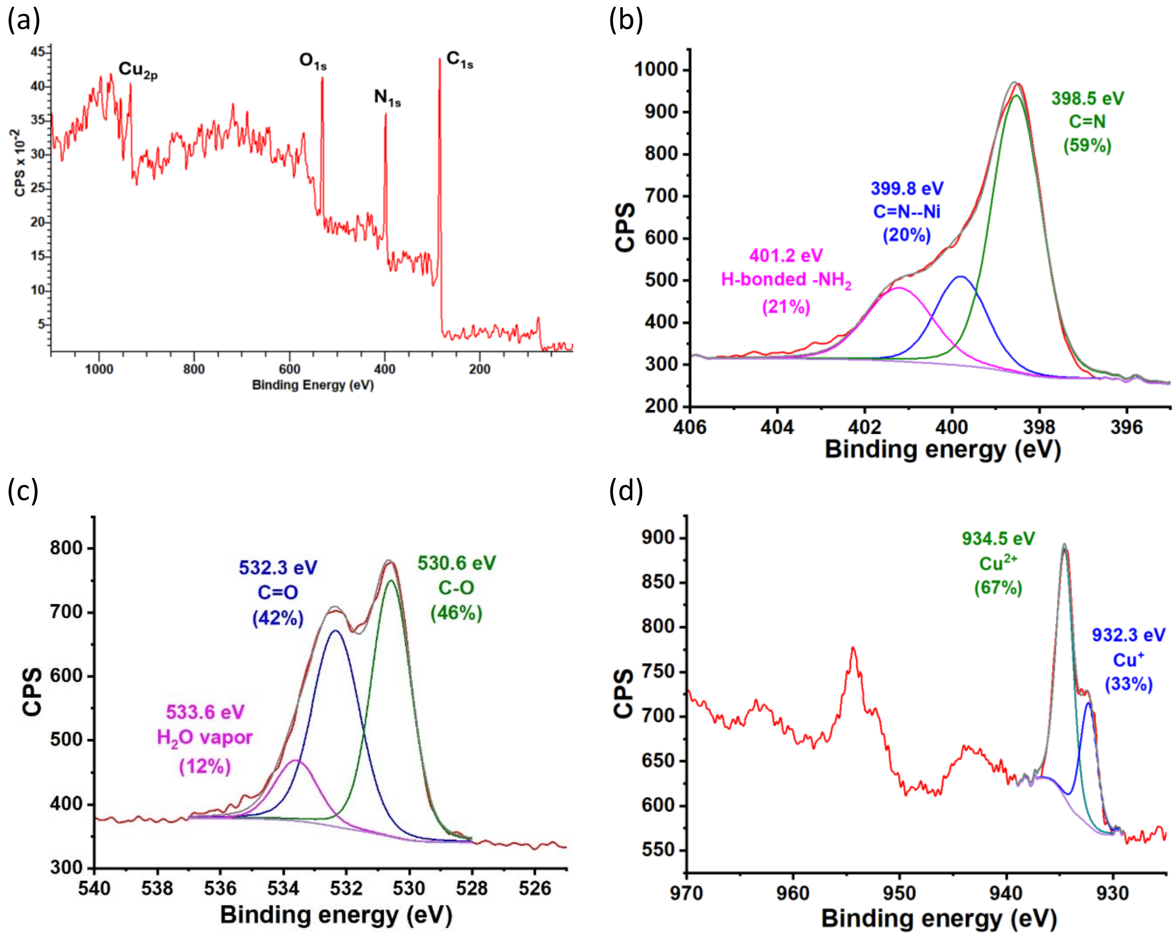

Figure S13. (a) XPS survey spectrum of **DC-102** showing the presence of C, N, O, and Cu elements. Deconvoluted XPS spectra of the **DC-102**: (b) N 1s, (c) O 1s, and (d) Cu 2p. The red, purple, and grey curve represent original data, background and the sum of the deconvoluted peaks, respectively.

6. Elemental analysis

Elemental analyses, including C, H, N, and S were performed by Atlantic Microlab inc. using combustion method by automatic analyzers. The metal contents (Ni, Cu & Zn) were analyzed by ICP-MS. The results are listed below.

Table S3. Elemental analysis of **DC-100**

Elemental	Found (2)	Theoretical[1] (3)	Diff. (3) – (2)	Theoretical[2] (5)	Diff. (5) – (2)
**C**	44.53	46.81	+2.28	43.60	-0.93
**H**	3.73	0.79	-2.94	2.68	-1.05
**N**	21.83	21.83	0	22.60	+0.77
**S**	1.07	0	-1.07	1.3	+0.23
**Ni**	4.44	5.72	+1.28	4.73	+0.29
**Cu**	9.98	12.38	+2.40	10.25	+0.27
**O**	-	12.47	-	14.84	-

[1] Based on the formula $(NiTPz)_1Cu_2$

[2] Based on the formula $(NiTPz)_1Cu_2$ + 2 EDA + 3 H_2O + 0.5 DMSO

Elemental analysis and ICP-MS results deviated by more than 2% from the theoretical $(NiTPz)_1Cu_2$ formula (Table S3, column 4). Considering the use of high-boiling-point DMSO, hydrophilic nature of the MOF, and the N 1s XPS peak ratios, we propose the refined formula $(NiTPz)_1Cu_2\cdot 2EDA\cdot 3H_2O\cdot 0.5DMSO$. This composition aligns well with experimental data, showing ≤1% error (Table S3, column 6).

Table S4. Elemental analysis of **DC-101**

Elemental	Found (2)	Theoretical[1] (3)	Diff. (3) – (2)	Theoretical[2] (5)	Diff. (5) – (2)
**C**	40.37	46.64	6.27	40.29	-0.08
**H**	3.35	0.78	-2.57	2.47	-0.88
**N**	19.23	21.76	2.53	19.48	0.25
**S**	0	0	0	0	0
**Ni**	4.28	5.70	0.85	4.80	0.52
**Zn**	10.67	12.69	0.59	10.7	0.03
**O**	-	12.43	-	22.25	-

[1] Based on the formula $(NiTPz)_1Zn_2$

[2] Based on the formula $(NiTPz)_1Zn_2$ + 0.5 EDA + 9 H_2O

Table S5. Elemental analysis of **DC-102**

Elemental	Found (2)	Theoretical[1] (3)	Diff. (3) – (2)	Theoretical[2] (5)	Diff. (5) – (2)
**C**	43.86	49.54	5.68	42.97	-0.89
**H**	3.44	1.04	-2.4	2.92	-0.52
**N**	20.67	23.11	2.44	21.48	0.81
**S**	0.14	0	-0.14	0	-0.14
**Cu**	10.81	13.11	1.3	10.83	0.02
**O**	-	13.20	-	21.81	-

[1] Based on the formula $(H_2TPz)_1Cu_2$

[2] Based on the formula $(H_2TPz)_1Cu_2$ + EDA + 8 H_2O

7. Electron paramagnetic resonance spectroscopy

EPR spectra were collected on a Bruker BioSpin Gmbh spectrometer equipped with a standard mode cavity. For each sample, about 2 mg material was used. The samples were flushed with N_2 for 15 minutes and then sealed in the EPR tube under N_2 atmosphere. The EPR spectrum was collected in room temperature. **DC-100** and **DC-102** showed EPR signal at g=2.101 and 2.088 from the Cu(II) unpaired electron respectively.

(a)
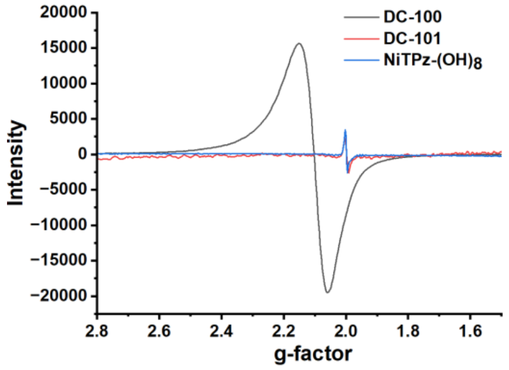

(b)
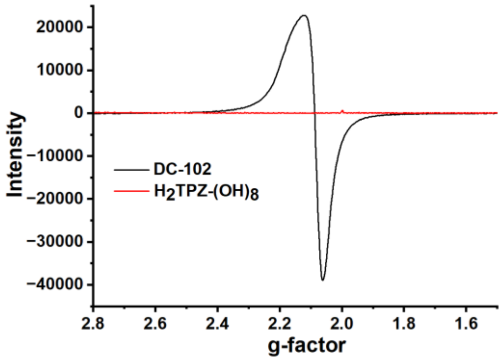

Figure S14. EPR spectra of (a) **DC-100** (black line), **DC-101** (red line) and NiTPz-$(OH)_8$ (blue line), (b) **DC-102** (black line) and H_2TPz-$(OH)_8$ (red line).

8. Thermal Gravimetric Analyses

Thermal gravimetric analysis was performed using a TA Instruments TGA Q150 with a 5 ^{o}C/min ramp from room temperature to 900 ^{o}C.

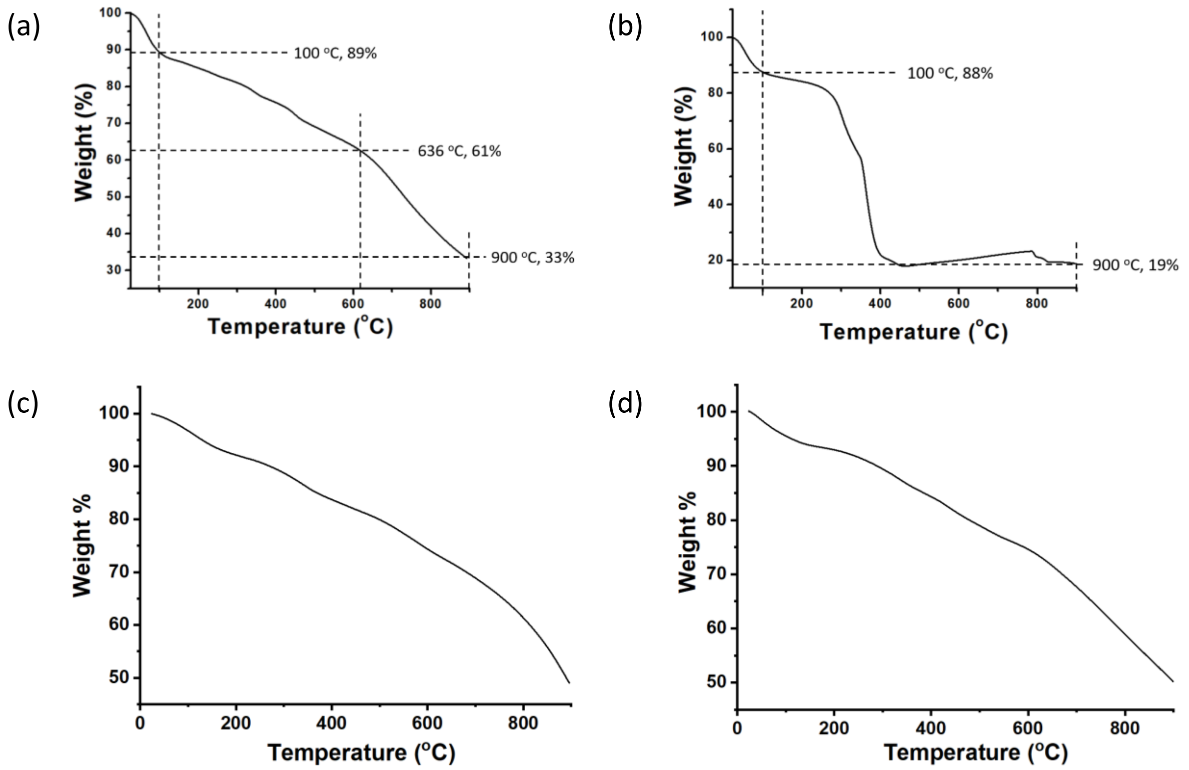

Figure S15. TGA curves of (a) **DC-100** in nitrogen and (b) in air, (c) **DC-101** in nitrogen and (d) **DC-102** in nitrogen.

TGA curves in nitrogen and air [Figure S15(a) and S15(b)] support this proposed composition $(NiTPz)_1Cu_2 \cdot 2EDA \cdot 3H_2O \cdot 0.5DMSO$. Initial mass losses of 11–12% at 100°C correspond well to the volatile components (EDA and water, 14% of the proposed composition). At 900°C in air, the remaining mass was 19% [Figure S15(b)]. After correcting for initial mass loss and assuming that only NiO and CuO remain, the experimental mass (22%) closely matches the theoretical weight percentage of NiO and CuO (23%).

9. SEM and TEM

Scanning electron microscopy was obtained using a Hitachi TM3000 SEM. The material was pressed onto carbon conductive tape that was attached to the aluminum plate. The images were taken at a 10 mm working distance using a 15 kV beamline in a to 10^{-6} torr vacuum chamber. Transmission electron microscopy was carried out in a Tecnai F20ST FEG TEM instrument. The sample was prepared by drop casting a water suspension (0.5 mg in 5 mL) of the MOF onto a copper grid (300 mesh, 3.0 mm O.D). An operating voltage of 120 kV was used for imaging. Energy dispersive X-Ray spectroscopy was collected using SDD X-ray microanalysis system with Octane Pro 10 sq. mm detector and TEAM software.

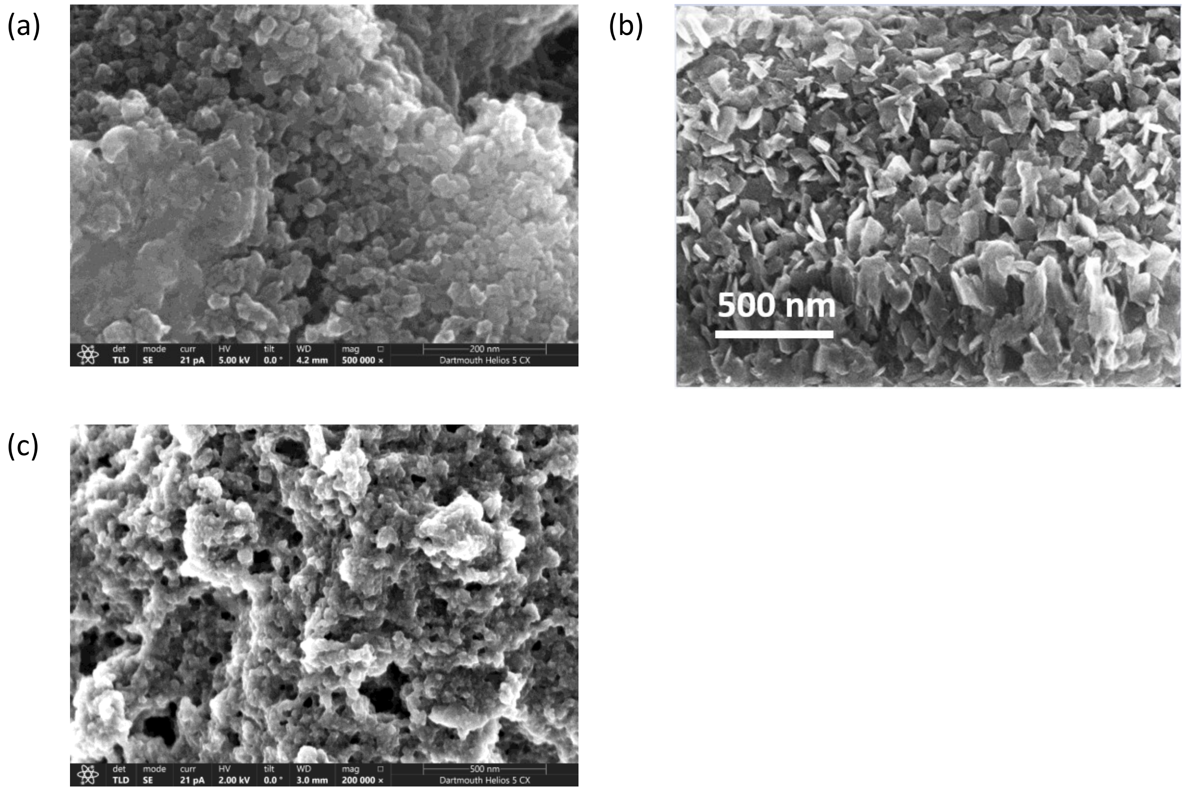

Figure S16. SEM images of (a) **DC-100**, (b) **DC-101** and (c) **DC-102**.

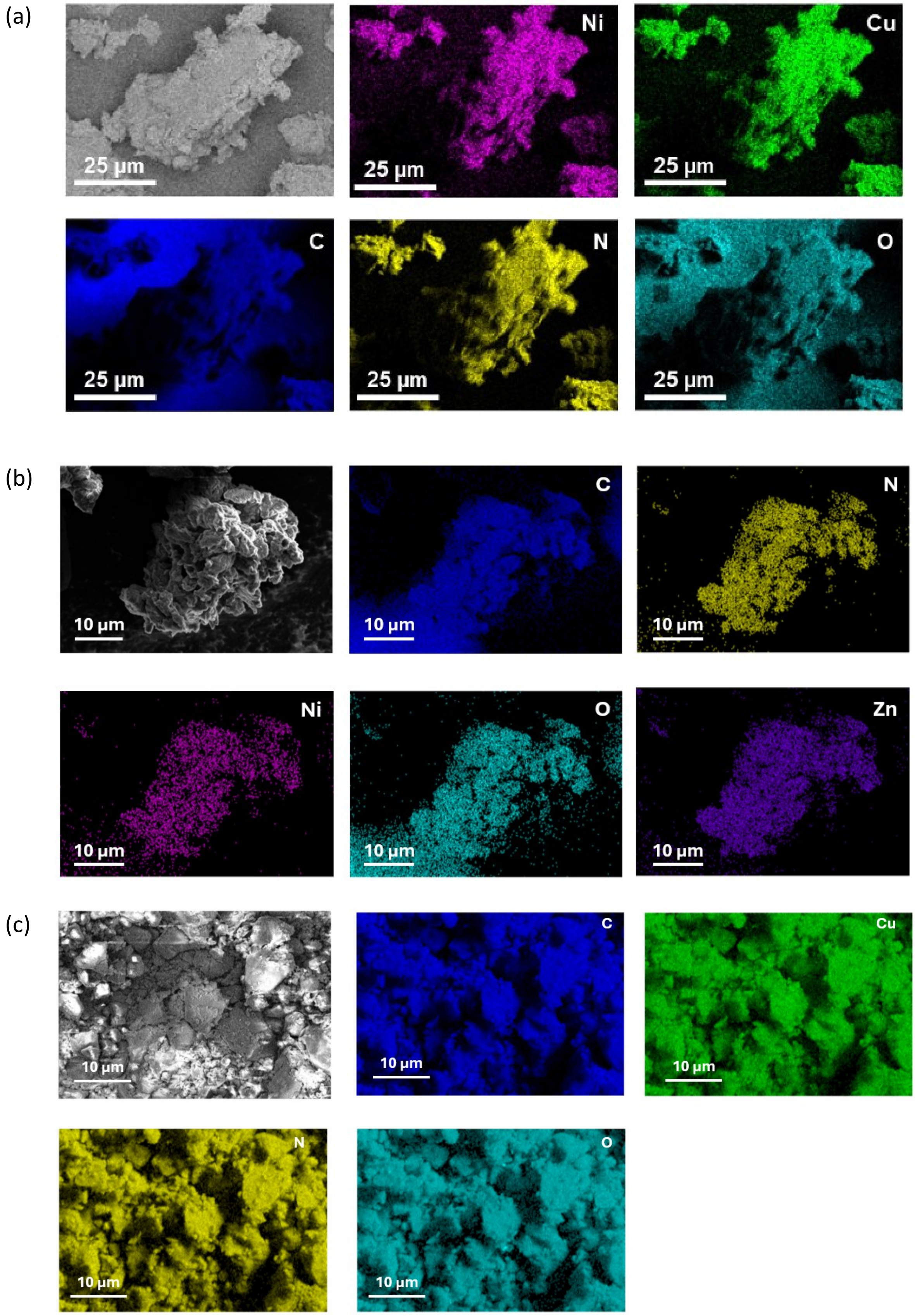

Figure S17. SEM EDX mapping of (a) **DC-100,** (b) **DC-101,** and (c) **DC-102.**

Table S6. Elemental counting of (a) **DC-100,** (b) **DC-101,** and (c) **DC-102** by SEM EDX.

Element	C	N	O	Ni	Cu	Zn	S
**(a) Counting (%)**	66.9	19.5	8.8	1.5	3.1	-	0.2
**(b) Counting (%)**	59.3	14.6	12.3	3.8	-	10.0	-
**(c) Counting (%)**	64.8	21.1	9.8	-	4.2	-	0.1

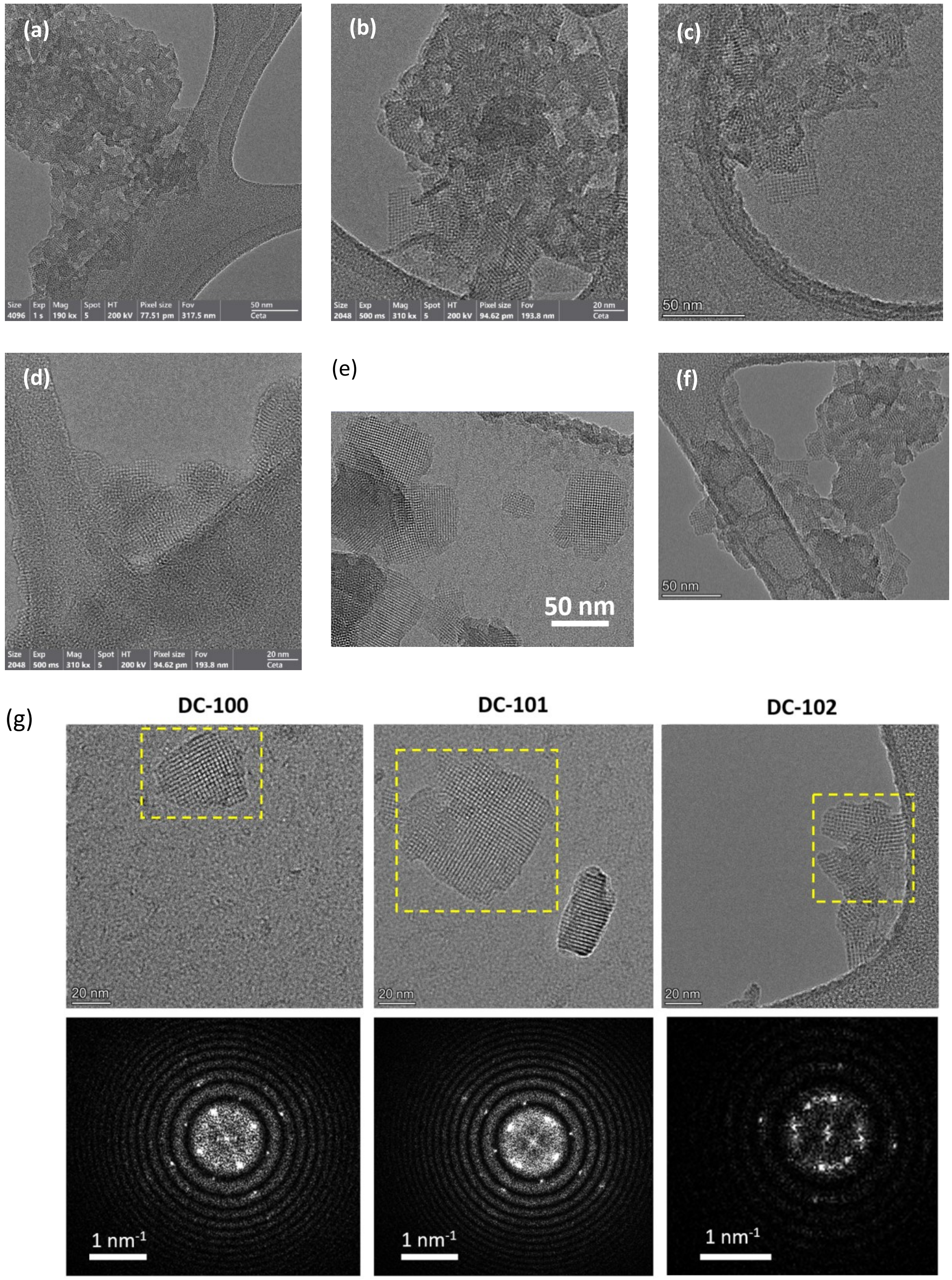

Figure S18. TEM images of (a–d) **DC-100**, (e) **DC-101**, and (f) **DC-102**. (g) The characterized region of TEM images (yellow box) and the corresponding fast Fourier transform (FFT) diffraction patterns of **DC-100**, **DC-101**, and **DC-102**.

10. ATR-FTIR spectroscopy

Infrared spectra were collected using a JASCO model FT IR-6100 Fourier transform infrared spectrophotometer.

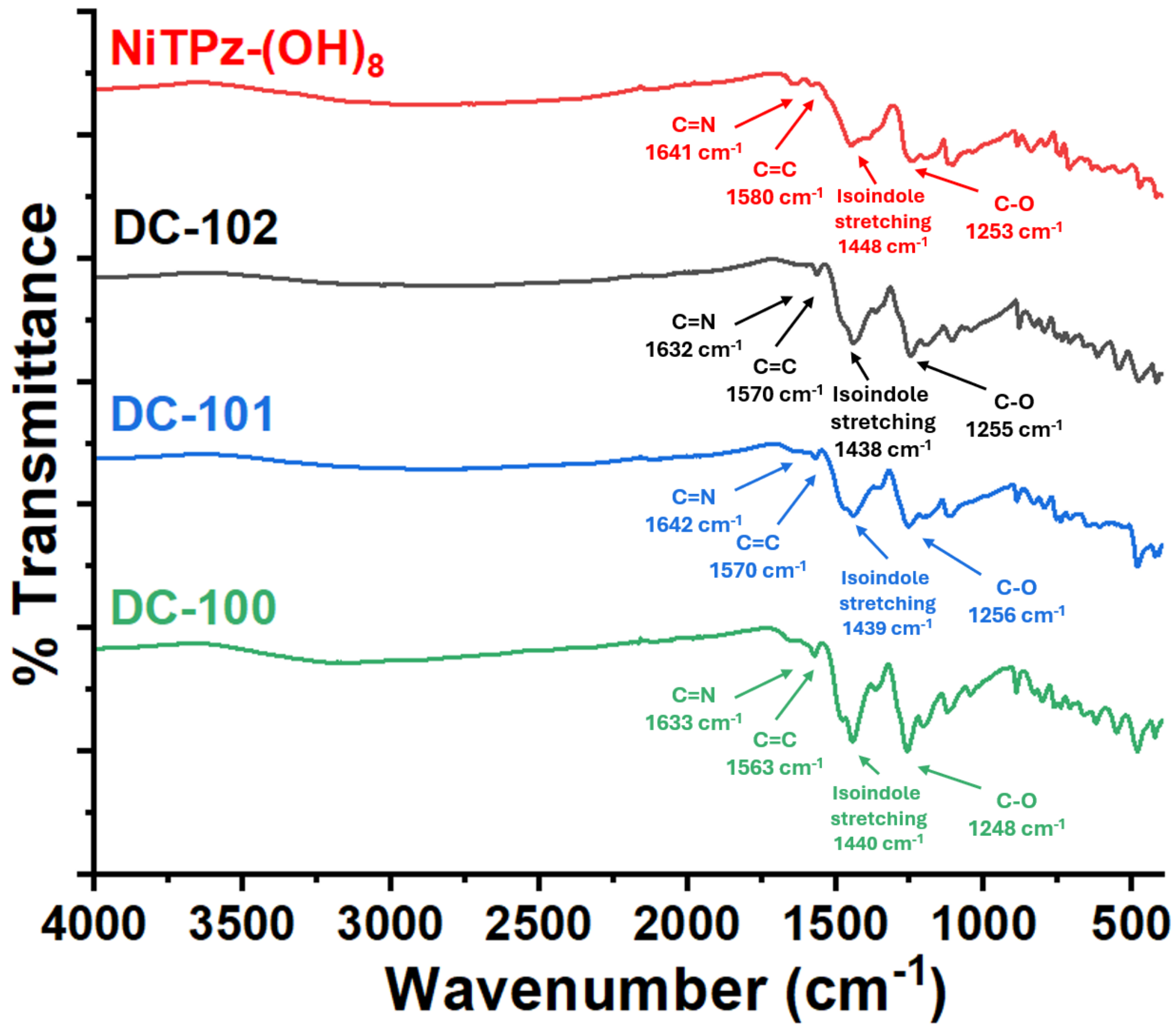

Figure S19. ATR-FTIR spectra of the NiTPz-$(OH)_8$, **DC-100**, **DC-101**, and **DC-102.**

11. Dye uptake experiments

The dye adsorption behavior of **DC-100** was carried out with two different kinds of dyes, anionic dye (methyl orange, MO) and cationic dye (methylene blue, MB). The dye adsorption experiments were performed by immersing 2 mg of **DC-100** in 10 mL of 1×10^{-5} M aqueous solutions of dyes. The adsorption was monitored by the UV-vis spectra of the aqueous solution at various time intervals.

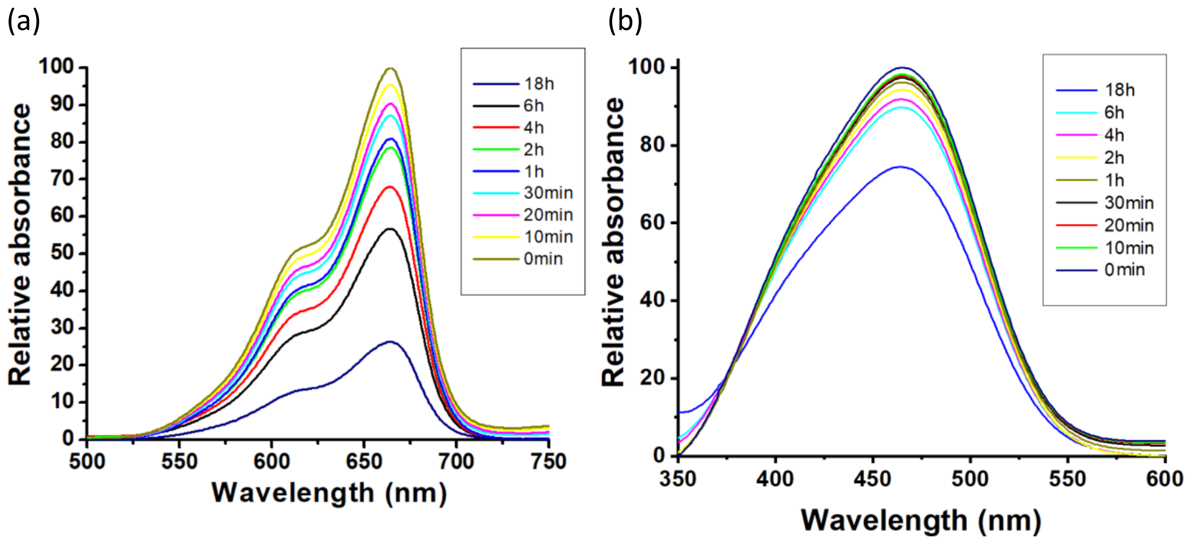

Figure S20. UV-vis spectra for the uptake of (a) methylene blue (MB) and (b) methyl orange (MO) from aqueous solutions at various time intervals by **DC-100**.

12. Sensing experiments

12.1. Fabrication of Gas Sensors

1 mg of **DC-100** was dispersed in 1 mL of deionized water. The mixture was then sonicated for 90 minutes and a homogeneous suspension was obtained. 25 µL of the suspension was drop casted onto interdigitated gold electrodes with 5 µm gaps (part NO. G-IDEAU5, purchased form Metrohm), which was then allowed to dry in the air under room temperature for overnight before use. The resistance values of electrodes in the range of 100-200 kΩ. Images of the electrodes are shown in **Figure S22**.

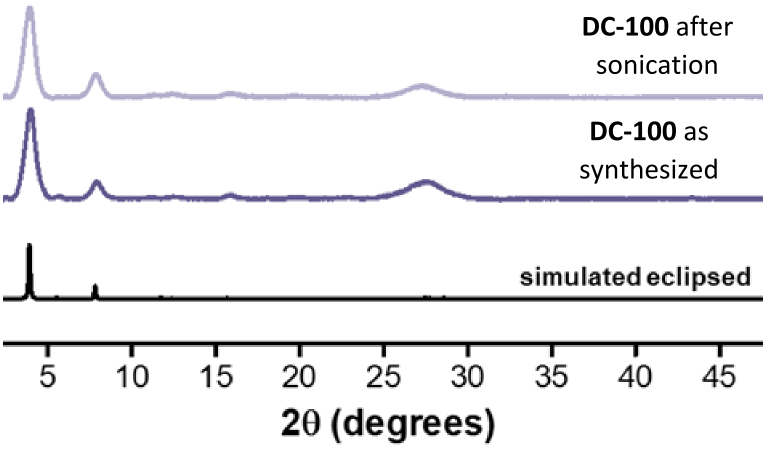

Figure S21. PXRD spectra of **DC-100** as-synthesized and after sonication for around 90 minutes.

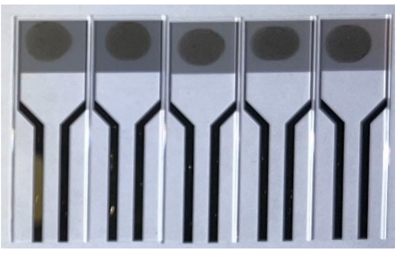

Figure S22. Representative photographs of the electrodes after dropcasting and drying the MOF suspensions overnight.

12.2. Sensing Experiments

The sensing performance of the fabricated devices was monitored in a sealed gas-sensing chamber at room temperature. An electrical feed-through and gas inlet and outlet were installed in the chamber. Dry N_2 was used as the balance/purging gas. A Sierra Micro-Trak and a Smart-Trak mass flow controllers were used in combination to deliver controlled concentrations of gases from custom-ordered pre-mixed tanks (100 ppm of NO in N_2, 10000 ppm of H_2S, SO_2, CO, and NH_3 in N_2, and 10000 ppm of NO_2 in air) equipped with two-stage stainless steel regulators. Gas streams from the tanks were diluted with dry N_2 for delivery of controlled concentrations of gases. The concentrations of target gases were adjusted by controlling the flow rates of the target gas and balance gas, which was achieved using two mass-flow controllers. The system was limited to mixing of one gas at a time with a dry N_2 or humidified N_2 stream. The flow of balance/purging gas was controlled at 0.4 or 1 L/min and the flow of the analyte was controlled at 0.1 to 4 mL/min. Before target gas exposure, the gas sensors were stabilized under N_2 exposure for 5 minutes to obtain a flat base line. All sensing experiments were performed under a constant applied voltage of 1V.

Raw current data (collected under constant applied voltage) was normalized and converted to normalized conductance according to the equation $-\frac{\Delta G}{G_0} = -\frac{I - I_0}{I_0} \times 100\%$, wherein I_o = initial current and I = current at various points during measurement.

A FlexStream Trace Gas Standards Generator equipped with a humidity sensor was used to produce the humid gas stream. This generator delivered a wet nitrogen flow of 1 L/min at 20% relative humidity (RH). To achieve the desired humidity level, this wet nitrogen was diluted with 1 mL/min of dry nitrogen, controlled by a mass flow controller. The resulting diluted vapor was then mixed with a controlled stream containing the NO gaseous analyte.

This setup allowed for the delivery of a humidified stream containing 1 ppm of NO for the sensing experiments.

The theoretical limits of detection (LOD) were calculated using reported protocols.[R4] First, the root mean squared (rms) value — representing the noise-based deviation in $-\Delta G/G_o$ — was calculated using the baseline trace before exposure to analyte. We took 600 consecutive points (N) and fit the data to a polynomial (5th order). We then calculated sum of squared residuals (SSR) from equation $SSR = \sum(yi - y)^2$, where yi is measured $-\Delta G/G_o$ and y is the value calculated from the polynomial fit. The root-mean-square deviation (RMS) was then calculated by the equation $RMS = \sqrt{\frac{SSR}{N}}$. We plotted concentration of analyte versus response ($-\Delta G/G_o$) after a specific exposure time and isolated the range of values wherein this relationship was linear. Linear regression provided an equation of best-fit (slope = m). With these values, we extrapolated the theoretical LOD from the equation $LOD = 3 \times \frac{RMS}{m}$.

The degree of saturation (DS) of the sensor, which is defined as the ratio of the quantity of the analyte adsorbed after a specific amount of time by the sensor to the quantity of the analyte absorbed upon the saturation,[R5] was calculated using the equation $DS\% = \frac{C_t}{C_0} = \frac{I_t - I_0}{I_5 - I_0} \times 100\%$. This calculation was based on the saturation point achieved after 5 minutes exposure and that the current change (ΔI_t) of the device after t min exposure is linear proportional to the quantity of the adsorbed analytes (C_t) by the sensing materials.[R6(a)]

In this equation, I_0, I_1, I_2, I_3, I_4, and I_5 are the current of the devices after analyte dosing for 0, 1, 2, 3, 4, and 5 min exposure, respectively, and C_o is the quantity of the adsorbed analytes upon saturation.

In the NO experiment conducted in air, the rate of NO oxidation in air at 25 °C can be estimated using the following equation: rate = -1.5 × 10^4 [NO]$[O_2]^2$.[R6(b)] At 1 ppm NO in air, the NO concentration remains nearly constant over a 5-minute exposure. The detailed calculations are shown below:

Since 1 ppm by volume = 1×10^{-6} L of NO per 1L of air.

At STP, 1 mol of gas occupies 22.414 L

No. of mol of NO in air = $\frac{1\times10^{-6}}{22.414}$ ≈ 4.46 $\times10^{-8}$ mol

$[NO]_0$ = 4.46×10^{-8} mol / 1L = 4.46×10^{-8} mol/L

Since there is 21% oxygen in air, 1 mol air contains 0.21 mol oxygen.

At STP, 1 mol of gas occupies 22.414 L.

$[O_2]$ = 0.21 / 22.414 = 0.009369 mol/L

Since $[O_2]$ is in large excess, we can treat it as constant, we have a pseudo-second-order reaction:

$$\frac{d[NO]}{dt} = -k[O_2][NO]^2$$

This integrates to:

$$\frac{1}{[NO]_t} - \frac{1}{[NO]_0} = k[O_2]t$$

Plug in $[NO]_0$ = 4.46 × 10^{-8}, t = 300, k = 1.5 × 10^4, $[O_2]$ = 0.009369

$$\frac{1}{[NO]_t} = (1.5 \times 10^4)(0.009369)(300) + \frac{1}{4.46 \times 10^{-8}}$$

$[NO]_t$ = 4.4516 × 10^{-8} mol/L

It shows that the concentration of NO is expected to remain almost constant over a 5-minute exposure.

The potential sources of error in the gas sensing experiments include:

- Accuracy limitations in mass flow controllers (MFCs)**:** The low-flow MFC delivering toxic gas (NO) has an error of 0.4 sccm, and the high-flow MFC delivering nitrogen

background has an error of 0.2 L/min. For a 10 ppb NO concentration (using a 100 ppm NO tank), the low-flow MFC was set to 0.1 sccm and the high-flow MFC to 1 L/min. Consequently, measurement errors at this concentration may arise from inaccuracies in gas delivery rates.

- Fluctuations in gas flow stability: These could affect the homogeneity and consistency of the 10 ppb NO concentration due to variations in either MFC.
- Measurement limitations at low current levels: The detection sensitivity of the DC-100 electrodes may be limited by the resolution and accuracy of the potentiostat used (PalmSens EmStatMUX8-R2; potential resolution: 0.1 mV, accuracy: 0.2%).
- Baseline signal fluctuations: These may be caused by the intrinsic signal-to-noise ratio of DC-100.

12.3. NO sensing performance

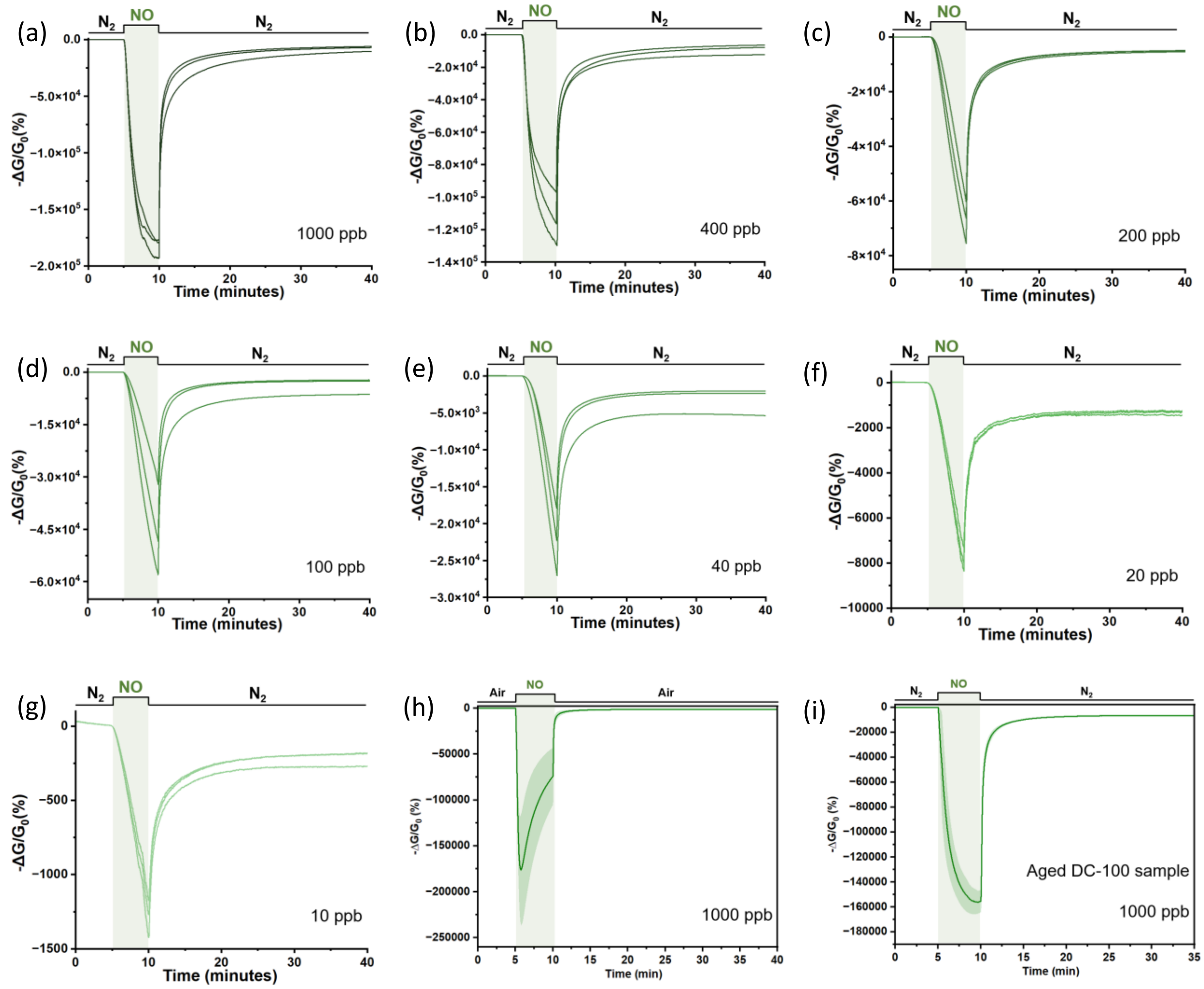

Figure S23. Normalized responses of **DC-100** devices to (a) 1000, (b) 400, (c) 200, (d) 100, (e) 40, (f) 20, (g) 10 ppb of NO in dry N_2; (h) 1000 ppb of NO in dry air; and (i) 1000 ppb of NO in dry N_2 using devices fabricated from aged **DC-100** stored under ambient conditions for 5 months. For each condition, 3 freshly prepared devices were tested.

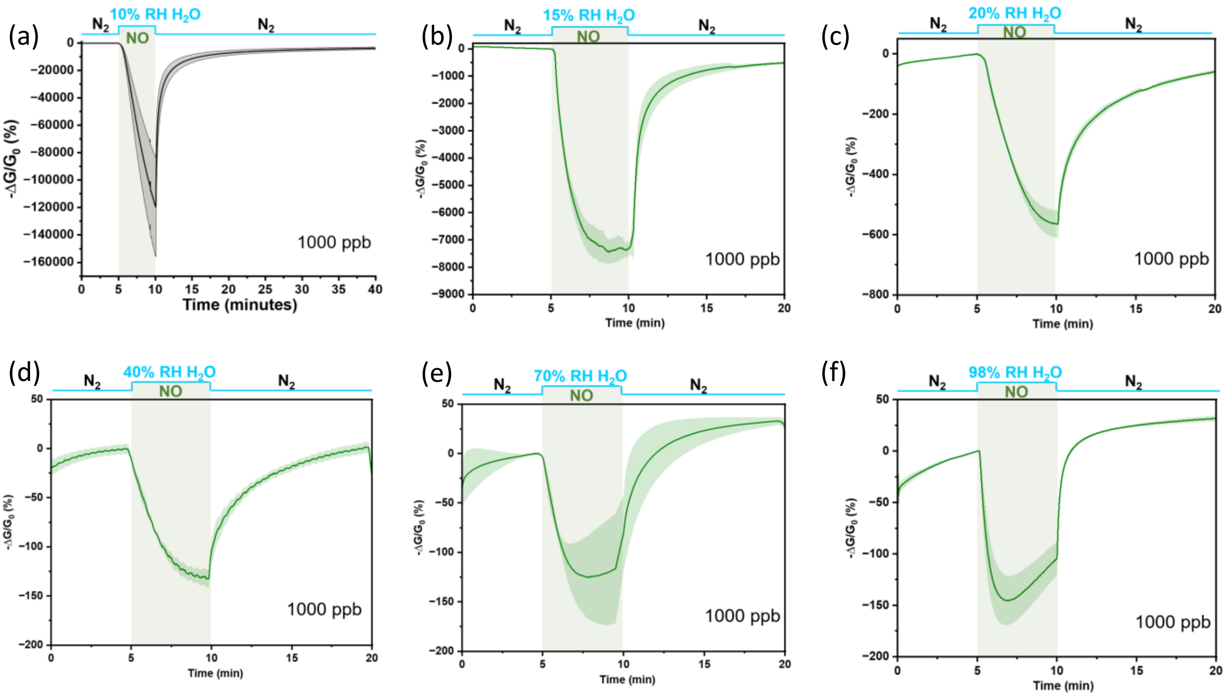

Figure S24. Normalized responses of **DC-100** devices to 1000 ppb of NO in N_2 under (a) 10% RH, (b) 15% RH, (c) 20% RH, (d) 40% RH, (e) 70% RH, and (f) 98% RH.

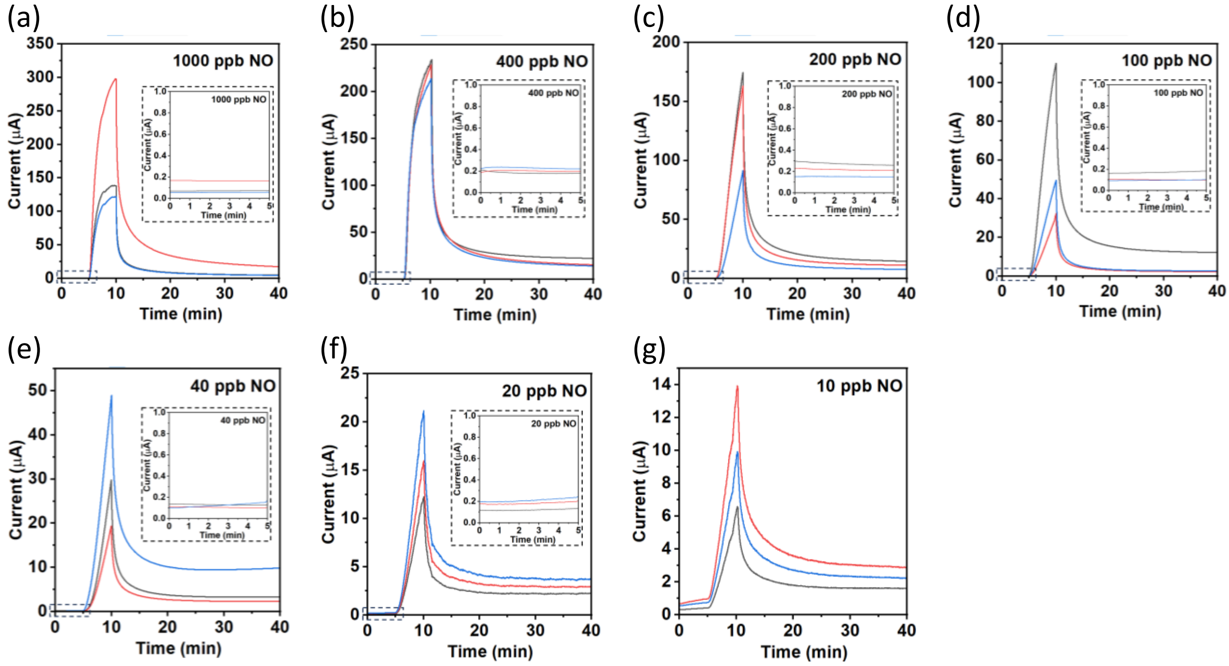

Figure S25. Time-dependent current responses of **DC-100** devices upon exposure to (a) 1000, (b) 400, (c) 200, (d) 100, (e) 40, (f) 20, (g) 10 ppb of NO in dry N_2.

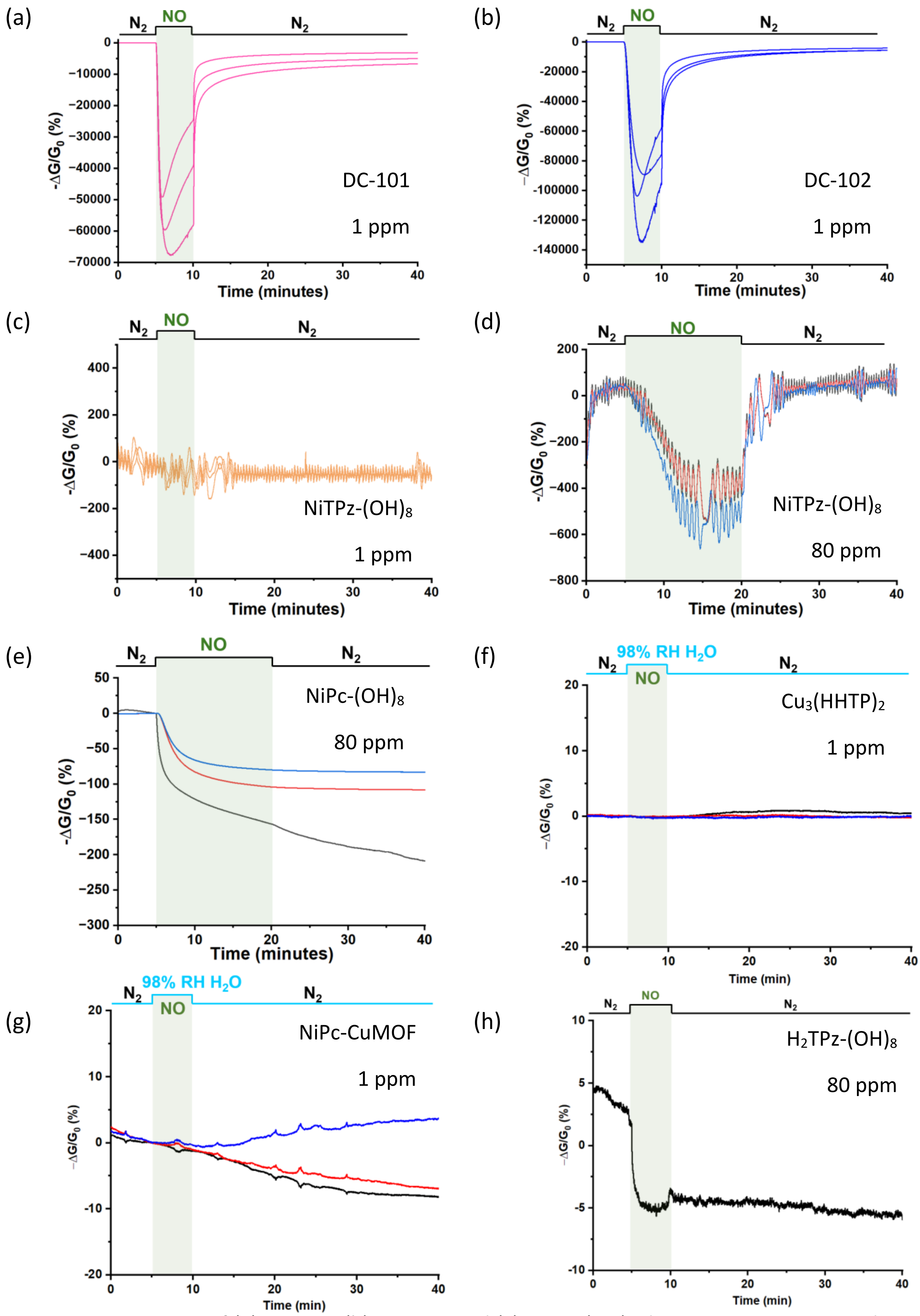

Figure S26. Responses of (a) **DC-101**, (b) **DC-102**, and (c) NiTPz-$(OH)_8$ devices to 1 ppm NO in dry N_2; (d) NiTPz-$(OH)_8$ and (e) NiPc-$(OH)_8$ devices to 80 ppm NO in dry N_2; (f) $Cu_3(HHTP)_2$ and (g) NiPc–CuMOF devices to 1 ppm NO in wet N_2 (98% RH); and (h) H_2TPz-$(OH)_8$ device to 80 ppm NO in dry N_2. For each test, three freshly prepared devices were used, except for panel (h), in which only one device was functional.

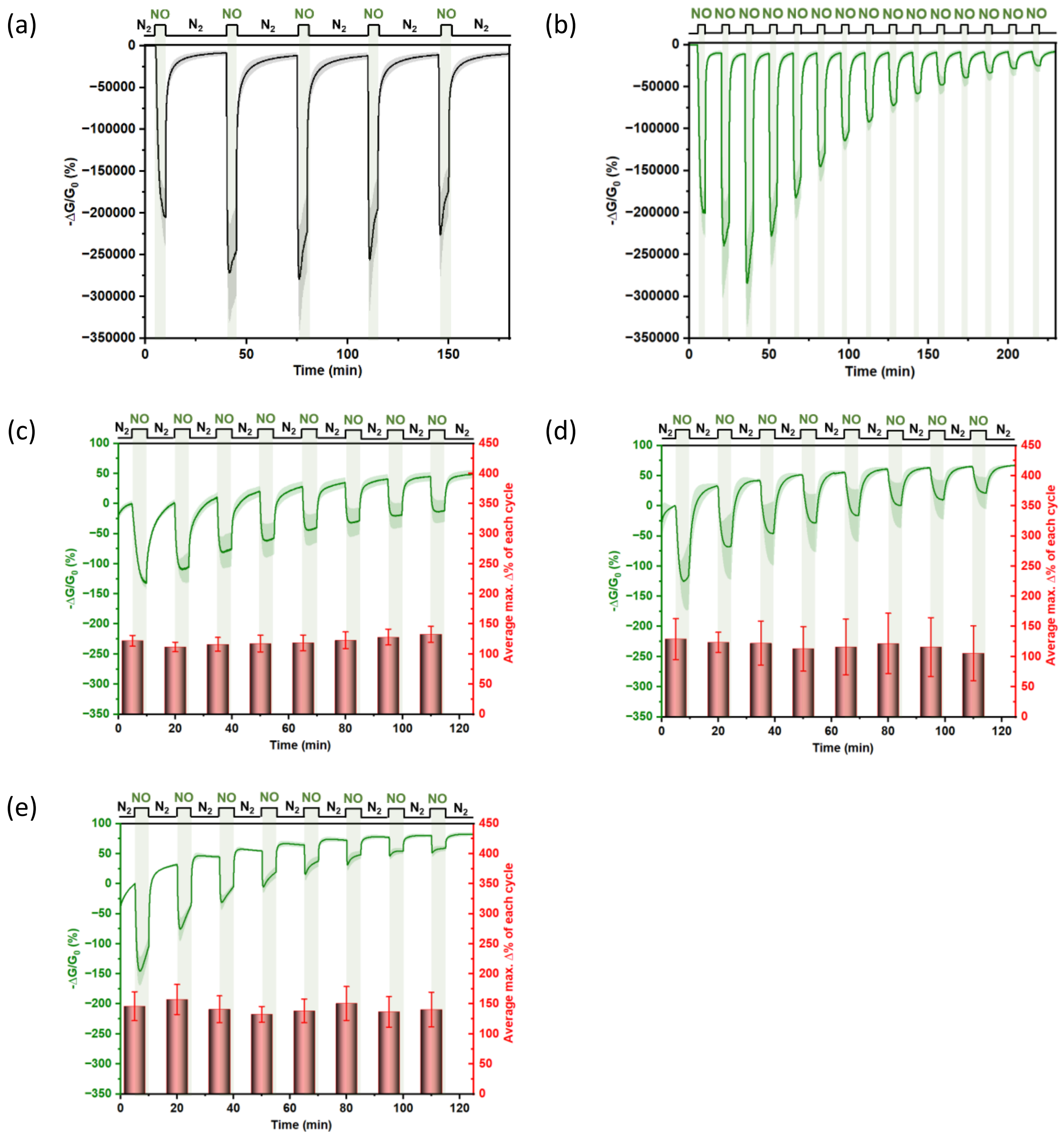

Figure S27. Consecutive exposure–recovery responses of **DC-100** devices to 1 ppm NO under (a) dry N_2 with 5-minute exposure and 30-minute recovery; (b) dry N_2 with 5-minute exposure and 10-minute recovery; wet N_2 at (c) 40% RH, (d) 70% RH, and (e) 98% RH.

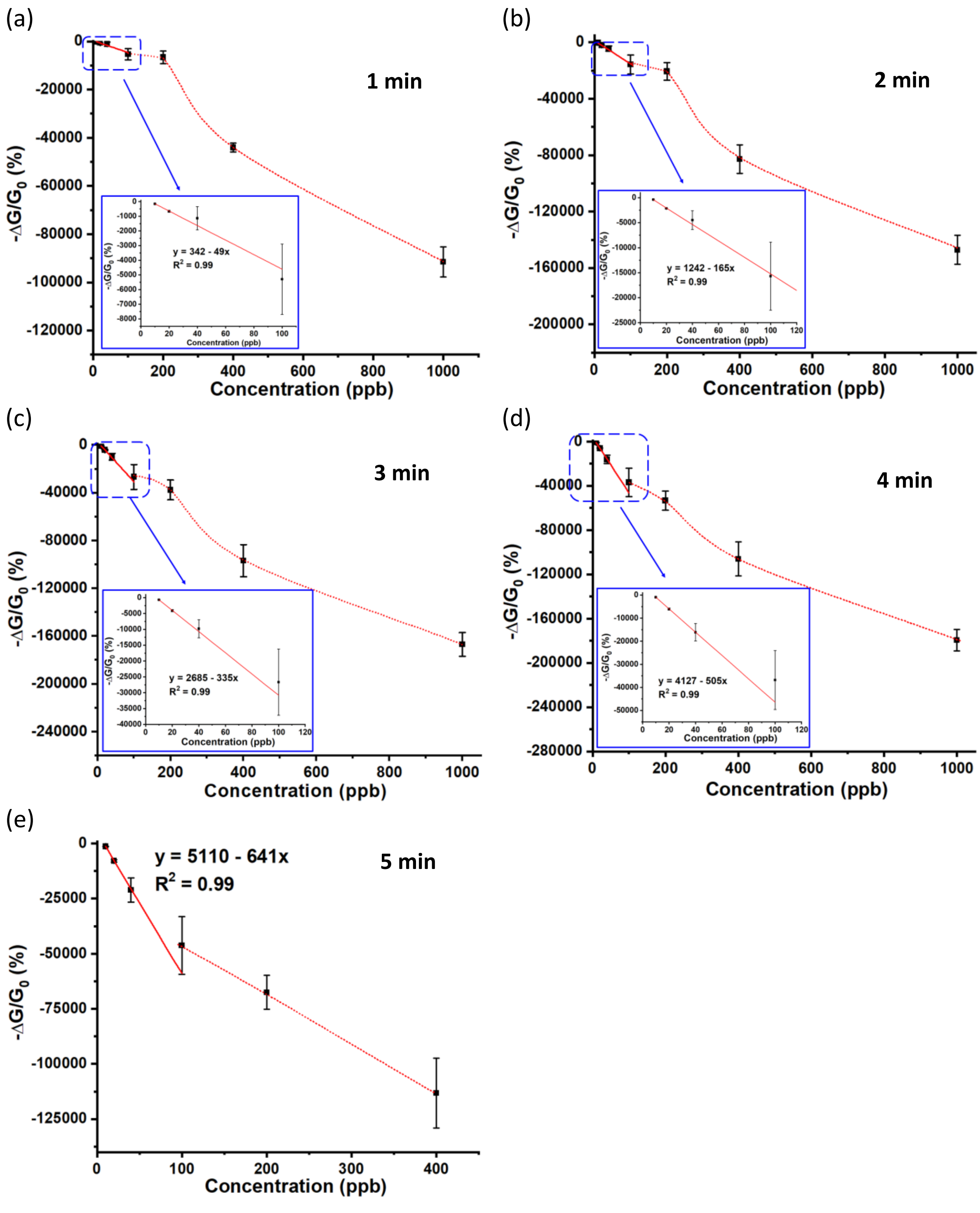

Figure S28. Response of **DC-100** device as a function of NO concentration. The values of response after the exposure for (a) 1, (b) 2, (c) 3, (d) 4, and (e) 5 min were plotted. The slopes of the curves are -49, -165, -335, -505, and -641. SSR is 8.009. N = 600, RMS is 0.1013.

Table S7. Calculated limits of detection for NO using **DC-100** under different exposure time.

Exposure time (min)	1	2	3	4	5
**LOD/ppt**	6.20	1.84	0.91	0.60	0.47

Table S8. Calculated degree of saturation after 5 minutes at various NO concentrations and the recovery percentage after 30 minutes in N_2 following 5 minutes of exposure to different NO concentrations.

Concentration (ppb)	10	20	40	100	200	400	1000
**Degree of saturation (%)**	0.70	4.29	12.18	25.19	36.83	62.23	100[a]
**Recovery percentage (%)**	83.37	83.00	85.40	92.02	92.36	92.31	95.73

[a] Saturation reached at 4.97 min.

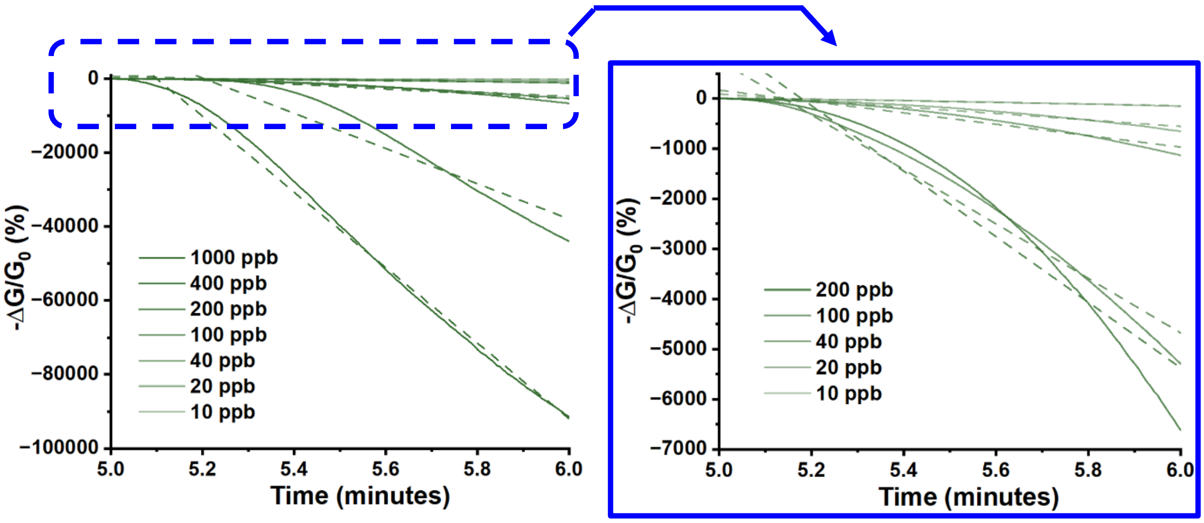

Figure S29. Responses of **DC-100** devices to 1000, 400, 200, 100, 40, 20, and 10 ppb of NO after 1 min of exposure and the linear fitting of the response.

Table S9. Linear fit equations and R^2 values for **DC-100** device responses to NO concentrations (1000, 400, 200, 100, 40, 20, and 10 ppb) after 1 min of exposure.

Concentration (ppb)	Linear fit equation	R^2 value
10	802 – 112x	0.98
20	3324 – 368x	0.95
40	5852 – 1137x	0.95
100	27960 – 5440x	0.96
200	33877 – 6544x	0.92
400	248170 – 47381x	0.91
1000	521730 – 102326x	0.99

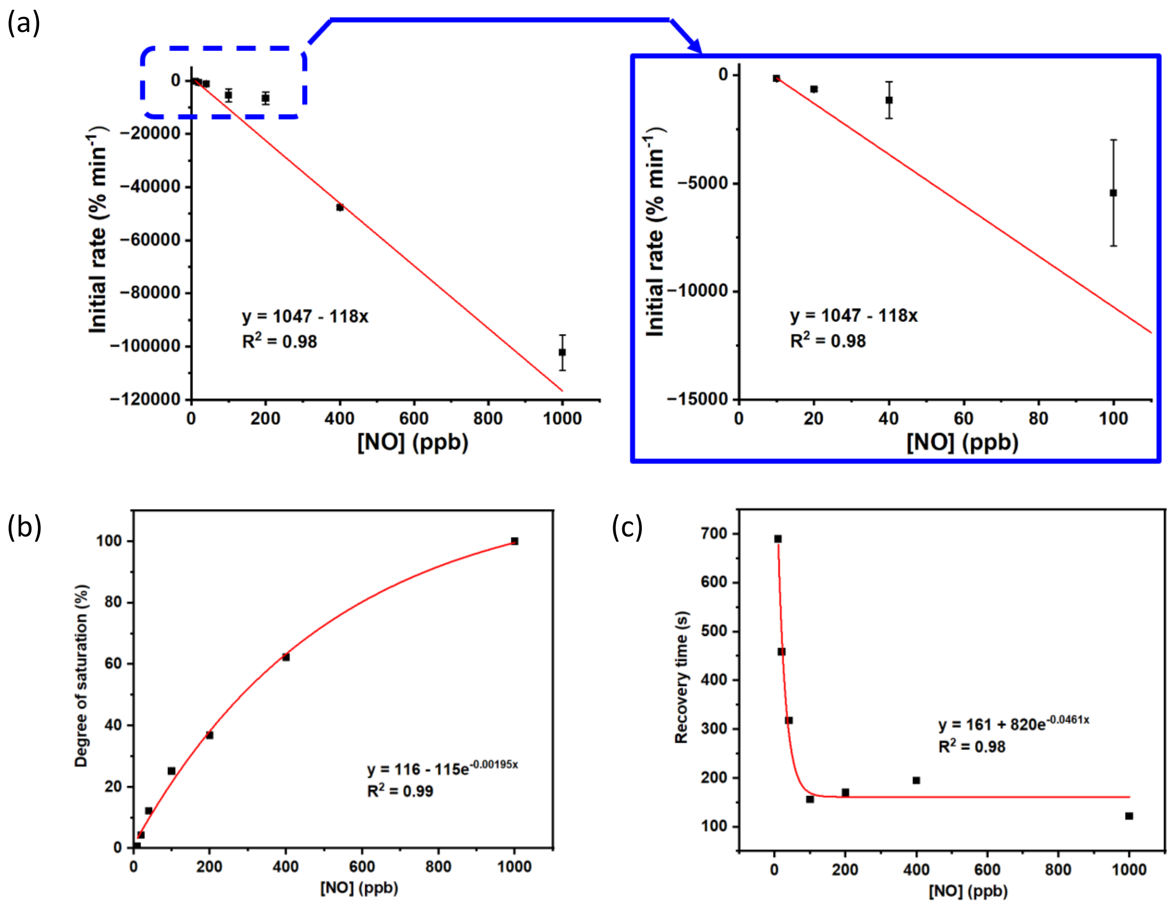

Figure S30. Plots of (a) the initial response rate, (b) the degree of saturation, and (c) the recovery time of **DC-100** as a function of NO concentration.

In Figure S30(b), the degree of saturation increases sharply from 10 to 400 ppb and fits an exponential saturation model, indicating rapid adsorption kinetics at low NO concentrations. This behavior is characteristic of pseudo-first-order kinetics with respect to NO concentration. The recovery time, defined as the time required for the sensor response to decay to 20% of its maximum value during desorption, remains stable (~150 s) at high concentrations (100–1000 ppb NO in N_2) but increases significantly at lower concentrations. Specifically, it rises to nearly 700 s at 10 ppb, as shown in Figure S30(c), suggesting slower desorption kinetics in the low-concentration regime.

12.4. Other gaseous analytes sensing performance

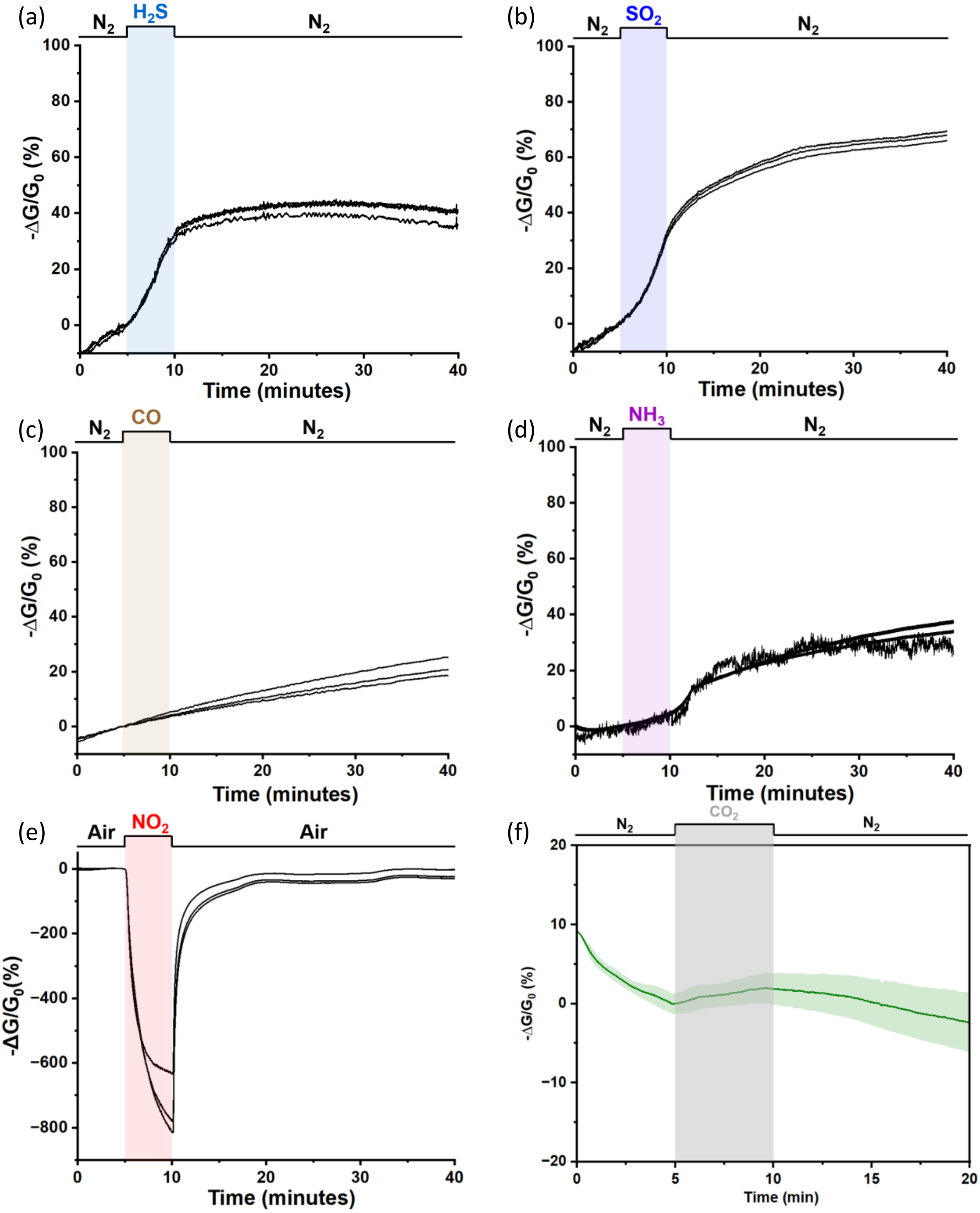

Figure S31. Responses of **DC-100** devices to 1 ppm of (a) H_2S, (b) SO_2, (c) CO, (d) NH_3, (e) NO_2, and (f) 4000 ppm CO_2 after 5 min. All experiments were conducted in dry N_2, except for NO_2, which was tested in dry air. For each test, 3 freshly prepared devices were used.

Although **DC-100** exhibits excellent selectivity toward NO, robust differentiation of analytes in complex gas mixtures may require an array of MTPz-based materials.

12.5. Representative examples of NO detection by MOFs and other nanomaterials/methods

Table S10. Examples of NO detection by MOFs and other nanomaterials/methods

Material	Sensing devices or method	Detection range	LOD	Response time (min)	Recovery (%)	No. of cycle with full sensitivity	Ref.
**DC-100**	Pc-based MOF chemiresistor	**10-1000 ppb**	**0.47-6.20 ppt**	**5**	**96% @ 1 ppm**	**5 @ 1 ppm**	**This work**
NiPc-Ni		20-1000 ppb	0.06-1.06 ppb	30	23% @ 1 ppm	-	(R7)
NiPc-Cu		20-1000 ppb	0.13-1 ppb	30	0% @ 1 ppm	-	(R7)
NiNPc-Ni		1 ppm	-	30	26% @ 1 ppm	-	(R7)
NiNPc-Cu		1 ppm	-	30	29% @ 1 ppm	-	(R7)
CoPc-Cu		10-80 ppm	-	30	30% @ 80 ppm	-	(R8)
COF-DC-8	Pc-based COF chemiresistor	20 ppb - 80 ppm	0.85-5.4 ppb	30	25% @ 80 ppm	0 @ 40 ppm	(R9)
NiPc-CoTAA	Pc-based MOF/COF hybrid chemiresistor	1 ppm	-	5	90% @ 1 ppm	-	(R10)
NiPc	Pc molecular film chemiresistor	5-500 ppm	-	20	94% @ 5 ppm	0 @ 50 ppm	(R11)
PbPc		20-100 ppm	-	25	92% @ 20 ppm	-	(R12)
$Cu_3(C_6O_6)_2$	MOF chemiresistor	1-40 ppm	12.8 ppb	10	53% @ 40 ppm	0 @ 40 ppm	(R13)
M_3HHTP_2–Graphene (M = Fe, Co, Ni, or Cu)		5-80 ppm	17 ppm	5	17%-30% @ 80 ppm	0 @ 80 ppm	(R14)
Ni_3HITP_2, Ni_3HHTP_2		5-80 ppm	0.16, 1.4 ppm	60	0%, 0% @ 5-80 ppm	-	(R15)
Bi(HHTP)		5-40 ppm	150 ppb	15	50% @ 40 ppm	-	(R16)
Pd–Pt/WO_3/p-Si/Al	chemiresistor	50-250 ppm	-	3	88% @ 200 ppm	0 @ 200 ppm	(R17)
ZnO		10-1000 ppb	10 ppb	1.5	15% @ 25 ppb	-	(R18)
functionalized carbon nanotubes		1-100 ppm	-	10	17% @ 100 ppb	0 @ 100 ppb	(R19)
SWCNTs/en-APTAS		100 ppb	-	10	90% @ 100 ppb	2 @ 100 ppb	(R20)
N-rGO/ZnO	p-n heterojunction	100-1000 ppb	100 ppb	10	97% @ 800 ppb	5 @ 800 ppb	(R21)
N-rGO/Pd	Field-effect transistors	2-420 ppb	2 ppb	4.5	43% @ 2 ppb	-	(R22)
CoTPP-polymer	Optical absorption spectroscopy	100-1000 ppb	33 ppb	30	94% @ 1 ppm	2 @ 1 ppm	(R23)
WO_3	potentiometric sensor array	1-100 ppb	-	15	70% @ 56 ppb	-	(R24)

Cytochrome c–doped xerogel	Spectroscopic	1-25 ppm	1 ppm	3.3	≈ 100% @ 20 ppm	-	(R25)
Cu(II) eriochrome cyanine R complex		0-6 ppm	0.23 ppm	10	≈ 100% @ 2 ppm	3 @ 2 ppm	(R26)
Griess assay (microgas analysis system)		0-200 ppb	7 ppb	≈13	-	-	(R27)
Substrate-integrated hollow waveguides		50-400 ppm	10 ppm	<1	-	-	(R28)
Photoacoustic spectroscopy		90-9000 ppm	11-41 ppb	Few seconds	-	-	(R29)
3,4-dichloroaniline and copper(II) bromide system	Gas Chromato-graphy	0-0.5 ppm	0.01 ppm	6	-	-	(R30)
Luminol (polypropylene hollow fiber membranes)	Chemi-luminescence	20-90 ppb	0.3 ppb	0.2	≈ 100% @ 20, 40, 60 ppb	-	(R31)
Photo-luminescent porous silicon		2-160 ppm	2 ppm	Few seconds	≈98% @ 11 ppm	-	(R32)
Diamino-fluorescein-2 (flow-based micro gas analysis system)		50-250 ppb	0.82-25 ppb	7	-	-	(R33)

13. XPS and EPR studies of NO exposure

For both XPS and EPR tests, 2 mg of **DC-100** was used for each analyte exposure sample. The analyte-exposed samples were prepared using a specific procedure. First, samples were evacuated using a home vacuum for 15 minutes. The container was then filled with 1 ppm NO gas until it reached atmospheric pressure. This evacuation-filling cycle was repeated three times in total. After the final filling, samples were kept in the 1 ppm NO environment for 2 hours. EPR tests were conducted immediately after exposure, using the operational conditions described in Section 7. For XPS tests, the analyte-exposed samples were stored under N_2 at room temperature until analysis. XPS measurements were performed under a pressure of approximately 10^{-9} psi.

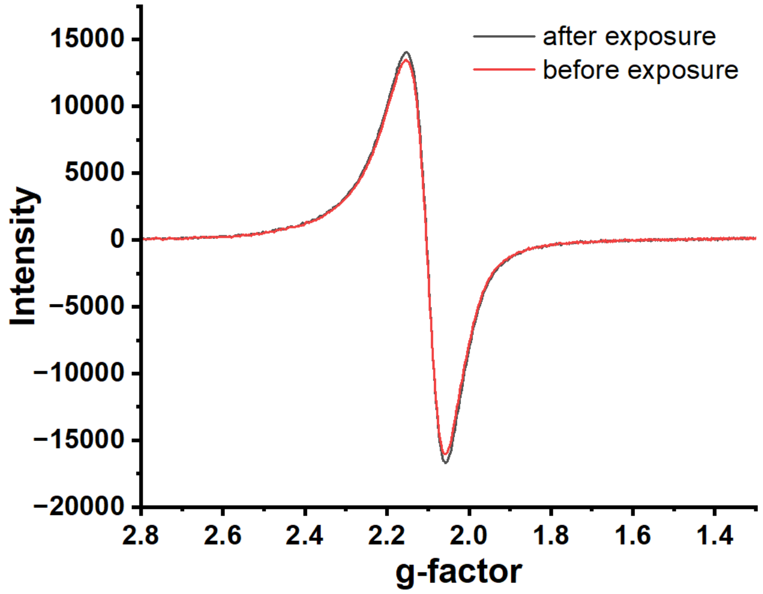

Figure S32. Comparison of the EPR of the pristine **DC-100** and **DC-100** at room temperature after 2-hour exposure of 1 ppm NO.

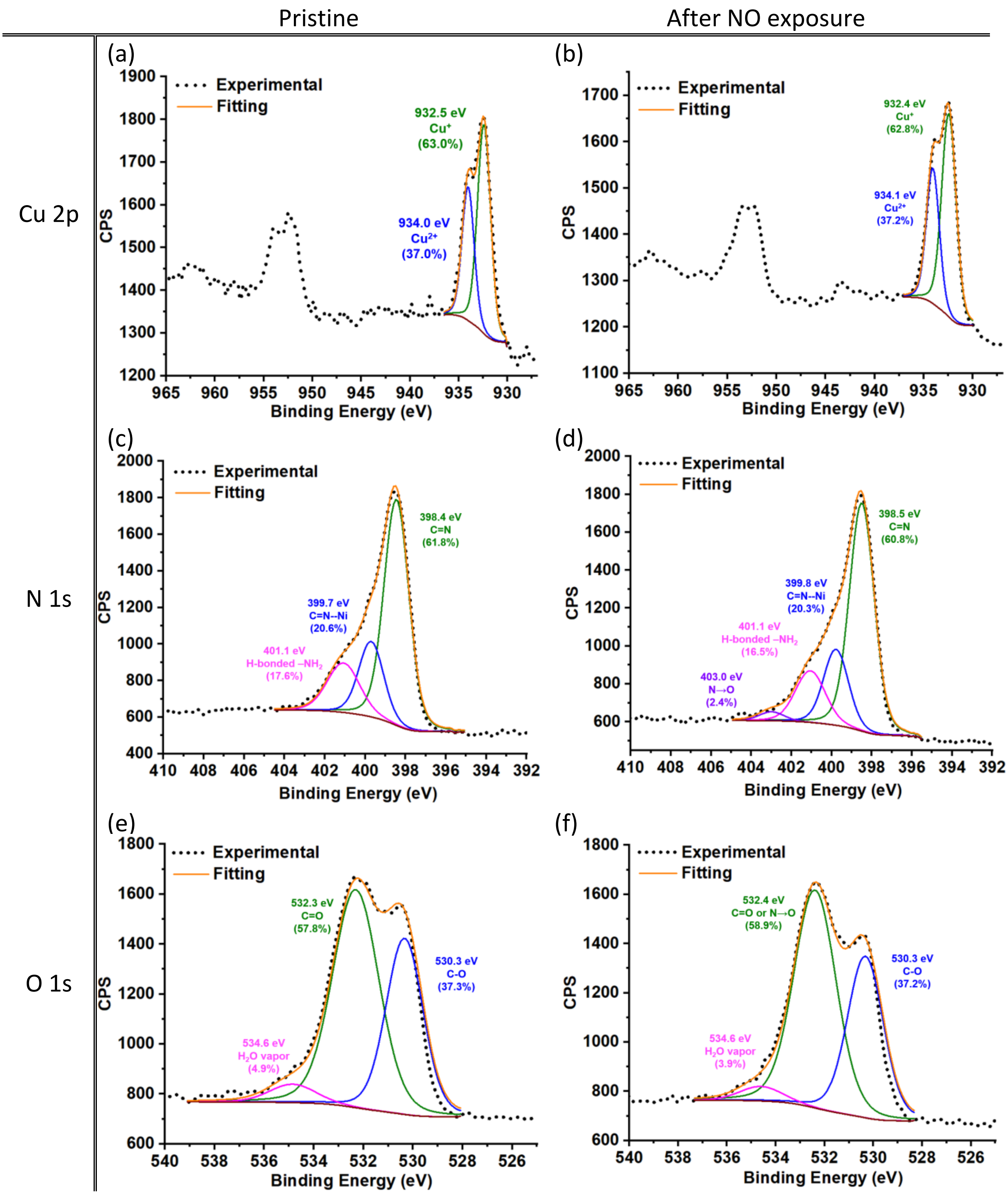

Figure S33. Comparison of the Cu2P, N1s, and O1s XPS spectra of pristine **DC-100** and **DC-100** after exposure to 1 ppm NO for 2 hours.

14. DRIFTS of NO exposure and N_2 recovery

DRIFTS data were collected on a Nicolet 6700 FT-IR spectrometer at room temperature. An environmental chamber was mounted within the DRIFTS housing. The chamber consisted of an air-tight steel domed volume that housed a sample cup. A gas inlet and a gas outlet port were affixed to the chamber with Swagelok fittings. The IR beam path accessed the sample cup through KBr windows. The sample cup within the environmental chamber was first loaded with a bed of dry KBr. A background spectrum of KBr was collected and used as the background for further experiments. Pristine **DC-100**, **DC-101**, and **DC-102** (2 mg) were mixed with KBr homogeneously by gently combining the two materials. The sample/KBr blend was then transferred to the sample cup and dried under N_2 stream at 110 ^oC. After 1 hour, a spectrum was collected under N_2 flow as the background for future experiments. Spectra were collected as a single beam (unsubtracted) and as the difference (subtracted) from a single beam of KBr. Single beam spectra were recorded as raw absorbance intensity across the spectral range accounting for contributions from KBr. Absorbance spectra were the result of subtracting the single beam obtained from pure KBr and that obtained from KBr blended with MOF. All spectra were collected at 32 scans with 4 cm^{-1} resolution from 400 cm^{-1} to 4000 cm^{-1}. After collecting an initial spectrum for the pristine sample, NO (1 % or 100 ppm in N_2) was introduced to the sample. Difference Spectra were collected continuously (around every 1 min). After the peaks reached to the maximum intensity (10 min for 1% NO and 15 min for 100 ppm NO), the NO flow was switched off, the difference spectra were continuously collected for 90 min (100 ppm NO) or 30 min (1% NO).

We used 1% NO and 100 ppm NO in the experiment for two main reasons: (1) 1 ppm NO, the highest concentration used in the sensing experiment, was too dilute to produce noticeable

spectroscopic changes in DRIFTS. Therefore, 100 ppm NO was selected as a compromise to ensure reliable spectroscopic evidence while maintaining relevant sensing conditions. (2) To compare the subtle spectroscopic changes among **DC-100**, **DC-101**, and **DC-102** when exposed to NO, a higher concentration (1%) was used to enhance the detection of these changes.

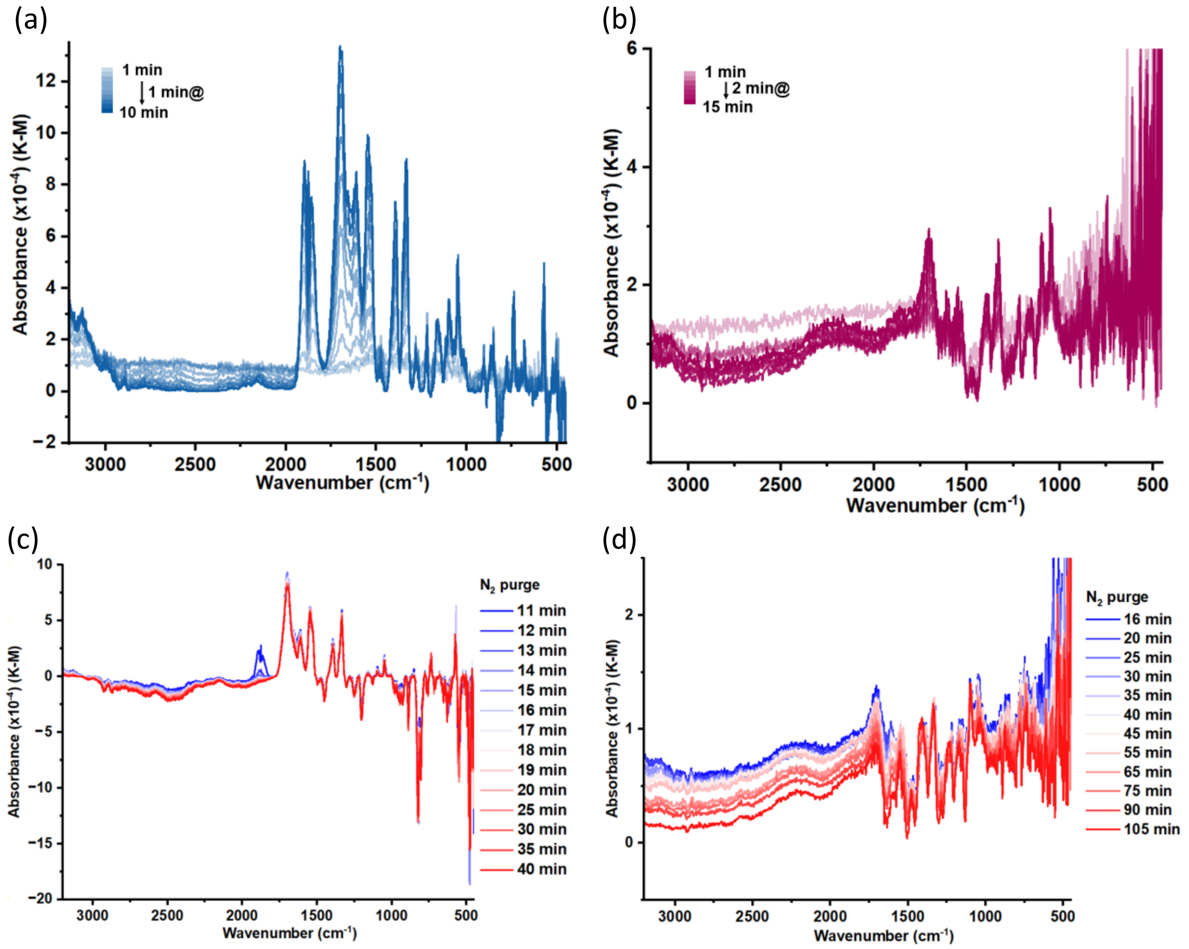

Figure S34. DRIFTS difference spectra of **DC-100** after continuous exposure to (a) 1% NO for 10 min and (b) 100 ppm of NO for 15 min. DRIFTS difference spectra of **DC-100** exposed to N_2 after the exposure of (c) 1% and (d) 100 ppm NO.

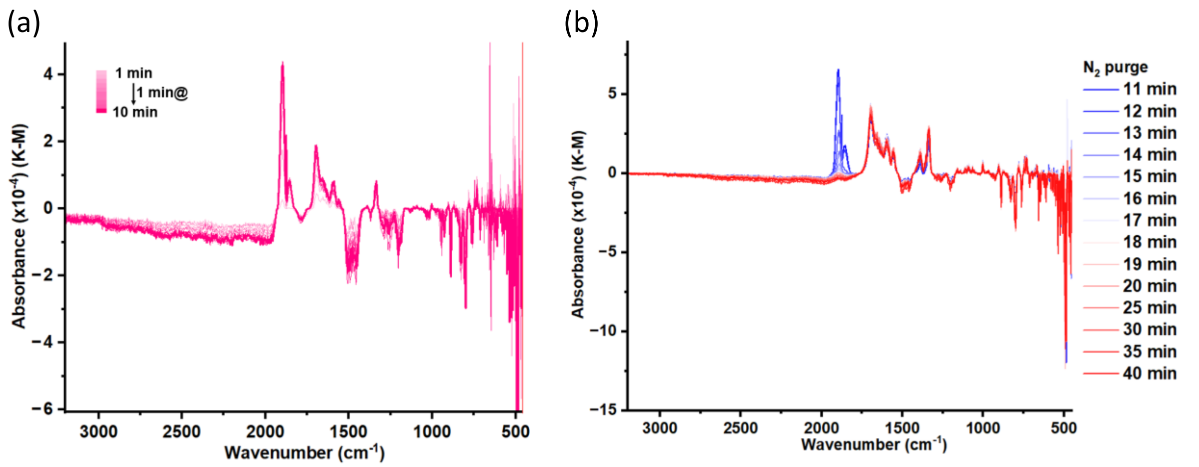

Figure S35. DRIFTS difference spectra of **DC-101** (a) after continuous exposure to 1% NO for 10 min and (b) exposed to N_2 after the exposure of 1% NO.

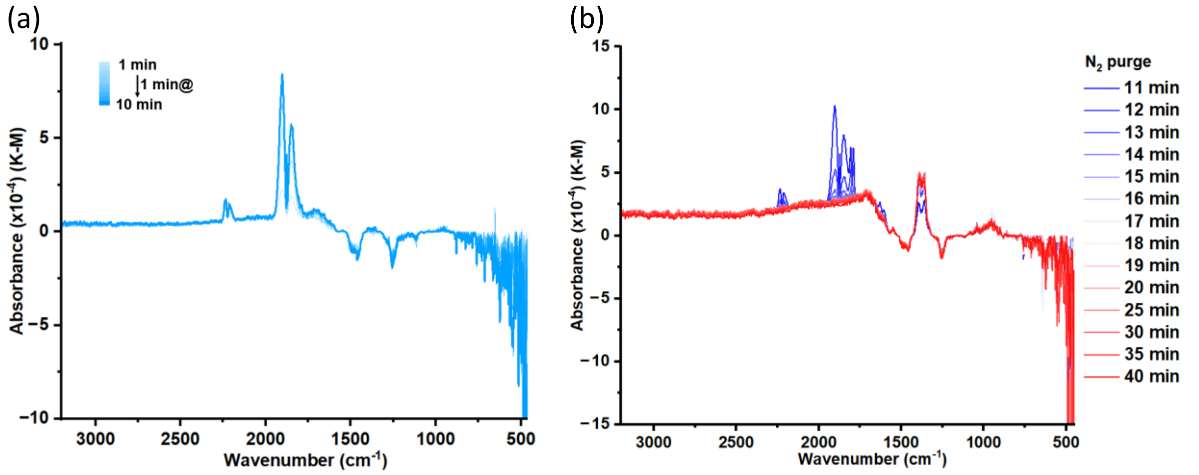

Figure S36. DRIFTS difference spectra of **DC-101** (a) after continuous exposure to 1% NO for 10 min and (b) exposed to N_2 after the exposure of 1% NO.

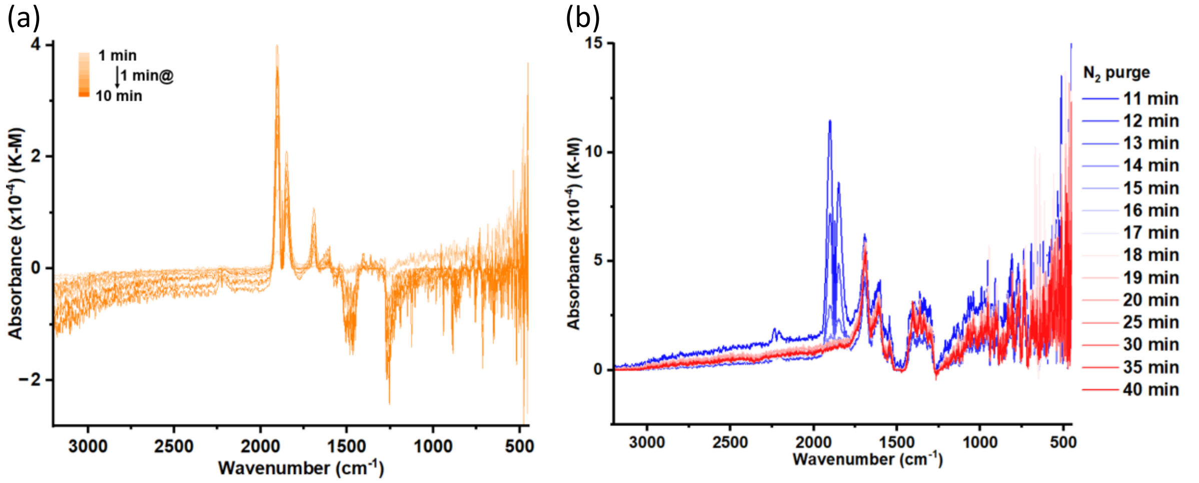

Figure S37. DRIFTS difference spectra of NiTPz-$(OH)_8$ (a) after continuous exposure to 1% NO for 10 min and (b) exposed to N_2 after the exposure of 1% NO.

15. Density functional theory experiments

A series of molecular models were investigated to confirm the spectral features observed in the periodic framework materials using the Gaussian09 software package.[R39] Molecular models included the **DC-100** monomer [NiTPz-$(OH)_8$], the **DC-100** monomer with hydroxyl protons progressively removed to furnish 1, 2, and 4 CO groups, the **DC-100** monomer with two removed protons and a single Cu atom coordinated by 2 CO groups from the monomer, and the **DC-100** monomer with two removed protons and a single Cu atom coordinated by both 2 CO groups from the monomer as well as ethylenediamine. In the single-metal molecules, NO was initialized at Ni. In the mixed-metal molecules, NO was initialized at both Ni and Cu. All molecules were geometrically optimized using the PBE hybrid exchange and correlation functional (PBE1PBE) including Grimme's empirical D3 dispersion corrections with Becke-Johnson damping (D3BJ) before a frequency calculation was performed on the optimized structure at the same level of theory. A def2svp basis was used with tight convergence and a superfine density grid. For thoroughness, the **DC-100** monomer with NO initialized at Ni was also optimized at the PBEh1PBE/def2svp, B3P86/def2tzvp, B3PW91/def2tzvp + D3BJ, PBE1PBE/def2svp + D3BJ, OLYP/def2tzvp, TPSSTPSS/def2tzvp + D3BJ, and TPSSh/def2tzvp levels of theory. These disparate models were employed due to the notorious difficulty of accurately reproducing metal-NO interactions with DFT.[R40] Notably, only the PBE1PBE/def2svp + D3BJ level of theory yielded a stable Ni-NO bond, whereas the rest of the models resulted in NO desorption. Therefore, this level of theory was applied to the remaining molecular models. For the **DC-100** monomer with NO bound to Ni, the NO vibrational frequency was 1986 cm^{-1}, near the free NO stretch and suggestive of weak bonding. For the **DC-100** monomer with one proton removed to furnish 1 CO group, the Ni-NO bond

was similarly weak and resulted in an NO vibrational frequency of 1988 cm^{-1}. For all other proton-deficient single-metal monomers, the NO molecule desorbed. When Cu(I) was included by coordination to 2 CO groups but without ethylenediamine, a slightly stronger Cu-NO bond formed with an NO vibrational frequency of 1893 cm^{-1}, still far from the experimentally-observed feature near 1700 cm^{-1}. However, when the molecular model incorporated ethylenediamine to yield 4-coordinate Cu(I), NO formed a stronger bond to the Cu atom and the resulting NO vibrational frequency was 1693 cm^{-1}, in excellent agreement with the NO vibrational mode obtained in the corresponding periodic model with VASP (1685 cm^{-1}).

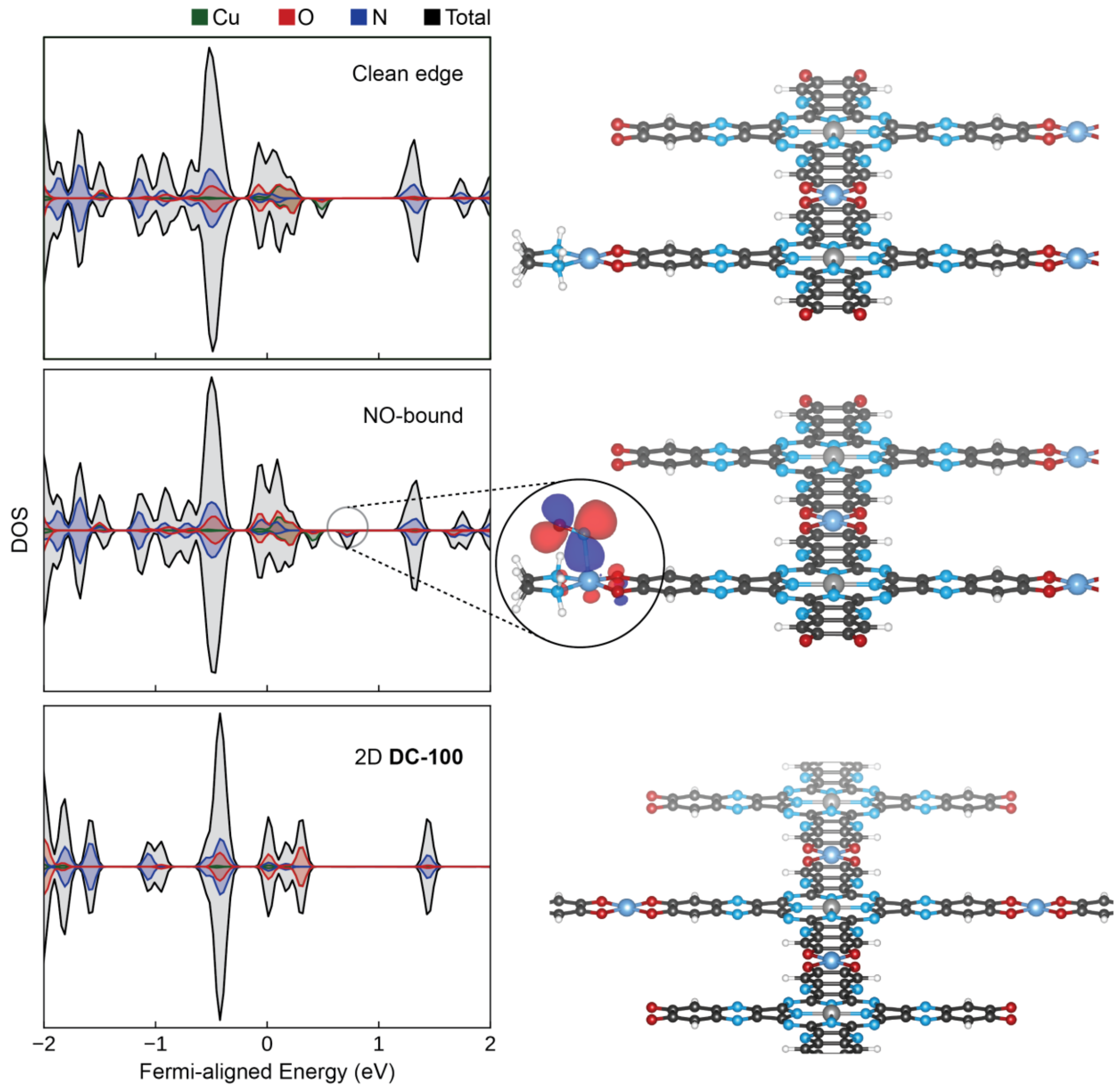

Figure S38. Density of states plotted for the edge-site Cu(I) model before and after NO binding, showing the appearance of unoccupied antibonding N-O states ~0.7 eV above the Fermi level while conserving the other features. The real space wavefunction is plotted for the band containing the new states. The DOS of single-layer periodic **DC-100** is included for comparison.

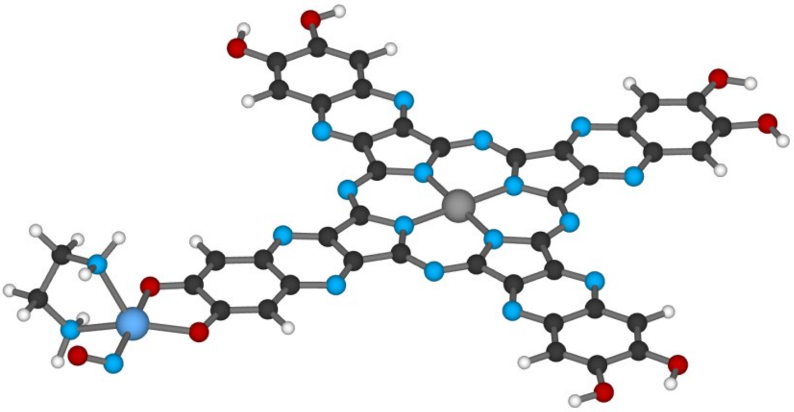

Figure S39. Molecular model of **DC-100** composed of the NiTPz-$(OH)_8$ monomer with two protons removed to form 2 CO groups, a single Cu atom, and coordinating ethylenediamine. NO bonds to the Cu(I) atom and vibrates at a frequency of 1693 cm^{-1}.

16. References

(R1) Zhou, Z.-L.; Weber, E.; Keana, J. F., Acetoxylation of 6, 7-dialkoxy-substituted 1, 4 dihydroquinoxaline-2, 3-diones (Qxs) using fuming nitric acid in acetic acid: A facile synthesis of 5-acyloxy-6, 7-dialkoxy QXs. *Tetrahedron letters* **1995,** *36* (42), 7583-7586.

(R2) Ottenwaelder, X.; Ruiz-García, R.; Blondin, G.; Carasco, R.; Cano, J.; Lexa, D.; Journaux, Y.; Aukauloo, A. From Metal to Ligand Electroactivity in Nickel(II) Oxamato Complexes. *Chem. Commun.* **2004**, 5, 504-505.

(R3) (a) Schroder, D. K. *Semiconductor Material and Device Characterization*. John Wiley & Sons, Inc.: 2005. (b) Smits, F. M. Measurement of Sheet Resistivities with the Four-Point Probe. *Bell System Technical Journal* **1958**, *37*, 711-718.

(R4) (a) Li, J.; Lu, Y.; Ye, Q.; Cinke, M.; Han, J.; Meyyappan, M. Carbon Nanotube Sensors for Gas and Organic Vapor Detection. *Nano Lett.* **2003**, *3*, 929. S20. (b) Ammu, S.; Dua, V.; Agnihotra, S. R.; Surwade, S. P.; Phulgirkar, A.; Patel, S.; Manohar, S. K. Flexible, All-Organic Chemiresistor for Detecting Chemically Aggressive Vapors. *J. Am. Chem. Soc*. **2012**, *134*, 4553-4556.

(R5) Samarghandi, M. R.; Hadi, M.; McKay, G. Breakthrough Curve Analysis for Fixed-Bed Adsorption of Azo Dyes Using Novel Pine Cone—Derived Active Carbon. *Adsorption Science & Technology* **2014**, *32*, 791-806.

(R6) (a) Garcia, S.; Gil, M. V.; Martin, C. F.; Pis, J. J.; Rubiera, F.; Pevida, C. Breakthrough Adsorption Study of a Commercial Activated Carbon for Pre-Combustion CO_2 Capture. *Chem. Eng. J*. **2011**, *171*, 549-556; (b) England, C.; Corcoran, W. H. The Rate and Mechanism of the Air Oxidation of Parts-per-Million Concentrations of Nitric Oxide in the Presence of Water Vapor. *Ind. Eng. Chem. Fundam.* **1975**, *14*, 55–63.

(R7) Meng, Z.; Aykanat, A.; Mirica, K. A. Welding Metallophthalocyanines into Bimetallic Molecular Meshes for Ultrasensitive, Low-Power Chemiresistive Detection of Gases. *J. Am. Chem. Soc.* **2019**, *141* (5), 2046-2053.

(R8) Aykanat, A.; Meng, Z.; Stolz, R. M.; Morrell, C. T.; Mirica, K. A. Bimetallic Two-dimensional Metal–Organic Frameworks for the Chemiresistive Detection of Carbon Monoxide. *Angew. Chem. Int. Ed.* **2022**, *61* (6), e202113665.

(R9) Meng, Z.; Stolz, R. M.; Mirica, K. A. Two-Dimensional Chemiresistive Covalent Organic Framework with High Intrinsic Conductivity. *J. Am. Chem. Soc.* **2019**, *141* (30), 11929-11937.

(R10) Yue, Y.; Cai, P.; Xu, X.; Li, H.; Chen, H.; Zhou, H.-C.; Huang, N. Conductive Metallophthalocyanine Framework Films with High Carrier Mobility as Efficient Chemiresistors. *Angew. Chem. Int. Ed.* **2021**, *60* (19), 10806-10813.

(R11) Ho, K.-C.; Tsou, Y.-H. Chemiresistor-Type NO Gas Sensor Based on Nickel Phthalocyanine Thin Films. *Sens. Actuators B Chem.* **2001**, *77* (1–2), 253-259.

(R12) Ho, K.-C.; Chen, C.-M.; Liao, J.-Y. Enhancing Chemiresistor-Type NO Gas-Sensing Properties Using Ethanol-Treated Lead Phthalocyanine Thin Films. *Sens. Actuators B Chem.* **2005**, *108* (1–2), 418-426.

(R13) Meng, Z.; Stolz, R. M.; De Moraes, L. S.; Jones, C. G.; Eagleton, A. M.; Nelson, H. M.; Mirica, K. A. Gas-induced Electrical and Magnetic Modulation of Two-dimensional Conductive Metal–Organic Framework. *Angew. Chem. Int. Ed.* **2024**, *63* (24), e202404290.

(R14) Ko, M.; Aykanat, A.; Smith, M. K.; Mirica, K. A. Drawing Sensors with Ball-Milled Blends of Metal–Organic Frameworks and Graphite. *Sensors* **2017**, *17*, 2192 (1-17).

(R15) Smith, M. K.; Mirica, K. A. Self-Organized Frameworks on Textiles (SOFT): Conductive Fabrics for Simultaneous Sensing, Capture, and Filtration of Gases. *J. Am. Chem. Soc.* **2017**, *139*, 16759-16767.

(R16) Aykanat, A.; Jones, C. G.; Cline, E.; Stolz, R. M.; Meng, Z.; Nelson, H. M.; Mirica, K. A. Conductive Stimuli-Responsive Coordination Network Linked with Bismuth for Chemiresistive Gas Sensing. *ACS Appl. Mater. Interfaces* **2021**, *13* (50), 60306-60318.

(R17) Zhang, W.; Uchida, H.; Katsube, T.; Nakatsubo, T.; Nishioka, Y. A Novel Semiconductor NO Gas Sensor Operating at Room Temperature. *Sens. Actuators B Chem.* **1998**, *49* (1–2), 58-62.

(R18) Singh, P.; Hu, L.-L.; Zan, H.-W.; Tseng, T.-Y. Highly Sensitive Nitric Oxide Gas Sensor Based on ZnO-Nanorods Vertical Resistor Operated at Room Temperature. *Nanotechnology* **2019**, *30* (9), 095501 (1-7).

(R19) Jeong, D.-W.; Kim, K. H.; Kim, B. S.; Byun, Y. T. Characteristics of Highly Sensitive and Selective Nitric Oxide Gas Sensors Using Defect-Functionalized Single-Walled Carbon Nanotubes at Room Temperature. *Appl. Surf. Sci.* **2021**, *550* (149250), 149250 (1-7).

(R20) Lim, N.; Kim, K. H.; Byun, Y. T. Preparation of Defected SWCNTs Decorated with En-APTAS for Application in High-Performance Nitric Oxide Gas Detection. *Nanoscale* **2021**, *13* (13), 6538-6544.

(R21) Qiu, J.; Hu, X.; Min, X.; Quan, W.; Tian, R.; Ji, P.; Zheng, H.; Qin, W.; Wang, H.; Pan, T.; Cheng, S.; Chen, X.; Zhang, W.; Wang, X. Observation of Switchable Dual-Conductive Channels and Related Nitric Oxide Gas-Sensing Properties in the N-rGO/ZnO Heterogeneous Structure. *ACS Appl. Mater. Interfaces* **2020**, *12* (17), 19755-19767.

(R22) Li, W.; Geng, X.; Guo, Y.; Rong, J.; Gong, Y.; Wu, L.; Zhang, X.; Li, P.; Xu, J.; Cheng, G.; Sun, M.; Liu, L. Reduced Graphene Oxide Electrically Contacted Graphene Sensor for Highly Sensitive Nitric Oxide Detection. *ACS Nano* **2011**, *5* (9), 6955-6961.

(R23) Shiba, S.; Yamada, K.; Matsuguchi, M. Humidity-Resistive Optical NO Gas Sensor Devices Based on Cobalt Tetraphenylporphyrin Dispersed in Hydrophobic Polymer Matrix. *Sensors* **2020**, *20* (5), 1295 (1-8).

(R24) Mondal, S. P.; Dutta, P. K.; Hunter, G. W.; Ward, B. J.; Laskowski, D.; Dweik, R. A. Development of High Sensitivity Potentiometric NOx Sensor and Its Application to Breath Analysis. *Sens. Actuators, B: Chem*. **2011**, *158*, 292-298.

(R25) Aylott, J. W.; Richardson, D. J.; Russell, D. A. Optical Biosensing of Gaseous Nitric Oxide Using Spin-Coated Sol−Gel Thin Films. *Chem. Mater.* **1997**, *9* (11), 2261–2263.

(R26) Dacres, H.; Narayanaswamy, R. A New Optical Sensing Reaction for Nitric Oxide. *Sens. Actuators B Chem.* **2003**, *90* (1–3), 222–229.

(R27) Toda, K.; Hato, Y.; Ohira, S.-I.; Namihira, T. Micro-Gas Analysis System for Measurement of Nitric Oxide and Nitrogen Dioxide: Respiratory Treatment and Environmental Mobile Monitoring. *Anal. Chim. Acta* **2007**, *603* (1), 60–66.

(R28) Petruci, J. F. da S.; Tütüncü, E.; Cardoso, A. A.; Mizaikoff, B. Real-Time and Simultaneous Monitoring of NO, NO2, and N2O Using Substrate-Integrated Hollow Waveguides Coupled to a Compact Fourier Transform Infrared (FT-IR) Spectrometer. *Appl. Spectrosc.* **2019**, *73* (1), 98–103.

(R29) Gondal, M. A.; Khalil, A. A. I.; Al-Suliman, N. High Sensitive Detection of Nitric Oxide Using Laser Induced Photoacoustic Spectroscopy at 213 Nm. *Appl. Opt.* **2012**, *51* (23), 5724–5734.

(R30) Funazo, K.; Tanaka, M.; Shono, T. Gas Chromatographic Determination of Nitric Oxide at Sub-Ppm Levels. *Anal. Chim. Acta* **1980**, *119* (2), 291–297.

(R31) Robinson, J. K.; Bollinger, M. J.; Birks, J. W. Luminol/H2O2 Chemiluminescence Detector for the Analysis of Nitric Oxide in Exhaled Breath. *Anal. Chem.* **1999**, *71* (22), 5131–5136.

(R32) Harper, J.; Sailor, M. J. Detection of Nitric Oxide and Nitrogen Dioxide with Photoluminescent Porous Silicon. *Anal. Chem.* **1996**, *68* (21), 3713–3717.

(R33) Toda, K.; Koga, T.; Kosuge, J.; Kashiwagi, M.; Oguchi, H.; Arimoto, T. Micro Gas Analyzer Measurement of Nitric Oxide in Breath by Direct Wet Scrubbing and Fluorescence Detection. *Anal. Chem.* **2009**, *81* (16), 7031–7037.

(R34) Liu, Y.; Li, S.; Dai, L.; Li, J.; Lv, J.; Zhu, Z.; Yin, A.; Li, P.; Wang, B. The Synthesis of Hexaazatrinaphthylene-based 2D Conjugated Copper Metal-organic Framework for Highly Selective and Stable Electroreduction of CO_2 to Methane. *Angew. Chem. Int. Ed.* **2021**, *60* (30), 16409–16415.

(R35) Ghodselahi, T.; Vesaghi, M. A.; Shafiekhani, A.; Baghizadeh, A.; Lameii, M. XPS Study of the Cu@Cu2O Core-Shell Nanoparticles. *Appl. Surf. Sci.* **2008**, *255* (5), 2730–2734.

(R36) (a) Wu, S.; Wen, G.; Schlogl, R.; Su, D. S. Carbon Nanotubes Oxidized by a Green Method as Efficient Metal-Free Catalysts for Nitroarene Reduction. *Phys. Chem. Chem. Phys*. **2015**, *17*, 1567-1571. (b) Qi, W.; Liu, W.; Zhang, B.; Gu, X.; Guo, X.; Su, D. Oxidative Dehydrogenation on Nanocarbon: Identification and Quantification of Active Sites by Chemical Titration. *Angew. Chem. Int. Ed*. **2013**, *52*, 14224–14228.

(R37) de Oteyza, D. G.; El-Sayed, A.; Garcia-Lastra, J. M.; Goiri, E.; Krauss, T. N.; Turak, A.; Barrena, E.; Dosch, H.; Zegenhagen, J.; Rubio, A.; Wakayama, Y.; Ortega, J. E.

Copper-Phthalocyanine Based Metal–Organic Interfaces: The Effect of Fluorination, the Substrate, and Its Symmetry. *J. Chem. Phys.* **2010**, *133* (21), 214703 (1-6).

(R38) Graf, N.; Yegen, E.; Gross, T.; Lippitz, A.; Weigel, W.; Krakert, S.; Terfort, A.; Unger, W. E. S. XPS and NEXAFS Studies of Aliphatic and Aromatic Amine Species on Functionalized Surfaces. *Surf. Sci.* **2009**, *603* (18), 2849–2860.

(R39) Frisch, M. J. et al. *Gaussian 09, Revision E.01*, Gaussian Inc. 2013.

(R40) Boguslawski, K.; Jacob, C. R.; Reiher, M. Can DFT Accurately Predict Spin Densities? Analysis of Discrepancies in Iron Nitrosyl Complexes. *J. Chem. Theory Comput.* **2011**, *7* (9), 2740–2752.
